# Statin Therapy Independently Reduces Mortality and Liver Complications in Patients With Cirrhosis: An Updated Systematic Review and Meta‐Analysis

**DOI:** 10.1111/apt.70526

**Published:** 2026-01-10

**Authors:** Bernardo de Faria Moraes, Gustavo André Pedral Diniz Leite, Igor Boechat Silveira, Gabriel André Pedral Diniz Leite, Maria Luisa Motta Fonseca, Leonardo Corrêa Suffert, Luisa Medeiros Visentini, Luis Pedro Possapp Beis, Guilherme Grossi Lopes Cançado

**Affiliations:** ^1^ Universidade Federal de Minas Gerais Belo Horizonte Brazil; ^2^ Universidade Federal de Juiz de Fora Juiz de Fora Brazil; ^3^ Pontifícia Universidade Católica do Rio Grande do Sul Porto Alegre Brazil; ^4^ Universidade do Oeste Paulista Presidente Prudente Brazil; ^5^ Hospital das Clínicas da Universidade Federal de Minas Gerais Belo Horizonte Brazil; ^6^ Hospital da Polícia Militar de Minas Gerais Belo Horizonte Brazil

**Keywords:** cirrhosis, hepatocellular carcinoma, mortality, portal hypertension, statins

## Abstract

**Background:**

The role of statins in cirrhosis remains controversial. Historically restricted due to safety concerns, emerging evidence highlights potential pleiotropic benefits, though effects on mortality and decompensation remain uncertain.

**Aims:**

To evaluate the effects of statin therapy on all‐cause mortality, hepatic decompensation, hepatic venous pressure gradient (HVPG), and hepatocellular carcinoma (HCC) in cirrhosis.

**Methods:**

We systematically searched major databases until July 2025 for randomised controlled trials (RCTs) and observational studies comparing statin therapy versus non‐use in cirrhosis. Random‐effects meta‐analyses were performed.

**Results:**

The meta‐analysis included 25 studies (9 RCTs, 16 observational) with 81,992 patients. Statins reduced all‐cause mortality in the overall analysis (unadjusted odds ratio 0.59; 95% CI: 0.48–0.71) and in RCTs (odds ratio 0.45; 95% CI 0.25–0.82), supported by a significant HVPG reduction. Conversely, statins reduced hepatic decompensation in the overall analysis (unadjusted odds ratio 0.56; 95% CI: 0.47–0.66) but not in RCTs (odds ratio 0.75; 95% CI: 0.52–1.09). Observational data indicated a protective association for HCC (adjusted hazard ratio 0.61; 95% CI: 0.46–0.82), and no RCT reported this outcome.

**Conclusions:**

Statin therapy is associated with improved survival in cirrhosis, supported mechanistically by reductions in portal pressure. Observational evidence suggests benefits for decompensation and HCC, though these remain uncertain due to residual confounding. Large‐scale, long‐term RCTs are needed to clarify the role of statins as disease‐modifying therapy in cirrhosis.

## Graphical abstract

Statin therapy in cirrhosis may confer a survival benefit while reducing the risk of hepatic decompensation and hepatocellular carcinoma.

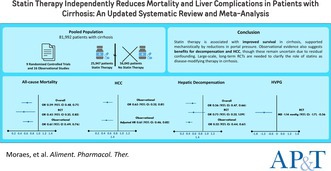

## 1 Introduction

Liver cirrhosis remains a major global health burden, responsible for nearly 2 million deaths annually and accounting for approximately 4% of all global mortality. Most of these deaths arise from complications of portal hypertension and the development of hepatocellular carcinoma (HCC) [1]. Despite advances in supportive care and research, there is no established pharmacological therapy that reverses hepatic fibrosis or consistently prevents cirrhosis‐related complications, apart from addressing the underlying aetiology. Consequently, patients remain vulnerable to progressive disease and adverse outcomes.

Statins, or 3‐hydroxy‐3‐methylglutaryl coenzyme A (HMG‐CoA) reductase inhibitors, have attracted increasing interest for their pleiotropic effects in cirrhosis, including anti‐inflammatory, anti‐fibrotic, antioxidative and endothelial‐modulating properties [2]. Owing to their well‐established benefits in cardiovascular disease, statins are among the most widely prescribed medications worldwide, with an estimated 25% of adults over the age of 40 receiving therapy [3]. Although the Baveno VII consensus supports their use in patients with cirrhosis who have conventional indications, their application in this population remains inconsistent, largely due to the absence of consensus on their potential benefits independent of cardiovascular comorbidities and safety concerns. While statins are generally considered safe across the spectrum of advanced chronic liver disease, cautious use and dose adjustments are recommended in patients with decompensated cirrhosis due to altered pharmacokinetics [4].

Earlier systematic reviews suggested that statins may attenuate fibrosis progression, reduce the risk of decompensation, HCC, and improve survival; however, these conclusions were based on a limited number of randomised controlled trials (RCTs) and observational studies [5, 6]. Since then, an expanding body of evidence from both RCTs and large‐scale observational cohorts has strengthened this association. More recently, a 2025 meta‐analysis reported a significant reduction in portal pressure with statin therapy but found no significant effect on mortality, ascites, or variceal bleeding [7]. However, this analysis was restricted to RCTs with predominantly short‐term follow‐up and did not include any observational data, leaving the cumulative impact on the long‐term clinical endpoints uncertain. Given these advances, a comprehensive reassessment is warranted. We therefore conducted a systematic review and meta‐analysis to evaluate the effects of statin therapy on key clinical outcomes in cirrhosis, including HCC development, hepatic decompensation, and all‐cause mortality. We aimed to synthesise the best available evidence to inform clinical practice and guideline development, recognising that large, long‐term randomised controlled trials are still ongoing.

## 2 Methods

This systematic review and meta‐analysis was performed in accordance with the Preferred Reporting Items for Systematic Reviews and Meta‐Analyses (PRISMA) [8]. The study protocol was registered in PROSPERO (CRD420251143472).

### 2.1 Eligibility Criteria

Eligible randomised controlled trials (RCTs) and observational studies that met all the inclusion criteria were included in this systematic review: (I) patients with liver cirrhosis, (II) patients on statin therapy in one study arm, (III) at least one of the following outcomes of interest was reported: mortality, variceal bleeding, spontaneous bacterial peritonitis (SBP), hepatorenal syndrome (HRS), ascites, hepatic encephalopathy (HE), hepatic venous pressure gradient (HVPG) or HCC. Exclusion criteria encompassed: (I) absence of a control group, (II) studies conducted on non‐cirrhotic patients, (III) studies conducted exclusively on populations of cirrhotic patients with HCC, (IV) studies in which statins were administered only in combination with another active intervention versus placebo or no treatment, as the independent effect of statin therapy could not be isolated, (V) studies that did not report any of the prespecified outcomes of interest, and (VI) abstracts, reviews, case studies and letters.

### 2.2 Search Strategy

A systematic search of PubMed, Embase and Cochrane Central Register of Controlled Trials (CENTRAL) was conducted from inception to July 11, 2025. There were no restrictions on language or date. Reference lists of included studies and relevant previous reviews were manually screened to identify additional eligible studies. Detailed information on search strategies is available in eTable 1 in Data S1.

### 2.3 Study Selection

All screened studies were uploaded to the Rayyan online review platform [9]. After removing duplicates, two independent investigators (B.F.M. and I.B.S.) assessed the eligibility of studies in two stages: (I) title and abstract, (II) full‐text review. Discrepancies in study selection were resolved by consensus or, when necessary, through consultation with a third reviewer (G.G.L.C.).

### 2.4 Data Collection

Using a standardised data extraction form, two reviewers (B.F.M. and I.B.S.) independently extracted data from each included study. Any discrepancies were resolved by consensus or, if necessary, through consultation with a third reviewer (G.G.L.C.). Extracted data included: (1) study characteristics; (2) study design; (3) demographic data; (4) clinical details of the population; (5) specifics of the intervention and comparator; (6) reported outcomes; and (7) the number of events for each outcome of interest and the total population analysed.

### 2.5 Risk of Bias Assessments

Risk of bias was assessed independently for each study by two reviewers (Gu.A.P.D.L and Ga.A.P.D.L) with the Cochrane Risk of Bias tool—RoB 2 for RCTs [10] and with the Cochrane Risk of Bias tool—ROBINS‐I V2 for observational studies [11]. The risk of bias was performed for all evaluated outcomes. Discrepancies were resolved through consensus or, when required, by consultation with a third reviewer (G.G.L.C.).

### 2.6 Outcomes

The primary outcomes were all‐cause mortality, hepatic decompensation, and HCC. The secondary outcomes were the change in the HVPG and the individual components of decompensation: variceal bleeding, SBP, HRS, ascites, and HE. Detailed definitions for the assessed outcomes are provided in eText 1 (Data S1).

### 2.7 Data Synthesis

Meta‐analysis of primary and secondary outcomes was performed using a random‐effects model with restricted maximum‐likelihood (REML) estimation for between‐study variance. To ensure methodological consistency across all study designs, particularly given the inclusion of case–control studies, odds ratios (ORs) with 95% confidence intervals (CIs) were used as the primary summary statistic for all meta‐analyses. This applied to the overall pooled analyses combining RCTs and observational studies, as well as analyses stratified by study design. To minimise confounding, data from propensity score‐matched populations were prioritised for dichotomous outcomes whenever available for the observational studies. For the continuous outcome of change in HVPG, the effect size measured was mean difference (MD) with corresponding 95% confidence intervals for RCTs only. To handle incompletely reported continuous data from the included trials, two standard imputation methods were applied. First, for studies reporting medians and interquartile ranges, we estimated the means and standard deviations using the validated method described by Wan et al. [12]. Second, for studies that provided a mean change from baseline but omitted its corresponding standard deviation, this value was derived from the reported *p*‐value. The confidence interval for all summary effects was calculated with the Wald‐type method. Meta‐analysis of adjusted HRs was performed for primary outcomes of observational studies using a random‐effects model with restricted maximum‐likelihood (REML) estimation for between‐study variance. Study‐specific adjusted HRs and confidence intervals were log‐transformed for analysis, with standard errors derived from the confidence interval limits. The pooled summary hazard ratio (HR) and confidence interval were calculated using inverse‐variance weighting under the random‐effects model. This analysis was restricted to studies reporting adjusted HRs; adjusted ORs or RRs were not included or converted for this synthesis. Meta‐analysis of adjusted HRs was not conducted for secondary outcomes (individual decompensation events) because the observational studies did not provide sufficient adjusted HR data to permit a formal pooled analysis. Sensitivity analyses were conducted to assess the robustness of the findings. First, acknowledging that relative risk (RR) is often more intuitive for clinical interpretation, we conducted a sensitivity analysis using RR restricted to the RCTs; this analysis was not performed for the observational data due to the inclusion of case–control studies, from which RR cannot be validly estimated. To determine if effects varied over time, subgroup comparisons stratified by follow‐up duration (short‐term [≤ 3 months] vs. long‐term [> 3 months]) were performed within this RR sensitivity analysis, and were similarly applied to the HVPG analysis. As an insufficient number of observational studies reported short‐term follow‐up data to permit a methodologically robust and statistically reliable comparison, this subgroup analysis by follow‐up duration was performed only for the RCTs. Second, to address potential heterogeneity arising from variable background therapies, a post hoc sensitivity analysis was conducted for the outcome of HVPG. This analysis was restricted to RCTs in which all patients in both study arms received non‐selective beta‐blockers (NSBBs), thereby isolating the specific additive hemodynamic effect of statin therapy. Quantitative heterogeneity was assessed using the *I* ^2^ metric, categorised as minimal (0%–25%), moderate (26%–50%), substantial (51%–75%), or considerable (> 75%). Bias due to small‐study effects was assessed by visual inspection of funnel plots [13] and formally tested using Egger's linear regression test of funnel plot asymmetry [14]. This analysis was restricted to meta‐analyses including 10 or more studies, as the interpretation of plot asymmetry is considered unreliable with fewer studies due to low statistical power [15, 16]. Statistical significance was set at 2‐sided *p* < 0.05. Statistical analyses were conducted using R version 4.4.3 (R Project for Statistical Computing), using the meta package [17].

### 2.8 Confidence in the Cumulative Evidence

The Grading of Recommendations Assessment, Development and Evaluation (GRADE) [18] approach was used to evaluate the overall certainty of evidence regarding whether statin administration affects each assessed outcome compared to its non‐use in patients with cirrhosis.

## 3 Results

The PRISMA flowchart of search strategy and included studies is presented in eFigure 1 in Data S1. A total of 3246 articles were identified through the databases and references. After removing duplicates (*n* = 749) and excluding articles that did not meet eligibility criteria according to their titles and abstracts (*n* = 2460), 37 studies were assessed for full‐text eligibility. Following this assessment, 25 studies were selected for inclusion in the systematic review and meta‐analysis. Excluded reports and reasons for exclusion are available in eTable 2 in Data S1.

### 3.1 Study Characteristics

Nine RCTs including a total of 813 cirrhotic patients (396 patients on statins, 417 patients as control) and 16 observational studies including a total of 81,179 cirrhotic patients (25,551 patients on statins, 55,628 patients as control) were included in this systematic review and meta‐analysis. Among the 16 observational studies, 14 were retrospective cohort studies [19, 20, 21, 22, 23, 24, 25, 26, 27, 28, 29, 30, 31, 32] and 2 were case–control studies [33, 34]. Table 1 summarises the characteristics of the included studies, which were all published in peer‐reviewed journals. Further details regarding reported outcomes and statin administration are available in eTable 3 in Data S1. eTable 4 in Data S1 provides the full list of covariates that were adjusted for in the multivariable analyses (adjusted HRs) of the observational studies.

**TABLE 1:** Characteristics of included studies.

Author (year)	Country	Follow‐up	Total (*n*)	Main cirrhosis aetiology	Male sex (%)	MELD/MELDNa score	Use of NSBBs	Child‐Pugh score
						Mean/median		
RCT								
Abraldes 2009 (Multicentre)	Spain	30 days	SU = 28 NSU = 27	SU → Alcohol = 39.3% HCV = 50% HBV = 0% NSU → Alcohol = 44.4% HCV = 48.1%; HBV = 7.4%	SU = 60.7% NSU = 77.8%	SU = 6.2 ± 1.3 NSU = 6.9 ± 1.9	SU = 46.4% NSU = 51.8%	SU → *A* = 64.2% *B* = 35.7% *C* = 0% NSU → *A* = 59.2% *B* = 29.6% *C* = 11.1%
Pollo‐Flores 2015 (Single‐centre)	Brazil	3 months	SU = 14 NSU = 20	Alcohol = 17% HCV = 58% HBV = 17% Autoimmune hepatitis = 8% Not presented separately by SU/NSU. These etiologies were equally distributed between the two groups.	SU = 57% NSU = 50%	SU = 10 (1.5) NSU = 10.5 (7)	SU = 57% NSU = 70%	SU → *A* = 57.1% *B* = 35.7% *C* = 7.2% NSU → *A* = 70% *B* = 25% *C* = 5%
Abraldes 2016 (Multicentre)	Spain	Days−median (IQR) SU = 371 (188–656) NSU = 382 (174–561)	SU = 69 NSU = 78	SU → Alcohol = 71.0% HCV = 27.5% HBV = 1.4% NASH = 1.4% Primary biliary cirrhosis = 4.3% NSU → Alcohol = 71.4% HCV = 22.1% HBV = 2.6% NASH = 5.2% Primary biliary cirrhosis = 0%	SU = 65.2% NSU = 67.9%	SU = 10.15 (4.40) NSU = 10.03 (5.32)	Propranolol or nadolol as a standardised intervention for all participants. SU = 100% NSU = 100%	SU → *A* = 15% *B* = 68% *C* = 17% NSU → *A* = 24% *B* = 62% *C* = 14%
Bishnu 2018 (Single‐centre)	India	1st = 30 days 2nd = 1 year	SU = 11 NSU = 12	SU → Alcohol = 36% HCV = 0% HBV = 0% NASH = 0% Autoimmune hepatitis = 9.1% Wilson's disease = 0% Cryptogenic = 54.55% NSU → Alcohol = 50% HCV = 0% HBV = 8.3% NASH = 8.3% Autoimmune hepatitis = 0% Wilson's disease = 8.3% Cryptogenic = 25%	SU = 81.8% NSU = 100%	SU = 11 (7–14) NSU = 11 (8–16)	Propranolol as a standardised intervention for all participants. SU = 100% NSU = 100%	NAR
Elwan 2018 (Single‐centre)	Egypt	30 days	SU = 20 NSU = 20	NAR	SU = 50% NSU = 80%	NAR	NAR	SU → *A* = 15% *B* = 60% *C* = 25% NSU → *A* = 5% *B* = 45% *C* = 50%
Jha 2019 (Single‐centre)	India	Weeks−mean (SD) 49.05 (25.74)	SU = 65 NSU = 69	SU → Alcohol = 26.2% HCV = 9.2% HBV = 29.2% Cryptogenic = 27.7% NSU → Alcohol = 36.2% HCV = 4.3% HBV = 33.3% Cryptogenic = 20.3%	SU = 69.2% NSU = 73.9%	SU = 15.03 ± 6.46 NSU = 16.49 ± 5.75	Carvedilol as a standardised intervention for all participants. SU = 100% NSU = 100%	SU → *A* = 9.2% *B* = 70.8% *C* = 20% NSU → *A* = 13% *B* = 62.4% *C* = 24.6%
Vijayaraghavan 2020 (Single‐centre)	India	3 months	SU = 110 NSU = 110	SU → Alcohol = 40% HCV = 5.5% HBV = 6.4% NASH = 43.6% NSU → Alcohol = 35.5% HCV = 11.8% HBV = 9.1% NASH = 38.2%	SU = 96% NSU = 86%	SU = 14.4 ± 3.96 NSU = 13.9 ± 4.0	Carvedilol as a standardised intervention for all participants. SU = 100% NSU = 100%	SU → *A* = 33.6% *B* = 50.9% *C* = 15.5% NSU → *A* = 43.6% *B* = 38.2% *C* = 18.2%
Kronborg 2023 (Multicentre)	Denmark	6 months	SU = 38 NSU = 40	SU → Alcohol = 78.9% HBV = 0% NAFLD = 7.9% Autoimmune hepatitis = 0% Primary biliary cirrhosis = 5.3% Methotrexate‐induced = 2.6% NSU → Alcohol = 85% HBV = 2.5% NAFLD = 2.5% Autoimmune hepatitis = 2.5% Primary biliary cirrhosis = 2.5% Methotrexate‐induced = 2.5%	SU = 55.3% NSU = 60.0%	SU = 12.5 [7; 21] NSU = 10.0 [6; 21]	SU = 13.2% NSU = 32.5%	SU → *A* = 31.6% *B* = 55.2% *C* = 13.2% NSU → *A* = 45% *B* = 50% *C* = 5%
Alvarado‐Tapias 2025 (Single‐centre)	Spain	Days−median (IQR) SU = 56 (35–65) NSU = 49 (42–67)	SU = 41 NSU = 41	SU → Alcohol = 39% HCV = 34% Alcohol + HCV = 4% MASLD = 12% NSU → Alcohol = 44% HCV = 34% Alcohol + HCV = 12% MASLD = 5%	SU = 64% NSU = 71%	SU = 9.7 ± 2.7 NSU = 10.3 ± 3.4	SU = 100% NSU = 100%	SU → *A* = 54% *B*–*C* = 46% NSU → *A* = 61% *B*–*C* = 39%
Observational studies								
Kumar 2014 (retrospective cohort)	USA	Months—median (range) SU = 36 (4–385) NSU = 30 (4–220)	SU = 81 NSU = 162	SU → Alcohol = 22% HCV = 22% HBV = 2% NASH = 43% Autoimmune = 4% Cardiac = 5% Other = 10% NSU → Alcohol = 24% HCV = 34% HBV = 6% NASH = 25% Autoimmune = 4% Cardiac = 4% Other = 10%^a^	SU = 54.32% NSU = 54.32%	SU = 11.0 ± 4.0 NSU = 11.7 ± 4.6	SU = 67% NSU = 74%	SU → *A* = 70% *B*–*C* = 30% NSU → *A* = 70% *B*–*C* = 30%
Mohanty 2016 (retrospective cohort)	USA	Years−median Mortality and HCC^b^ SU = 2.4 NSU = 1.9 Decompensation, Ascites, SBP, Variceal Bleeding: SU = 2.3 NSU = 1.7	SU (psm) = 685 NSU (psm) = 2062	SU (psm) → HCV = 100% NSU (psm) → HCV = 100%	SU (psm) = 98.8% NSU = 97.9%	NAR	NAR	NAR
Chang 2017 (case–control)	Taiwan	Years−mean (SD) SU = 5.5 (3.5) NSU = 5.4 (3.6)	SU = 675 NSU = 675	SU → HCV = 23% HBV = 46% Alcohol = 32% NSU → HCV = 23% HBV = 43% Alcohol = 34%	SU = 73% NSU = 71%	NAR	SU = 79% NSU = 79%	NAR
Bang 2017 (retrospective cohort)	Denmark	Years−median (95% CI) Mortality: SU = 7.5 (6.6–8.9) NSU = 5.9 (4.9–6.5) Decompensation: SU = 5.5 (3.4–12.8) NSU = 1.1 (0.7–1.8)	SU (psm) = 248 NSU (psm) = 496	SU (psm) → Alcohol = 100% NSU (psm) → Alcohol = 100%	SU (psm) = 61% NSU (psm) = 54%	NAR	SU (psm) = 30% NSU (psm) = 29%	NAR
Corey 2017 (case–control)	USA	NAR	SU = 55 NSU = 189	SU → NASH = 100% NSU → NASH = 100%	NAR	SU = 11.6 ± 4.4 NSU = 12.39 ± 4.8	NAR	NAR
Patel 2018 (retrospective cohort)	USA	6 months	SU = 19 NSU = 48	SU → HCV = 42.1% Alcohol = 5.3% NASH = 42.1% NSU → HCV = 53.8% Alcohol = 12.3% NASH = 30.8%	NAR	SU = 17 ± 10 NSU = 16 ± 8	NAR	NAR
Kaplan 2019 (retrospective cohort)	USA	Days−median (IQR) SU = 2080 (1268–2872) NSU = 1970 (1234–2736)	SU (psm) = 6481 NSU (psm) = 12,860	SU → HCV + Alcohol = 25.3% HCV = 14.4% Alcohol = 36% NASH = 16.1% NSU → HCV + Alcohol = 26.3% HCV = 14.9% Alcohol = 35.2% NASH = 15.6%	SU (psm) = 98% NSU (psm) = 98%	SU = 8 (6–12) NSU = 9 (6–12)	NAR	SU → *A* = 85.3% *B* = 14.2% *C* = 0.4% NSU → *A* = 83.2% *B* = 16% *C* = 0.7%
Hung 2019 (retrospective cohort)	Taiwan	30 days	SU = 816 NSU = 3264	SU → Alcohol = 8.5% HCV = 12.1% HBV = 13.8% NSU → Alcohol = 8.7% HCV = 11.3% HBV = 13.4%	SU = 57.2% NSU = 59.7%	NAR	NAR	NAR
Goh 2020 (retrospective cohort)	South Korea	Years–median (IQR) 7.2 (0.5–9.9)	SU = 100 NSU = 1754	SU → HBV = 100% NSU → HBV = 100%	NAR	NAR	NAR	NAR
Merkel 2021 (retrospective cohort)	Germany	NOAP	SU = 154(psm) NSU = 154(psm)	SU (psm) → Alcoholism 69 (44.8%) HBV 3 (1.9%) HCV 12 (7.8%) Metabolic 22 (14.3%) Cryptogenic 29 (18.8%) NSU (psm) → Alcoholism 75 (48.7%) Hepatitis *B* 4 (2.6%) Hepatitis *C* 13 (8.4%) Metabolic 12 (7.8%) Cryptogenic 29 26 (16.9%)	SU (psm) = 74.7% NSU (psm) = 79.5%	11.9 ± 4.8 (entire population)	SU (psm) = 57.1% NSU (psm) = 61.7%	SU (psm) = 7.0 ± 2.0 NSU (psm) = 7.3 ± 2.2
Pinyopornpanish 2021 (a) (retrospective cohort)^c^	USA	NOAP	SU = 13,060 NSU = 12,050	NAR	NAR	NAR	NAR	NAR
Pinyopornpanish 2021 (b) (retrospective cohort)^c^	USA	Years−mean (SD) SU = 4.7 (3.1) NSU = 4.5 (3.4)	SU = 392 NSU = 558	NAR	SU = 42.9% NSU = 40.9%	SU = 8.7 ± 3.5 NSU = 9.8 ± 4.2	NA	SU → *A* = 319 (81.4%) *B* = 72 (18.4%) *C* = 1 (0.3%) NSU → *A* = 375 (67.2%) *B* = 161 (28.8%) *C* = 22 (3.9%)
Kraglund 2023 (retrospective cohort)	Denmark	5 years	SU = 1438 trial entries (1351 distinct patients) NSU = 118,460 trial entries (14,653 distinct patients).	Alcohol 100%	SU = 67.1% NSU = 64.8%	NAR	NAR	NAR
Pfisterer 2024 (1) (retrospective cohort)^d^	Austria	Months−median (IQR) 26.8 (37.9)	SU = 32 NSU = 337	SU → Alcohol 14 (43.8%) Viral hepatitis 5 (15.6%) Mixed 0 (0) Cryptogenic 10 (31.3%) NSU → Alcohol 169 (50.1%) Viral hepatitis 71 (21.1%) Mixed 15 (4.4%) Cryptogenic 41 (12.2%)	SU = 59.4% NSU = 66.8%	SU = 10 (5) NSU = 12 (6)	SU = 68.8% NSU = 68.6%	SU → *A* 12 (37.8%) *B* 7 (21.9%) *C* 4 (12.5%) NSU → *A* 93 (27.6%) *B* 111 (32.9%) *C* 60 (17.8%)
Pfisterer 2024 (2) (retrospective cohort)^d^	Austria	Months−Median (IQR) 29.2 (56.7)	SU = 17 NSU = 394	SU → Alcohol 9 (52.9%) Viral hepatitis 1 (5.9%) Mixed 0 (0) Cryptogenic 3 (17.6%) NSU → Alcohol 224 (56.9%) Viral hepatitis 81 (20.6%) Mixed 27 (6.9%) Cryptogenic 38 (9.6%)	SU = 47.1% NSU = 70.1%	SU = 14 (6) NSU = 13 (7)	SU = 82.4% NSU = 69%	SU → *A* 5 (29.4%) *B* 6 (35.3%) *C* 1 (5.9%) NSU → *A* 92 (23.4) *B* 180 (45.7) *C* 89 (22.6)
Cooper 2025 (retrospective cohort)	USA	Years−median (IQR) 1.3 (0.3–2.7)	SU = 127 NSU = 496	SU → Alcohol: 38 (29.9%) MASH: 69 (54.3%) HCV: 9 (7.1%) Autoimmune: 3 (2.4%) Cholestatic: 0 (0.0%) Genetic: 2 (1.6%) NSU → Alcohol: 269 (54.3%) MASH: 78 (15.8%) HCV: 79 (16.0%) Autoimmune: 23 (4.6%) Cholestatic: 15 (3.0%) Genetic: 12 (2.4%)	SU = 71.7% NSU = 61.5%	SU = 11 NSU = 16	NA	NA
Choi 2025 (retrospective cohort)	USA	Years−median (IQR) SU = 6 (2.0–7.9) NSU = 2.8 (1.0–6.3)	SU = 1258 NSU = 5476	NAR	NAR	NAR	NAR	NAR

Abbreviations: NAR, not adequately reported; NOAP, not applicable; NSU, non‐statin user; PSM, propensity score match; SU, statin user.

^a^ Etiologies of cirrhosis add up to greater than 100% because some subjects had more than one disease.

^b^ A specific median follow‐up for HCC was not stated; the value for mortality is the best estimate.

^c^ (a) and (b) are used to distinguish between different studies from the same first author and publication year.

^d^ (1) and (2) are used to distinguish between two separate study populations reported in a single publication, corresponding to patients who underwent primary and secondary prophylaxis of variceal bleeding, respectively.

### 3.2 Risk of Bias

Risk of bias assessments are presented in eFigure 2 in Data S1. We conducted 80 individual outcome‐level risk of bias assessments across the 25 included studies, with each assessment corresponding to a specific outcome reported by a specific study. Of these 80 assessments, 21 were judged to be at low risk of bias, derived from four RCTs [35, 36, 37, 38] and two observational studies [25, 32]. A further 40 assessments were judged to be at moderate risk of bias, originating from four RCTs [39, 40, 41, 42] and five observational studies [20, 26, 28, 33, 34]. Eighteen assessments were judged to be at serious risk of bias, contributed by eight observational studies [19, 21, 22, 23, 24, 27, 30, 31] and one RCT [43]. The remaining assessment was judged to be at critical risk of bias, derived from one observational study [29].

### 3.3 Primary Outcome—Effect of Statins on All‐Cause Mortality

**FIGURE 1:**
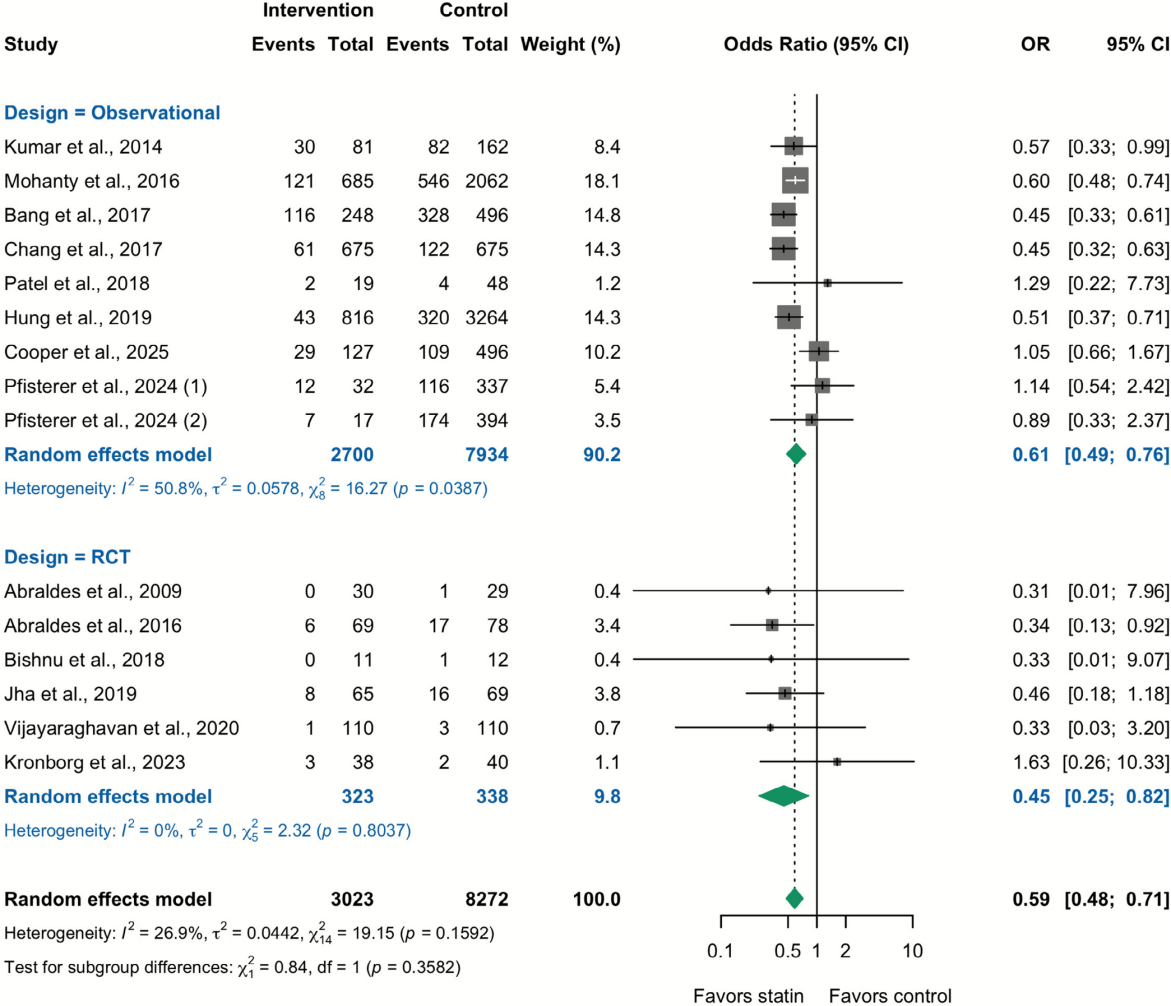
Effect of statins on all‐cause mortality: Meta‐analysis of unadjusted odds ratios from RCTs and observational studies. The squares represent point estimates, and the size of each square is proportional to the weight of the study. Horizontal lines indicate the 95% CI of the unadjusted odds ratio (OR) estimate in each study. The diamond represents the pooled point estimate, and its width represents the pooled estimate 95% CI. Summary effects are presented separately for the RCTs and observational studies, as well as for the overall combined analysis.

**FIGURE 2:**
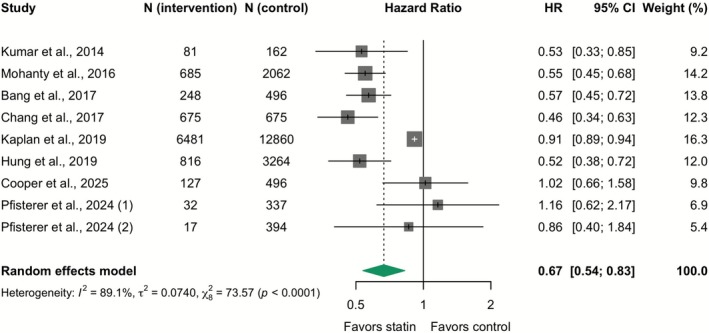
Effect of statins on all‐cause mortality: Meta‐analysis of adjusted hazard ratios from observational studies. The squares represent point estimates, and the size of each square is proportional to the weight of the study. Horizontal lines indicate the 95% CI of the adjusted hazard ratio (HR) estimate in each study. The diamond represents the pooled point estimate, and its width represents the pooled estimate 95% CI.

Fourteen studies (11,295 patients) reported data on mortality. In the overall analysis combining RCTs and observational studies, statin therapy was associated with lower odds of death (Unadjusted OR 0.59; 95% CI 0.48–0.71; *I* ^2^ = 26.9%). This association was consistent across study designs: in RCTs (6 trials), statins reduced mortality (OR 0.45; 95% CI 0.25–0.82; *I* ^2^ = 0%), and in observational studies (8 studies), statins likewise reduced mortality (Unadjusted OR 0.61; 95% CI 0.49–0.76; *I* ^2^ = 50.8%) (Figure 1). In observational studies, the pooled adjusted HR—considered the preferred estimate because it accounts for confounding—also demonstrated a significant reduction in mortality (adjusted HR 0.67; 95% CI 0.54–0.83; *I* ^2^ = 89.1%), albeit with considerable heterogeneity (Figure 2). In a sensitivity analysis using RR to aid clinical interpretability, the benefit in RCTs remained consistent (RR 0.50; 95% CI 0.30–0.85; *I* ^2^ = 0%), and was confined to long‐term follow‐up (eFigure 4 in Data S1). There was no evidence of publication bias according to Egger's test in the overall analysis (*p* = 0.49) (eFigure 6 in Data S1).

### 3.4 Primary Outcome—Effect of Statins on Hepatic Decompensation

**FIGURE 3:**
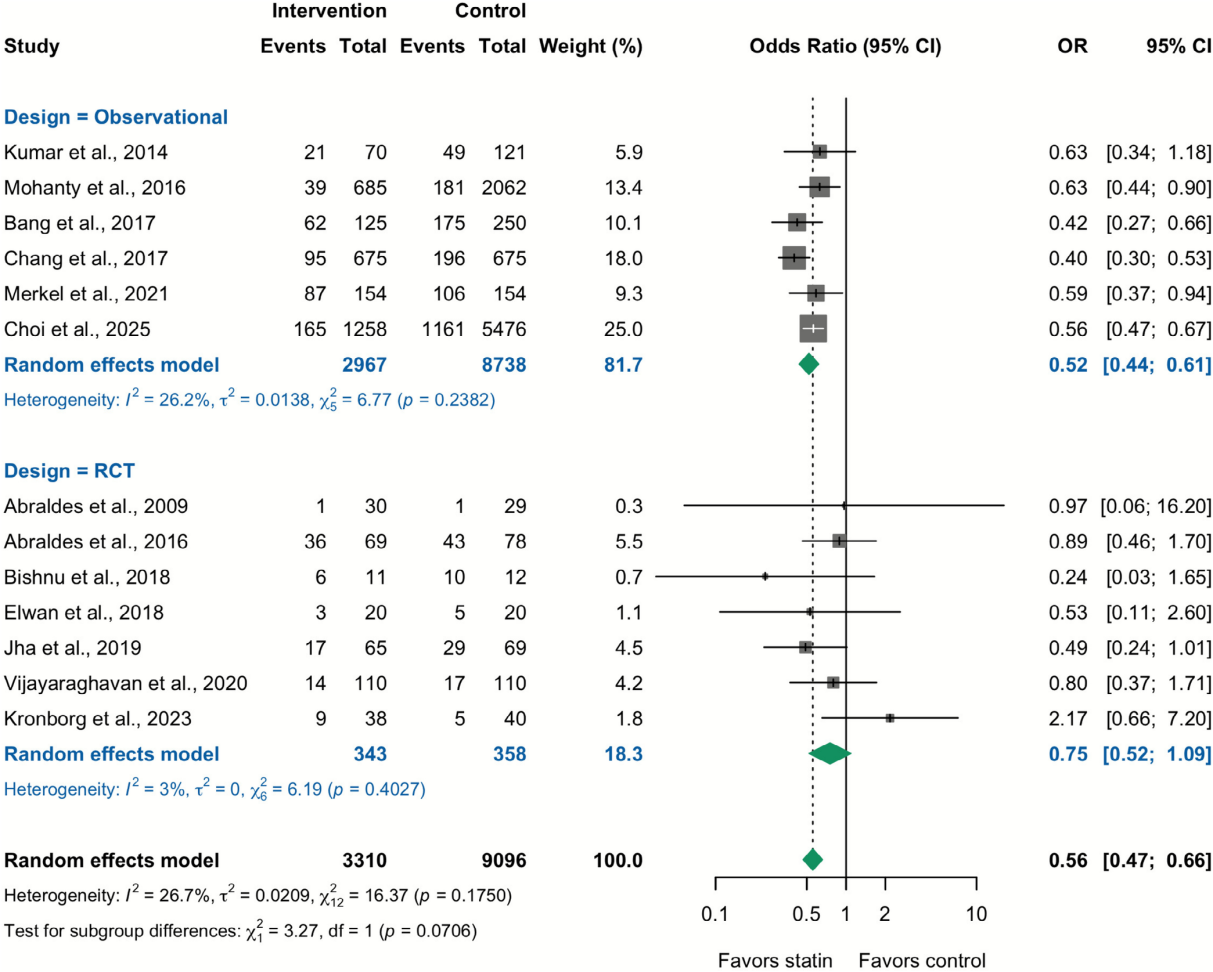
Effect of statins on hepatic decompensation: Meta‐analysis of unadjusted odds ratios from RCTs and observational studies. The squares represent point estimates, and the size of each square is proportional to the weight of the study. Horizontal lines indicate the 95% CI of the unadjusted odds ratio (OR) estimate in each study. The diamond represents the pooled point estimate, and its width represents the pooled estimate 95% CI. Summary effects are presented separately for the RCTs and observational studies, as well as for the overall combined analysis.

**FIGURE 4:**
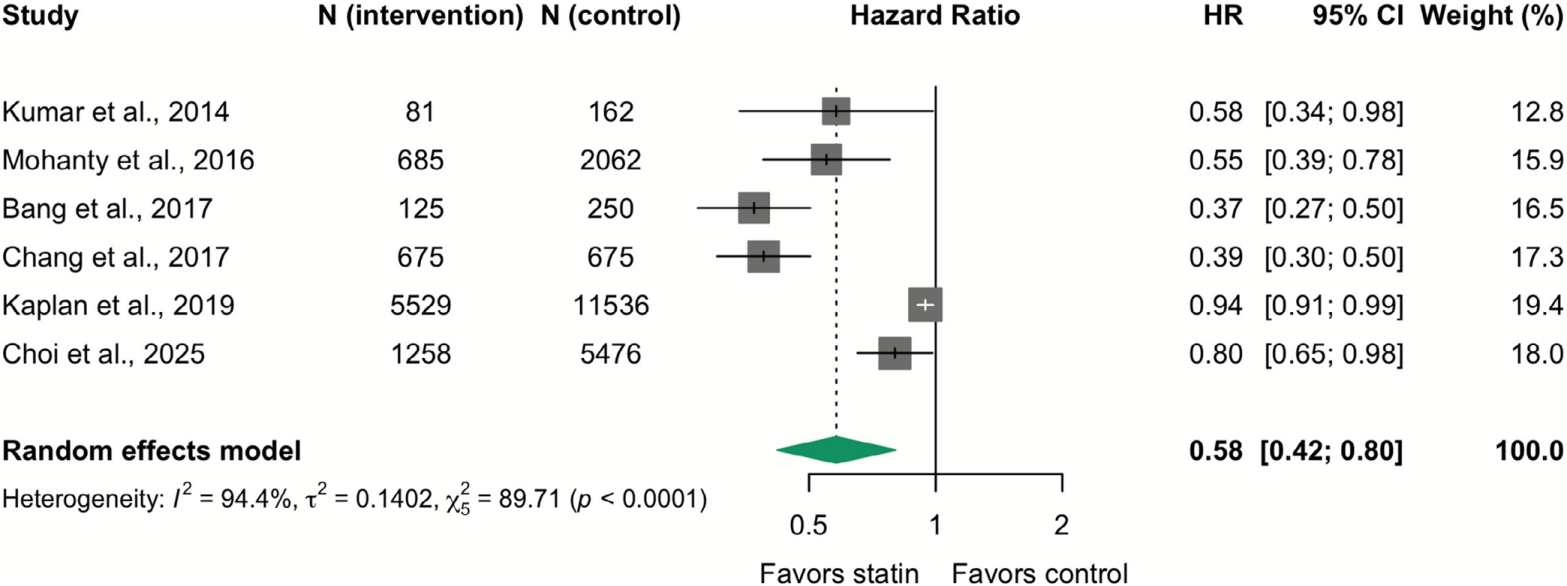
Effect of statins on hepatic decompensation: Meta‐analysis of adjusted hazard ratios from observational studies. The squares represent point estimates, and the size of each square is proportional to the weight of the study. Horizontal lines indicate the 95% CI of the adjusted hazard ratio (HR) estimate in each study. The diamond represents the pooled point estimate, and its width represents the pooled estimate 95% CI.

Thirteen studies (12,406 patients) reported data on hepatic decompensation. In the overall analysis combining RCTs and observational studies, statin therapy was associated with lower odds of decompensation (Unadjusted OR 0.56; 95% CI: 0.47–0.66; *I* ^2^ = 26.7%). However, this association differed by study design: in RCTs (7 trials), statins did not significantly reduce the risk of decompensation (OR 0.75; 95% CI: 0.52–1.09; *I* ^2^ = 3.0%), while in observational studies (6 studies), statins reduced the risk of decompensation (Unadjusted OR 0.52; 95% CI: 0.44–0.61; *I* ^2^ = 26.2%) (Figure 3). The pooled adjusted HR for observational studies, again considered the preferred estimate, also demonstrated a significant reduction in decompensation (Adjusted HR 0.58; 95% CI: 0.42–0.80; *I* ^2^ = 94.4%), although with considerable heterogeneity (Figure 4). In a sensitivity analysis using RR to aid clinical interpretability, the finding for RCTs was unchanged (RR 0.83; 95% CI: 0.66–1.05; *I* ^2^ = 0.0%), and showed no significant effect in either long‐term or short‐term follow‐up (eFigure 4 in Data S1). There was no evidence of publication bias according to Egger's test in the overall analysis (*p* = 0.31) (eFigure 6 in Data S1).

### 3.5 Primary Outcome—Effect of Statins on HCC

**FIGURE 5:**
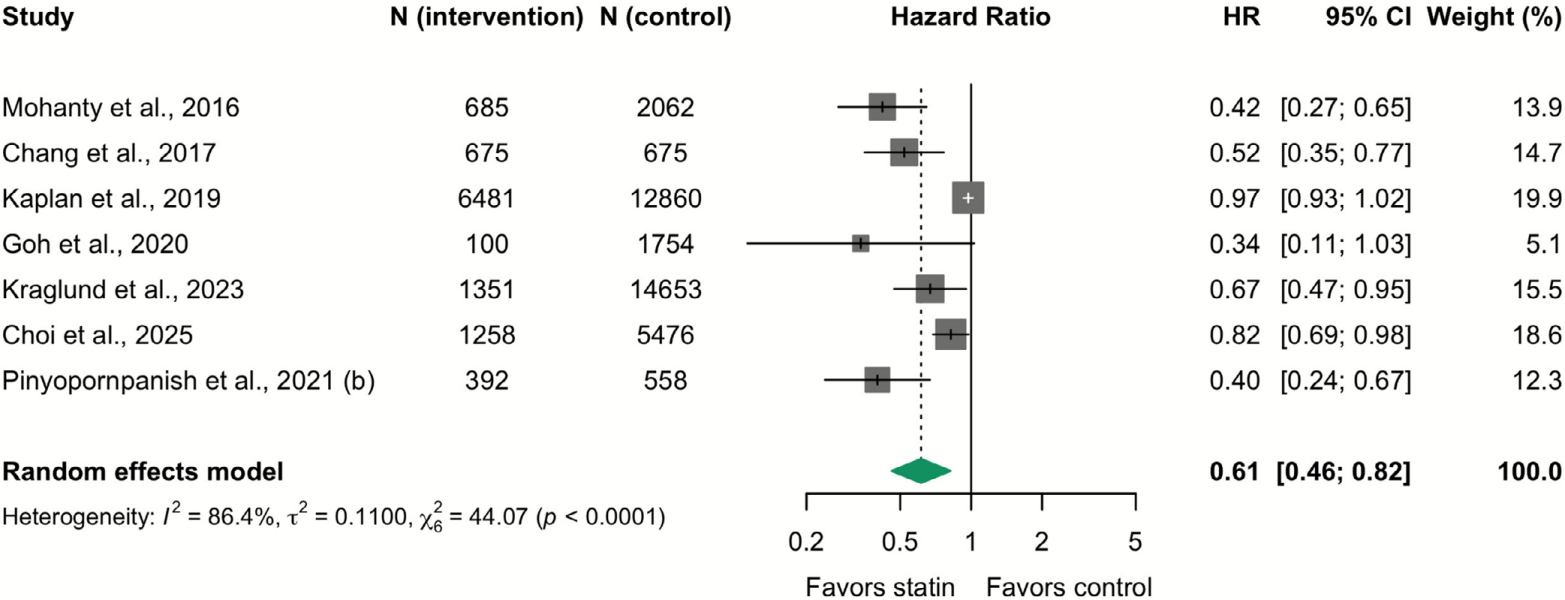
Effect of statins on hepatocellular carcinoma: Meta‐analysis of adjusted hazard ratios from observational studies. The squares represent point estimates, and the size of each square is proportional to the weight of the study. Horizontal lines indicate the 95% CI of the adjusted hazard ratio (HR) estimate in each study. The diamond represents the pooled point estimate, and its width represents the pooled estimate 95% CI.

In a meta‐analysis of eight observational studies (39,297 patients), statin therapy was associated with lower odds of HCC (Unadjusted OR 0.65; 95% CI: 0.52–0.81; *I* ^2^ = 64.6%), although with substantial heterogeneity (eFigure 3 in Data S1). Pooled analysis of adjusted HRs from seven observational studies (48,980 patients), again considered the preferred estimate, also demonstrated a significant reduction in HCC (Adjusted HR 0.61; 95% CI: 0.46–0.82; *I* ^2^ = 86.4%), although with considerable heterogeneity (Figure 5). HCC data were derived exclusively from observational studies because no RCTs assessed this outcome; accordingly, an RCT analysis was precluded.

### 3.6 Secondary Outcomes—Effect of Statins on Individual Decompensation Events

In the overall pooled analyses combining both RCTs and observational studies, statin use was associated with significantly lower odds of variceal bleeding (Unadjusted OR 0.62; 95% CI: 0.43–0.90; *I* ^2^ = 32.2%) and ascites (Unadjusted OR 0.74; 95% CI: 0.57–0.96; *I* ^2^ = 0.0%). In contrast, the analyses limited to RCTs did not show a significant effect for either ascites or variceal bleeding. Regarding HRS, SBP, and HE, no significant associations were found in the overall analyses. However, the analysis limited to RCTs demonstrated a significant reduction in the odds of SBP (OR 0.24; 95% CI: 0.06–0.98; *I* ^2^ = 0.0%). These analyses are presented in eFigure 3 (Data S1). In sensitivity analyses using RR to aid clinical interpretability, findings were largely consistent; notably, the reduction in SBP observed in RCTs maintained a similar effect size but reached borderline statistical significance (RR 0.25; 95% CI: 0.06–1.00; *I* ^2^ = 0.0%) (eFigure 4 in Data S1).

### 3.7 Secondary Outcome—Effect of Statins on Change in HVPG

**FIGURE 6:**
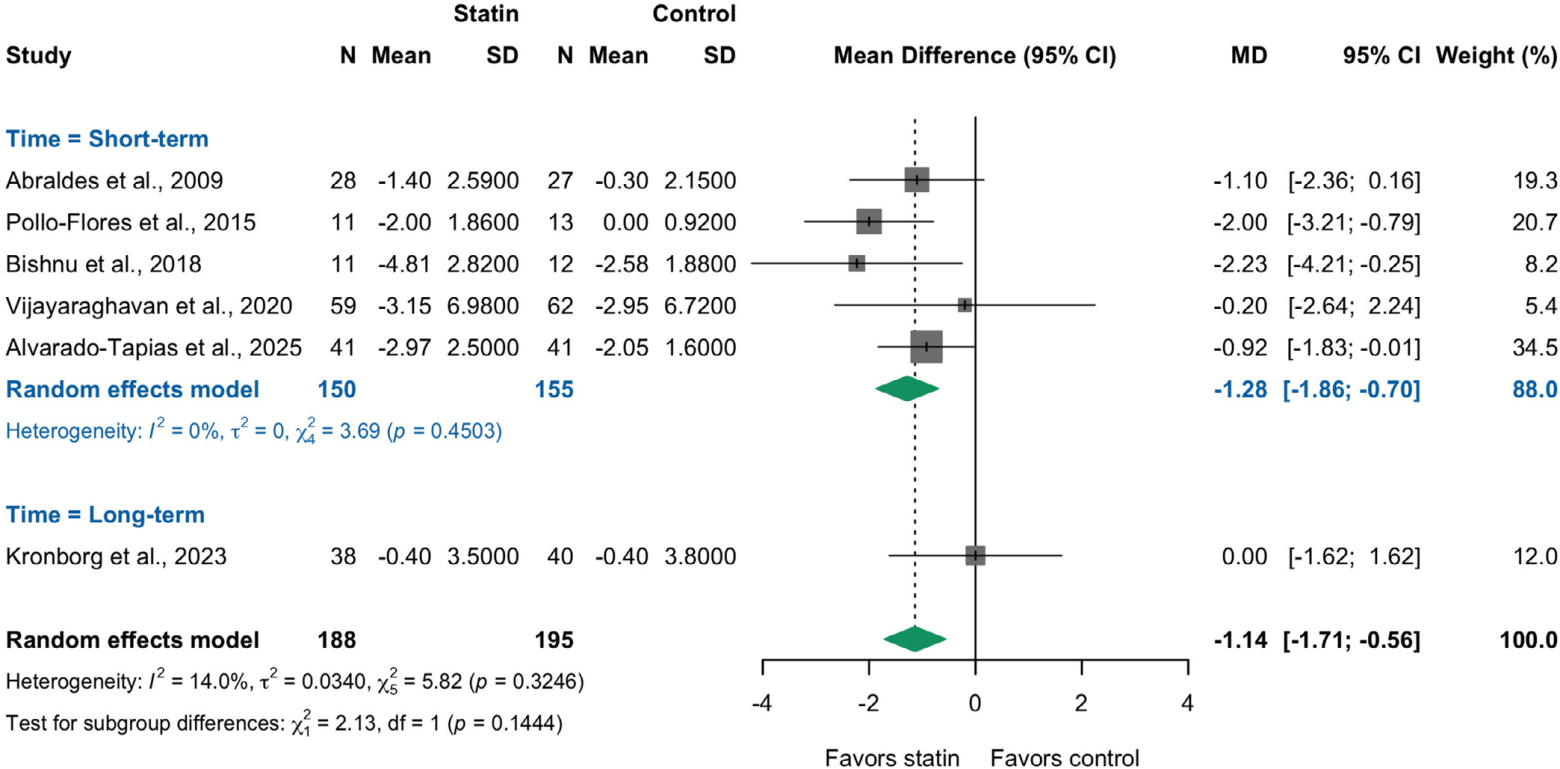
Effect of statins on change in HVPG: Meta‐analysis of mean difference from RCTs. The squares represent point estimates, and the size of each square is proportional to the weight of the study. Horizontal lines indicate the 95% CI of the mean difference (MD) estimate in each study. The diamond represents the pooled point estimate, and its width represents the pooled estimate 95% CI. Summary effects are presented for the short‐term follow‐up subgroup and for the overall combined analysis. As only one long‐term follow‐up study was included, its individual estimate is shown without a separate subgroup summary.

In a meta‐analysis of six RCTs (383 patients), statin therapy was associated with a significant reduction in HVPG (MD −1.14 mmHg; 95% CI: −1.71 to −0.56; *I* ^2^ = 14.0%). This effect was consistent in the short‐term follow‐up subgroup (MD −1.28 mmHg; 95% CI: −1.86 to −0.70; *I* ^2^ = 0.0%), and a pooled analysis for the long‐term subgroup was not feasible as only one trial was available (Figure 6). In a post hoc sensitivity analysis restricted to the three RCTs [37, 38, 40] in which all patients in both study arms received NSBBs, statin therapy was also associated with a significant reduction in HVPG (MD −1.05 mmHg; 95% CI: −1.83 to −0.27; *I* ^2^ = 0%). This result reflects the short‐term follow‐up, as long‐term hemodynamic data were not reported for any of these trials (eFigure 5 in Data S1).

### 3.8 Certainty of Evidence

The certainty of the evidence for each outcome, assessed via the GRADE approach, is presented in eTable 5 in Data S1.

## 4 Discussion

In this updated and comprehensive meta‐analysis of 25 studies (9 randomised controlled trials and 16 observational studies), including a total of 81,992 patients with cirrhosis, four main findings were identified: statin therapy was associated with (1) a robust reduction in all‐cause mortality; (2) a reduction in hepatic decompensation, a finding driven by observational data; (3) a significant decrease in the HVPG in RCTs; and (4) a lower incidence of HCC in observational studies.

The primary rationale for investigating statins in cirrhosis extends beyond their established lipid‐lowering properties. Statins are well‐known for their pleiotropic effects, which may counteract the pathophysiology of portal hypertension and liver fibrosis [44]. Despite historical concerns regarding hepatotoxicity, a growing body of evidence suggests statins may modulate the increased intrahepatic vascular resistance that drives portal hypertension [45]. Proposed mechanisms include upregulation of endothelial nitric oxide synthase, with increased nitric oxide bioavailability and vasodilation [40, 46]. Furthermore, statins exert direct anti‐inflammatory and anti‐fibrotic actions, partly by inhibiting the activation and contractility of hepatic stellate cells, the main fibrogenic cells in the liver, and by attenuating the expression of profibrogenic cytokines such as TGF‐β1 [41, 47]. Collectively, these multifaceted mechanisms provide a strong biological plausibility for the use of statins to slow disease progression, prevent hepatic decompensation, and potentially reduce the risk of hepatocarcinogenesis.

This meta‐analysis establishes a consistent association between statin therapy and lower all‐cause mortality. This benefit was evident in the overall pooled analysis, in the observational studies and, critically, in the pooled analysis of RCTs, in which the effect was driven primarily by trials with long‐term follow‐up, supporting a potential causal relationship. This is important, as the cardiovascular benefits of statins typically emerge over extended treatment periods and tend to increase with duration [48, 49]. Although the pooled adjusted hazard ratio from observational studies showed considerable heterogeneity, the consistent association with reduced mortality across the study designs is encouraging. These findings are promising but warrant confirmation in larger, well‐designed trials.

A key mechanistic finding of our analysis is the significant reduction in HVPG observed in the pooled RCT data. This clinically meaningful decrease was consistent across short‐term trials with low heterogeneity, providing robust evidence of a direct, beneficial hemodynamic effect. Crucially, to address the potential confounding effect of background therapy, we performed a post hoc sensitivity analysis restricted to RCTs in which statins were administered as an add‐on therapy to standard NSBBs in both arms. In this analysis, statin therapy maintained a significant reduction in HVPG, indicating that its hemodynamic benefits are additive to those of NSBBs. As the primary surrogate marker for portal hypertension, this improvement suggests that statins exert pleiotropic actions within the hepatic microcirculation, including the enhancement of endothelial function. Therefore, this short‐term hemodynamic benefit provides a strong mechanistic rationale for the long‐term clinical outcomes observed in our analysis, bridging the gap between biological plausibility and the reduction in mortality and decompensation.

In contrast to mortality, the effect of statins on hepatic decompensation differed by study design: RCTs showed no significant benefit, whereas observational studies suggested a protective association. In these observational studies, the association was consistent across both unadjusted odds ratio and adjusted hazard ratio analyses and was particularly evident for ascites. The discordance in the decompensation findings is best explained by the difference in follow‐up duration by study design. The shorter duration of RCTs was plausibly insufficient to capture a statistically significant difference in major clinical outcomes that might take years to develop, like decompensation, even while successfully showing a mechanistic benefit (HVPG reduction). In contrast, the multi‐year timeframe of observational studies provided a large enough window to observe the cumulative, real‐world benefits of a chronic therapy. Their positive findings suggest that the early, mechanistic benefits of statins seen in RCTs do, over time, translate into a reduced risk of decompensation. Therefore, the findings are likely complementary, not contradictory. The RCTs demonstrate a short‐term biological effect, while observational studies suggest its eventual translation into a long‐term clinical outcome, underscoring the need for future trials with extended follow‐up.

In the overall pooled analyses combining both RCTs and observational studies, statin use was associated with significantly lower odds of variceal bleeding, which was not confirmed in RCT only analysis, despite consistent hemodynamic effects. This apparent discrepancy is likely multifactorial. The HVPG trials enrolled relatively small numbers of patients, with few bleeding events and predominantly short‐term follow‐up, limiting statistical power to detect differences in clinical outcomes. In addition, the average HVPG reduction of approximately 1–1.5 mmHg, while mechanistically indicating that statins target intrahepatic resistance, may not be sufficient for many patients to achieve the established threshold reductions associated with meaningful decreases in bleeding risk (HVPG < 12 mmHg or a > 20% reduction from baseline) [50, 51]. Heterogeneity in baseline variceal status, prior bleeding history, and concomitant endoscopic and pharmacological therapies may also have attenuated any potential effect on variceal haemorrhage. Larger, adequately powered RCTs with longer follow‐up and clinically oriented endpoints are required to determine whether the hemodynamic benefits of statins ultimately translate into fewer decompensation events, including variceal bleeding. Conversely, the robust reduction in all‐cause mortality and the decrease in overall decompensation seen in observational data suggest that the major clinical benefits of statins are likely mediated through broader pleiotropic mechanisms—such as improved endothelial function, reduced inflammation, and fibrosis regression—rather than solely through portal pressure lowering.

Furthermore, this meta‐analysis highlights a significant association between statin use and a lower incidence of HCC, an outcome for which evidence was derived exclusively from observational studies. However, these non‐randomised data cannot establish causality and are subject to residual confounding, a concern underscored by substantial heterogeneity in the analysis. Although this potential chemopreventive effect is biologically plausible, as statins can disrupt key signalling pathways that control tumour cell growth and survival [52], it requires definitive confirmation from RCTs.

Prior meta‐analyses and large cohort studies have consistently reported that statin use is associated with lower risks of hepatic decompensation and all‐cause mortality in patients with cirrhosis [5, 6]. In line with this, another meta‐analysis from 2019 also found that long‐term statin therapy reduced mortality, lowered the incidence of HCC, and decreased the risk of specific decompensation events, including ascites and HE [53]. By contrast, a more recent meta‐analysis from 2025 focusing mainly on hemodynamic endpoints found a reduction in HVPG but no significant differences in variceal bleeding, ascites, or mortality [7]. However, it included a smaller evidence base (6 RCTs, no observational studies) and predominantly short‐term follow‐up, which may have reduced the statistical power of the analysis in foreseeing the long‐term effects of statin therapy. Crucially, the chemopreventive potential of statins has also been reinforced by recent large‐scale meta‐analyses examining statin use across broadly defined populations. Wong et al. [54] demonstrated a consistent risk reduction in HCC development among individuals with chronic liver disease, while Zeng et al. [55] reported a similar protective association in analyses encompassing all statin users. Importantly, both studies confirmed a robust and significant reduction in HCC risk specifically within their cirrhosis subgroups. Our work strengthens the current evidence base by integrating newly available studies with both randomised and observational data, yielding a more contemporary and comprehensive evaluation of the effects of statin therapy in this high‐risk population.

Guidelines have increasingly acknowledged the potential of statins to lower portal pressure and improve survival through pleiotropic effects, while noting historical uncertainty around safety in cirrhosis [56]. In that scenario, our analysis offers evidence of its beneficial effect. The Baveno VII guidelines also recommend statin use in patients with cirrhosis who have a clinical indication, citing their potential to decrease portal hypertension and improve overall survival [4]. In line with this evolving perspective, our findings support the growing recognition of statins as more than lipid‐lowering agents, suggesting a potential disease‐modifying role in cirrhosis. Clinically, this evidence reassures practitioners regarding the safety of statin use in patients with compensated cirrhosis and highlights their possible benefit in reducing mortality and decompensation, even beyond cardiovascular prevention. However, current data remain insufficient to recommend statins as standard therapy for all patients with cirrhosis, particularly those with advanced disease. Future research should prioritise adequately powered, multicentre RCTs with longer follow‐up to validate these benefits, clarify the optimal type and dose of statins, and evaluate their role across different etiologies and severities of cirrhosis. Trials linking hemodynamic improvements to long‐term outcomes and safety assessments in decompensated populations will be especially valuable to guide clinical practice and inform future guidelines.

Our study has limitations. First, our conclusions for the long‐term outcomes of decompensation and HCC rely heavily on observational data, which is inherently susceptible to residual confounding and selection bias despite statistical adjustment. Second, substantial heterogeneity was present across the included studies. The analyses pooled data from clinically diverse populations with cirrhosis, spanning multiple etiologies and wide variation in disease severity. Statin exposure also varied considerably, including differences in the specific agent, dosing strategies, and treatment duration. This degree of clinical and pharmacologic variability limits the ability to define an optimal statin regimen or standardised therapeutic protocol based on the available evidence. Critically, considering NSBBs are the cornerstone of therapy for preventing decompensation, their inconsistent reporting, particularly in the included RCTs, introduces a major potential confounder that complicates the interpretation of decompensation outcomes. Several trials evaluated statins as an adjunctive therapy added to a standardised NSBB backbone such as carvedilol [38, 39, 40], propranolol [37] and propranolol or nadolol [35]. In contrast, other trials compared statins to a placebo where NSBBs were a variable co‐intervention, which was handled through stratification [36], was present with baseline imbalances [41, 43], or was inconsistently described [42]. Finally, the existing RCT evidence is itself limited, as the trials were mostly proof‐of‐concept or small single‐centre investigations with limited sample sizes and with shorter follow‐up durations, which precluded the evaluation over the multi‐year time horizons that were available for the observational studies.

In summary, statin therapy in cirrhosis is consistently associated with a reduction in all‐cause mortality, a finding mechanistically supported by reductions in portal pressure in RCTs. Observational studies suggest possible benefits in preventing hepatic decompensation and HCC, but the certainty of this evidence is low and requires confirmation. Current randomised data are limited by sample size and short follow‐up, underscoring the need for adequately powered, multicentre trials with long‐term outcomes. Until such evidence is available, statins should be considered safe in compensated cirrhosis when otherwise indicated, with their potential disease‐modifying effects remaining an exciting opportunity.

## Author Contributions

**Bernardo de Faria Moraes:** conceptualization, data curation, formal analysis, methodology, software, project administration, writing – original draft preparation, writing – review and editing, and final approval of the version to be published. **Gustavo André Pedral Diniz Leite:** data curation, formal analysis, writing – original draft preparation, writing – review and editing, and final approval of the version to be published; guarantor. **Igor Boechat Silveira:** data curation, formal analysis, writing – original draft preparation, writing – review and editing, and final approval of the version to be published. **Gabriel André Pedral Diniz Leite:** data curation, formal analysis, writing – original draft preparation, writing – review and editing, and final approval of the version to be published. **Maria Luisa Motta Fonseca:** data curation, formal analysis, writing – original draft preparation, writing – review and editing, and final approval of the version to be published. **Leonardo Corrêa Suffert:** data curation, formal analysis, writing – original draft preparation, writing – review and editing, and final approval of the version to be published. **Luisa Medeiros Visentini:** data curation, formal analysis, writing – original draft preparation, writing – review and editing, and final approval of the version to be published. **Luis Pedro Possapp Beis:** data curation, formal analysis, writing – original draft preparation, writing – review and editing, and final approval of the version to be published. **Guilherme Grossi Lopes Cançado:** conceptualization, data curation, formal analysis, methodology, project administration, writing – original draft preparation, writing – review and editing, supervision, and final approval of the version to be published; guarantor.

## Funding

The authors have nothing to report.

## Conflicts of Interest

The authors declare no conflicts of interest.

## Supporting information


**Data S1:** apt70526‐sup‐0001‐Supinfo1.docx.

## Data Availability

The data that support the findings of this study are available from the corresponding author upon reasonable request.
