# Supplementary material for: Statin Therapy Independently Reduces Mortality and Liver Complications in Patients With Cirrhosis: An Updated Systematic Review and Meta‐Analysis
Source: Aliment Pharmacol Ther. 2026 Jan 10;63(6):778–94. doi: 10.1111/apt.70526 (PMC12934547; doi:10.1111/apt.70526)
Supplement: Supplementary file 1 — Data S1: apt70526‐sup‐0001‐Supinfo1.docx. [file APT-63-778-s001.docx]

**Supplementary Online Content**

**eTable 1:** Detailed information on search strategies.

**eTable 2:** Excluded reports and reasons for exclusion.

**eTable 3:** Characteristics of included studies: reported outcomes and statin administration.

**eTable 4:** Covariates adjusted for in the multivariable analyses (adjusted hazard ratios) of included studies.

**eTable 5:** GRADE table.

**eText 1:** Detailed definitions of outcomes.

**eFigure 1:** Preferred Reporting Items for Systematic Reviews and Meta-analyses (PRISMA) flowchart of search strategy and included studies.

**eFigure 2:** Risk of bias assessments**.**

**eFigure 3:** Forest Plots.

**eFigure 4:** Sensitivity analysis of RCTs using relative risk.

**eFigure 5:** Post hoc sensitivity analysis of the effect of statins on change in HVPG restricted to RCTs in which all participants received non-selective beta-blockers.

**eFigure 6:** Funnel Plots.

**eTable 1:** Detailed information on search strategies.

| Information Source | Method |
| --- | --- |
| PubMed | ("Hydroxymethylglutaryl-CoA Reductase Inhibitors"[Mesh] OR "Rosuvastatin Calcium"[MeSH] OR "Atorvastatin"[MeSH] OR "Simvastatin"[MeSH] OR "Pravastatin"[MeSH] OR "Fluvastatin"[MeSH] OR "Lovastatin"[MeSH] OR "hydroxymethylglutaryl-CoA reductase inhibitors"[tiab] OR "HMG-CoA reductase inhibitors"[tiab] OR statin[tiab] OR statins[tiab] OR atorvastatin[tiab] OR rosuvastatin[tiab] OR simvastatin[tiab] OR pravastatin[tiab] OR fluvastatin[tiab] OR lovastatin[tiab] OR pitavastatin[tiab]) AND ("Liver Cirrhosis"[Mesh] OR "End Stage Liver Disease"[MeSH] OR cirrhosis[tiab] OR cirrhotic[tiab] OR "liver fibrosis"[tiab] OR "hepatic fibrosis"[tiab] OR "end-stage liver disease"[tiab] OR "advanced chronic liver disease"[tiab]) |
| Embase | ('hydroxymethylglutaryl coa reductase inhibitor'/exp OR 'atorvastatin'/exp OR 'rosuvastatin'/exp OR 'simvastatin'/exp OR 'pravastatin'/exp OR 'fluvastatin'/exp OR 'lovastatin'/exp OR 'pitavastatin'/exp OR 'hydroxymethylglutaryl-coa reductase inhibitor':ti,ab OR 'hmg-coa reductase inhibitor':ti,ab OR 'statin':ti,ab OR 'statins':ti,ab OR 'atorvastatin':ti,ab OR 'rosuvastatin':ti,ab OR 'simvastatin':ti,ab OR 'pravastatin':ti,ab OR 'fluvastatin':ti,ab OR 'lovastatin':ti,ab OR 'pitavastatin':ti,ab) AND ('liver cirrhosis'/exp OR 'liver fibrosis'/exp OR 'end stage liver disease'/exp OR 'cirrhosis':ti,ab OR 'cirrhotic':ti,ab OR 'liver fibrosis':ti,ab OR 'hepatic fibrosis':ti,ab OR 'end-stage liver disease':ti,ab OR 'advanced chronic liver disease':ti,ab) |
| Cochrane Central Register of Controlled Trials (CENTRAL)^a^ | (hydroxymethylglutaryl-CoA reductase inhibitors OR HMG-CoA reductase inhibitors OR statin OR statins OR atorvastatin OR rosuvastatin OR simvastatin OR pravastatin OR fluvastatin OR lovastatin OR pitavastatin) AND (cirrhosis OR cirrhotic OR liver fibrosis OR hepatic fibrosis OR end-stage liver disease OR advanced chronic liver disease) |
| Citation searching | Relevant reviews^1-4^ were screened for their included studies.  The reference lists of included articles were screened to identify additional relevant studies. |

Table legend:

1. The search in CENTRAL was conducted via the Cochrane Library (Wiley). For this database, all terms were searched as free text within the Title, Abstract, and Keywords fields.

References cited in this table:

1. Abdulrazzak E, Fakhoury B, Jaan A, et al. Statin Therapy and Portal Pressure Reduction in Cirrhosis: A Systematic Review and Meta-Analysis. *Liver Int*. 2025;45(10):e70326. doi:10.1111/liv.70326
2. Gu Y, Yang X, Liang H, et al. Comprehensive evaluation of effects and safety of statin on the progression of liver cirrhosis: a systematic review and meta-analysis. *BMC Gastroenterol*. 2019;19(1):231. Published 2019 Dec 30. doi:10.1186/s12876-019-1147-1
3. Kamal S, Khan MA, Seth A, et al. Beneficial Effects of Statins on the Rates of Hepatic Fibrosis, Hepatic Decompensation, and Mortality in Chronic Liver Disease: A Systematic Review and Meta-Analysis. *Am J Gastroenterol*. 2017;112(10):1495-1505. doi:10.1038/ajg.2017.170
4. Kim RG, Loomba R, Prokop LJ, et al. Statin Use and Risk of Cirrhosis and Related Complications in Patients With Chronic Liver Diseases: A Systematic Review and Meta-analysis. *Clin Gastroenterol Hepatol*. 2017;15(10):1521-1530.e8. doi:10.1016/j.cgh.2017.04.039

**eTable 2:** Excluded reports and reasons for exclusion.

| 1 | Blanc JF, Khemissa F, Bronowicki JP, et al. Phase 2 trial comparing sorafenib, pravastatin, their combination or supportive care in HCC with Child-Pugh B cirrhosis. *Hepatol Int*. 2021;15(1):93-104. doi:10.1007/s12072-020-10120-3 | Wrong patient population - All patients had hepatocellular carcinoma (HCC) at baseline. |
| --- | --- | --- |
| 2 | Amjad W, Jiang Z, Lai M. Statin use in cirrhosis and its association with incidence of portal vein thrombosis. *J Gastroenterol Hepatol*. 2024;39(5):955-963. doi:10.1111/jgh.16495 | No outcomes of interest - Outcome assessed was portal vein thrombosis, which was not included in this review. |
| 3 | Jeon D, Cha HR, Chung SW, et al. Association between statin use and the prognosis of hepatocellular carcinoma after resection: a nationwide cohort study. *EClinicalMedicine*. 2023;65:102300. Published 2023 Nov 1. doi:10.1016/j.eclinm.2023.102300 | Wrong patient population - All patients had hepatocellular carcinoma (HCC) at baseline. |
| 4 | Motzkus-Feagans C, Pakyz AL, Ratliff SM, Bajaj JS, Lapane KL. Statin use and infections in Veterans with cirrhosis. *Aliment Pharmacol Ther*. 2013;38(6):611-618. doi:10.1111/apt.12430 | No outcomes of interest - Outcome assessed was time to first serious infection, which was not included in this review. |
| 5 | Han JE, Kim J, Cheong JY, et al. The Impact of Statins on the Survival of Patients with Advanced Hepatocellular Carcinoma Treated with Sorafenib or Lenvatinib. *Cancers (Basel)*. 2024;16(2):249. Published 2024 Jan 5. doi:10.3390/cancers16020249 | Wrong patient population - All patients had hepatocellular carcinoma (HCC) at baseline. |
| 6 | Jeon CY, Goodman MT, Cook-Wiens G, Sundaram V. Statin Use and Survival with Early-Stage Hepatocellular Carcinoma. *Cancer Epidemiol Biomarkers Prev*. 2016;25(4):686-692. doi:10.1158/1055-9965.EPI-15-1040 | Wrong patient population - All patients had hepatocellular carcinoma (HCC) at baseline. |
| 7 | Ramsing MS, Kraglund F, Jepsen P. Prevalence of Statin Use and Predictors of Statin Initiation Among Patients with Alcohol-Related Cirrhosis - A Danish Nationwide Cohort Study. *Clin Epidemiol*. 2023;15:435-446. Published 2023 Apr 1. doi:10.2147/CLEP.S401862 | No outcomes of interest - Outcomes assessed were prevalence of statin use and incidence of statin initiation and discontinuation, which were not included in this review. |
| 8 | Pose E, Jiménez C, Zaccherini G, et al. Simvastatin and Rifaximin in Decompensated Cirrhosis: A Randomized Clinical Trial. *JAMA*. 2025;333(10):864-874. doi:10.1001/jama.2024.27441 | Wrong exposure - Intervention consisted of simvastatin administered in combination with rifaximin, compared with placebo, precluding assessment of the isolated effect of statin therapy. |
| 9 | Muñoz AE, Pollarsky F, Marino M, et al. Safety of Chronic Simvastatin Treatment in Patients with Decompensated Cirrhosis: Many Adverse Events but No Liver Injury. *Dig Dis Sci*. 2021;66(9):3199-3208. doi:10.1007/s10620-020-06630-7 | No outcomes of interest - Outcomes assessed were safety and tolerability endpoints of simvastatin, which were not included in this review. |
| 10 | Zafra C, Abraldes JG, Turnes J, et al. Simvastatin enhances hepatic nitric oxide production and decreases the hepatic vascular tone in patients with cirrhosis. *Gastroenterology*. 2004;126(3):749-755. doi:10.1053/j.gastro.2003.12.007 | No outcomes of interest - Study evaluated the acute hemodynamic effects of simvastatin, which were not included in this review. |
| 11 | Wani ZA, Mohapatra S, Khan AA, Mohapatra A, Yatoo GN. Addition of simvastatin to carvedilol non responders: A new pharmacological therapy for treatment of portal hypertension. *World J Hepatol*. 2017;9(5):270-277. doi:10.4254/wjh.v9.i5.270 | Wrong comparator - The study did not include a separate, concurrent control group. The analysis was a single-arm, pre-post comparison where patients served as their own controls. |
| 12 | Mahmud N, Chapin S, Goldberg DS, Reddy KR, Taddei TH, Kaplan DE. Statin exposure is associated with reduced development of acute-on-chronic liver failure in a Veterans Affairs cohort. *J Hepatol*. 2022;76(5):1100-1108. doi:10.1016/j.jhep.2021.12.034 | No outcomes of interest - Study evaluated the risk of development of  acute-on-chronic liver failure (ACLF), which was not included in this review as an outcome of interest. |

**eTable 3:** Characteristics of included studies: reported outcomes and statin administration.

| **Author (year)** | **Outcomes** | **Statin Administration** |
| --- | --- | --- |
| **RCT** |  |  |
| Abraldes 2009 | Mortality  Ascites  HVPG | Simvastatin 20 mg/day for 1 month; increased to 40 mg/day after 15 days. |
| Pollo-Flores 2015 | HVPG | Simvastatin 20 mg/day for 15 days; increased to 40 mg/day thereafter; continued for a total of 3 months. |
| Abraldes 2016 | Mortality  Variceal bleeding  Ascites  Hepatorenal Syndrome  SBP  Hepatic Encephalopathy | Simvastatin 20 mg/day for 15 days; increased to 40 mg/day thereafter; continued up to 2 years. |
| Bishnu 2018 | Mortality  Variceal bleeding  SBP  Hepatic Encephalopathy  HVPG | Atorvastatin 20 mg/day plus propranolol 40 mg/day. |
| Elwan 2018 | Ascites | Simvastatin 20 mg/day for 2 weeks; increased to 40 mg/day for 2 weeks. |
| Jha 2019 | Mortality  Variceal bleeding  Ascites  Hepatorenal Syndrome  SBP  Hepatic Encephalopathy | Simvastatin 20 mg/day; increased to 40 mg/day after 7 days if tolerated; combined with carvedilol up to 12.5 mg/day plus EVL. |
| Vijayaraghavan 2020 | Mortality  Variceal bleeding  Ascites  HVPG | Simvastatin 20 mg/day at night; increased to 40 mg/day after 15 days if tolerated; continued 3 months. Combined with carvedilol 3.125 mg twice daily, titrated to 12.5 mg twice daily. |
| Kronborg 2023 | Mortality  Ascites  Hepatorenal Syndrome  SBP  Hepatic Encephalopathy  HVPG | Atorvastatin 10 mg/day; increased to 20 mg/day after 1 month if tolerated; continued 6 months. |
| Alvarado-Tapias 2025 | HVPG | Simvastatin 20 mg/day; increased to 40 mg/day after 2 weeks if no safety concerns; maintained for chronic response assessment. |
| **Observational studies** |  |  |
| Kumar 2014 | Mortality  Decompensation | Various statins, continued ≥3 months after biopsy (specific drug/dose not reported). |
| Mohanty 2016 | HCC  Variceal Bleeding  Mortality  Decompensation  Ascites  SBP | Simvastatin (85%), lovastatin (10%), pravastatin (3%), rosuvastatin (1%), fluvastatin (1%). |
| Bang 2017 | Mortality  Decompensation | Statin use defined as ≥2 prescriptions; treatment start at second claim; total duration >1 month. |
| Chang 2017 | HCC  HE  Variceal Bleeding  Mortality  Decompensation | Statin users defined as patients with cumulative defined daily dose (cDDD) ≥28. |
| Corey 2017 | HCC | Not reported. |
| Patel 2018 | HE  Variceal Bleeding  Mortality | Statin exposure defined up to 6 months after therapy initiation. |
| Kaplan 2019 | HCC  Mortality  Decompensation | Any statin initiated during follow-up (VA pharmacy), modeled as a time-varying exposure. |
| Hung 2019 | Mortality | Atorvastatin, rosuvastatin, fluvastatin, simvastatin, pravastatin, lovastatin, or pitavastatin. |
| Goh 2020 | HCC | Not reported. |
| Merkel 2021 | HCC  Hepatorenal Syndrome  HE  Variceal Bleeding  Decompensation  Ascites | Simvastatin (63.0%), atorvastatin (28.3%), pravastatin (5.8%), fluvastatin (2.9%). Mean simvastatin-equivalent dose: 35.2 ± 22.1 mg/day. |
| Pinyopornpanish 2021 (a) | HCC | Not reported. |
| Pinyopornpanish 2021 (b) | HCC | Hydrophilic and lipophilic statins. |
| Kraglund 2023 | HCC | Continuous prescriptions; exposure persisted until 7 days after tablets ran out. |
| Pfisterer 2024 (1) | Variceal Bleeding  Mortality | Not reported. |
| Pfisterer 2024 (2) | Variceal Bleeding  Mortality | Not reported. |
| Choi 2025 | HCC  Decompensation | Hydrophilic and lipophilic statins. |
| Cooper 2025 | Mortality | Statin use defined as having an active prescription on medication reconciliation at liver transplantation evaluation. |

LEGEND: EVL = Endoscopic variceal ligation; HCC = Hepatocellular carcinoma; HE = Hepatic encephalopathy; HVPG = Hepatic venous pressure gradient; SBP = Spontaneous bacterial peritonitis.

**eTable 4:** Covariates adjusted for in the multivariable analyses (adjusted hazard ratios) of included studies.

| **Author (year)** | **Covariates** |
| --- | --- |
| **Observational studies** |  |
| Kumar 2014 | **Mortality:** MELD score, CAD, diabetes, NASH, HCC.  **Decompensation:** Albumin, beta-blocker use, MELD score, NASH, HCC. |
| Mohanty 2016 | **Mortality, Decompensation and HCC:** Age, FIB-4 index score, serum level of albumin, MELD score, and BMI. |
| Bang 2017 | **Mortality**: Age, year of cohort entry, sex, socioeconomic status, Charlson index score, use of diuretics or nonselective beta-blockers, smoking, alcohol intoxication, healthy adherer profile, and indication of statins (stroke, ischaemic heart disease, and hypertension).  **Decompensation:** Adjusted by the covariates listed above but without use of diuretics. |
| Chang 2017 | **Mortality, Decompensation and HCC:** Age, sex, cirrhosis with different etiologies, comorbidities (DM, CAD, and hypertensive cardiovascular disease), medications (angiotensin-converting enzyme inhibitor, aspirin, other lipid-lowering drugs, antiviral drugs, and metformin), presence of nonhemorrhagic varices at enrollment, follow-up duration, and cirrhosis etiology. |
| Kaplan 2019 | **Mortality, Decompensation and HCC:** CTP class, disease etiology, age, sex, race-ethnicity, MELD-Na score, tobacco use, AUDIT-C-defined alcohol abuse, treating facility characteristics (academic affiliation, frequency of low socioeconomic status), DM, CAD, pre-cirrhosis statin exposure (if present), baseline or time-updating AST, ALT, total serum cholesterol, LDL cholesterol, triglycerides, and platelet counts. |
| Hung 2019 | **Mortality:** Age, sex, HCC, RFI, DM, etiology of cirrhosis (alcoholism, HBV, HCV), liver reserve, steroid use, and socioeconomic status. |
| Goh 2020 | **HCC:** Age, sex, cirrhosis, diabetes, hypertension, serum ALT, cholesterol, HBV DNA level, antiviral treatment, and antiplatelet therapy. |
| Pinyopornpanish 2021 (b) | **HCC:** Age, sex, race, decompensation status, smoking status, alcohol use status, DM, BMI, and MELD-Na score. |
| Kraglund 2023 | **HCC:** Age, sex, calendar year, time since cirrhosis diagnosis, cumulative prior use of statins, hospital contacts, liver imaging, decompensated cirrhosis, indications and relative contraindications for use of statins, potentially confounding drugs, and relapse of hazardous alcohol use. |
| Pfisterer 2024 (1) | **Mortality:** Age, CTP class and the use of co-medications. |
| Pfisterer 2024 (2) | **Mortality:** Age, CTP class and the use of co-medications. |
| Choi 2025 | **Decompensation and HCC:** Age, sex, race and ethnicity, causes of chronic liver disease, body mass index, presence of cirrhosis, DM, hypertension, CAD, peripheral vascular disease, dyslipidemia, chronic kidney disease, cerebrovascular accident, Fibrosis-4 score, and use of medications (metformin, aspirin, fibrate, nicotinic acid, bile acid sequestrant, sodium-glucose cotransporter-2 inhibitor, oral antiviral therapy, and interferon). |
| Cooper 2025 | **Mortality:** Age, MELD-Na, BMI, and CAD. |

LEGEND: ALT = alanine aminotransferase; AST = aspartate aminotransferase; AUDIT-C = Alcohol Use Disorders Identification Test–Consumption; BMI = body mass index; CAD = coronary artery disease; CTP = Child–Turcotte–Pugh; DM = diabetes mellitus; FIB-4 = Fibrosis-4 index; HBV = hepatitis B virus; HCC = hepatocellular carcinoma; HCV = hepatitis C virus; LDL = low-density lipoprotein; MELD = Model for End-Stage Liver Disease; MELD-Na = Model for End-Stage Liver Disease–Sodium; NASH = nonalcoholic steatohepatitis; RFI = renal function impairment.

**eTable 5:** GRADE table.

| Certainty assessment | | | | | | | | № of patients | | Effect | | Certainty |
| --- | --- | --- | --- | --- | --- | --- | --- | --- | --- | --- | --- | --- |
| № of studies | Study design | Risk of bias | Inconsistency | Indirectness | Imprecision | Publication bias | Other considerations | Intervention | Control | Relative (95% CI) | Absolute (95% CI) |  |
| **All-cause mortality (RCTs)** | | | | | | | | | | | | |
| 6 | randomised trials | serious^a^ | not serious | not serious | serious^b^ | none | none | 323 | 338 | OR 0.45 [0.25; 0.82] | 61 fewer per 1,000  (from 86 fewer to 19 fewer) | ⨁⨁OO Low |
| **All-cause mortality (Observational)** | | | | | | | | | | | | |
| 9 | non-randomised studies | serious^c^ | serious^d^ | not serious | not serious | none | none | 9162 | 20746 | HR 0.67 [0.54; 0.83] | 75 fewer per 1,000  (from 104 fewer to 39 fewer)^e^ | ⨁OOO Very low |

**Hepatic Decompensation (RCTs)**

| 7 | randomised trials | serious^f^ | not serious | not serious | serious^g^ | none | none | 343 | 358 | OR 0.75 [0.52; 1.09] | 58 fewer per 1,000  (from 105 fewer to 16 more) | ⨁⨁OO Low |
| --- | --- | --- | --- | --- | --- | --- | --- | --- | --- | --- | --- | --- |

**Hepatic Decompensation (Observational)**

| 6 | non-randomised studies | serious^h^ | serious^i^ | not serious | not serious | none | none | 8353 | 20161 | HR 0.58 [0.42; 0.80] | 90 fewer per 1,000  (from 124 fewer to 43 fewer)^j^ | ⨁OOO Very low |
| --- | --- | --- | --- | --- | --- | --- | --- | --- | --- | --- | --- | --- |

**Hepatocellular carcinoma (Observational)**

| 7 | non-randomised studies | serious^k^ | serious^l^ | not serious | not serious | none | none | 10942 | 38038 | HR 0.61 [0.46; 0.82] | 27 fewer per 1,000  (from 38 fewer to 13 fewer)^m^ | ⨁OOO Very low |
| --- | --- | --- | --- | --- | --- | --- | --- | --- | --- | --- | --- | --- |

**Hepatic venous pressure gradient (RCTs)**

| 6 | randomised trials | serious^n^ | not serious | not serious | serious^o^ | none | none | 188 | 195 | -  (not applicable) | Mean difference 1.14 mmHg lower  (1.71 lower to 0.56 lower) | ⨁⨁OO Low |
| --- | --- | --- | --- | --- | --- | --- | --- | --- | --- | --- | --- | --- |

**Hepatic Encephalopathy (RCTs)**

| 4 | randomised trials | serious^p^ | not serious | not serious | serious^q^ | none | none | 184 | 200 | OR 1.20 [0.38; 3.75] | 8 more per 1.000  (from 24 fewer to 95 more) | ⨁⨁OO Low |
| --- | --- | --- | --- | --- | --- | --- | --- | --- | --- | --- | --- | --- |

**Hepatic Encephalopathy (Observational)**

| 3 | non-randomised studies | serious^r^ | serious^s^ | not serious | serious^t^ | none | none | 848 | 877 | OR 0.67 [0.34; 1.31] | 45 fewer per 1,000  (from 94 fewer to 37 more) | ⨁OOO Very low |
| --- | --- | --- | --- | --- | --- | --- | --- | --- | --- | --- | --- | --- |

**Hepatorenal Syndrome (RCTs)**

| 3 | randomised trials | serious^u^ | not serious | not serious | serious^v^ | none | none | 172 | 187 | OR 1.04 [0.26; 4.22] | 1 more per 1.000  (from 15 fewer to 63 more) | ⨁⨁OO Low |
| --- | --- | --- | --- | --- | --- | --- | --- | --- | --- | --- | --- | --- |

**Ascites (RCTs)**

| 6 | randomised trials | serious^w^ | not serious | not serious | serious^x^ | none | none | 332 | 346 | OR 0.98 [0.60; 1.59] | 3 fewer per 1.000  (from 44 fewer to 57 more) | ⨁⨁OO Low |
| --- | --- | --- | --- | --- | --- | --- | --- | --- | --- | --- | --- | --- |

**Ascites (Observational)**

| 2 | non-randomised studies | serious^y^ | not serious | not serious | not serious | none | none | 839 | 2216 | OR 0.66 [0.48; 0.90] | 29 fewer per 1.000  (from 46 fewer to 9 fewer) | ⨁OOO Very low |
| --- | --- | --- | --- | --- | --- | --- | --- | --- | --- | --- | --- | --- |

**Spontaneous bacterial peritonitis (RCTs)**

| 4 | randomised trials | serious^Z^ | not serious | not serious | serious^aa^ | none | none | 183 | 199 | OR 0.24 [0.06; 0.98] | 38 fewer per 1.000  (from 47 fewer to 1 fewer) | ⨁⨁OO Low |
| --- | --- | --- | --- | --- | --- | --- | --- | --- | --- | --- | --- | --- |

**Variceal Bleeding (RCTs)**

| 4 | randomised trials | serious^bb^ | not serious | not serious | serious^cc^ | none | none | 255 | 269 | OR 0.68 [0.41; 1.15] | 49 fewer per 1.000  (from 95 fewer to 21 more) | ⨁⨁OO Low |
| --- | --- | --- | --- | --- | --- | --- | --- | --- | --- | --- | --- | --- |

**Variceal Bleeding (Observational)**

| 6 | non-randomised studies | serious^dd^ | serious^ee^ | not serious | serious^ff^ | none | none | 1582 | 3670 | OR 0.62 [0.34; 1.14] | 31 fewer per 1.000  (from 54 fewer to 11 more) | ⨁OOO Very low |
| --- | --- | --- | --- | --- | --- | --- | --- | --- | --- | --- | --- | --- |

Explanations:

1. Downgraded by one level. While three trials were assessed as low risk of bias (Abraldes 2009, Abraldes 2016, Bishnu 2018), three trials (Jha 2019, Vijayaraghavan 2020, Kronborg 2023) raised some concerns, specifically in Domain 3 (Bias due to missing outcome data). As these trials contribute significant weight to the analysis, the overall confidence is reduced.
2. Downgraded by one level. Although the confidence interval excludes unity, the total number of events (n = 58) is low, rendering the effect estimate fragile. Furthermore, the confidence interval is wide, ranging from a 75% reduction to a more modest 18% reduction, indicating that the precise magnitude of the benefit remains uncertain.
3. Downgraded by one level. The majority of included studies were assessed as having a serious risk of bias. The primary limitations were Bias due to confounding (Domain 1), observed in multiple studies (e.g., Kumar 2014, Bang 2017, Hung 2019, Kaplan 2019, Cooper 2025) despite statistical adjustments, and Bias in selection of participants (Domain 3) (e.g., Pfisterer 2024, Hung 2019, Cooper 2025), which raises concerns about the comparability of the cohorts.
4. Downgraded by one level. The statistical heterogeneity was considerable (I² = 89.1%), indicating significant variation in effect sizes across the observational studies that could not be fully explained.
5. Calculated using an assumed control group risk of 227 per 1,000 (22.7%). This baseline risk was derived from the observed event rate in the unadjusted observational analysis (1,801 events in 7,934 controls), as the studies included in the adjusted Hazard Ratio analysis did not consistently report raw event counts.
6. Downgraded by one level. While some trials were at low risk of bias, four of the seven included trials (Elwan 2018, Jha 2019, Vijayaraghavan 2020, Kronborg 2023) raised some concerns. These concerns were primarily in Domain 1 (Randomization process), for Elwan 2018, and Domain 3 (Missing outcome data), for Jha 2019, Vijayaraghavan 2020, Kronborg 2023.
7. Downgraded by one level. The 95% confidence interval crosses the line of no effect (unity), ranging from a substantial reduction in decompensation (OR 0.52) to a potential increase in risk (OR 1.09). This wide interval, consistent with both benefit and harm, prevents a definitive conclusion regarding efficacy.
8. Downgraded by one level. The majority of evidence contributed by studies was assessed as having moderate or serious limitations. The primary concern was bias due to confounding (Domain 1), which was rated as serious in multiple large cohorts (Kumar 2014, Bang 2017, Kaplan 2019). Additionally, bias in selection of the reported result (Domain 6) was a consistent issue, rated as moderate in nearly all included studies, except for Choi 2025.
9. Downgraded by one level. The I² value was extremely high (94.4%), indicating considerable heterogeneity in the magnitude of effect across studies that could not be fully explained.
10. Calculated using an assumed control group risk of 214 per 1,000 (21.4%). This baseline risk was derived from the observed event rate in the unadjusted observational analysis (1,868 events in 8,738 controls), as the studies included in the adjusted Hazard Ratio analysis did not consistently report raw event counts.
11. Downgraded by one level. While some included studies were at low risk of bias (Choi 2025, Goh 2020), significant limitations were identified in others included in the analysis. Bias due to confounding (Domain 1) was a major issue, rated as critical in Kraglund 2023 and serious in Kaplan 2019. Additionally, moderate concerns were noted across several studies regarding Bias in selection of participants (Domain 3) and Bias in selection of the reported result (Domain 6).
12. Downgraded by one level. The I² value was very high (86.4%), indicating considerable heterogeneity in the magnitude of effect across studies that could not be fully explained.
13. Calculated using an assumed control group risk of 70 per 1,000 (7.0%). This baseline risk was derived from the observed event rate in the unadjusted observational analysis (1,610 events in 22,918 controls), as the studies included in the adjusted Hazard Ratio analysis did not consistently report raw event counts.
14. Downgraded by one level. While three trials were at an overall low risk of bias (Abraldes 2009, Bishnu 2018 and Alvarado-Tapias 2025), concerns were identified in others. Specifically, Bias due to missing outcome data (Domain 3) was rated as high in Pollo-Flores 2015 and as some concerns in Vijayaraghavan 2020 and Kronborg 2023, as dropouts were not fully accounted for in the hemodynamic analysis.
15. Downgraded by one level. Although the confidence interval excludes zero (indicating a statistically significant reduction), the total sample size (n=383) is small. Furthermore, the magnitude of the reduction (MD -1.14 mmHg) is modest, and the lower bound of the confidence interval (-0.56 mmHg) represents a very small clinical effect that may not translate into a reduction in clinical events like variceal bleeding.
16. Downgraded by one level. While two trials were at low risk of bias (Abraldes 2016, Bishnu 2018), half of the included evidence came from studies with some concerns (Jha 2019, Kronborg 2023). The primary issue was Bias due to missing outcome data (Domain 3), as the handling of dropouts was not clearly described.
17. Downgraded by one level. The confidence interval is very wide (0.38 to 3.75), crossing the line of no effect and including both substantial benefit and substantial harm. Additionally, the total number of events is extremely low (only 17 events in total), making the estimate fragile.
18. Downgraded by one level. All included studies were assessed as having limitations. Bias due to confounding (Domain 1) was a critical issue, rated as serious in Patel 2018. Bias in selection of the reported result (Domain 6) was rated as moderate in Chang 2017 and Merkel 2021, and bias due to missing outcome data (Domain 3) was also a moderate concern in these studies.
19. Downgraded by one level. The I² value was substantial (64.4%), indicating considerable heterogeneity in the magnitude of effect across studies that could not be fully explained.
20. Downgraded by one level. The confidence interval is wide (0.34 to 1.31) and crosses the line of no effect, including the possibility of both a large reduction in risk and a clinically significant increase in risk.
21. Downgraded by one level. While Abraldes 2016 was assessed as low risk of bias, the other included trials (Jha 2019, Kronborg 2023) raised some concerns. The primary limitation was Bias due to missing outcome data (Domain 3), as the handling of missing data for this specific outcome was not explicitly clarified.
22. Downgraded by one level. The confidence interval is extremely wide (0.26 to 4.22), crossing the line of no effect and including the possibility of both large benefit and large harm. Furthermore, the total number of events is extremely low (only 8 events total), rendering the estimate highly unstable.
23. Downgraded by one level. While Abraldes 2009 and Abraldes 2016 were assessed as low risk of bias, the other included trials (Elwan 2018, Jha 2019, Vijayaraghavan 2020, Kronborg 2023) raised some concerns. The primary issues were Bias due to missing outcome data (Domain 3), rated as some concerns in Jha 2019, Vijayaraghavan 2020, and Kronborg 2023, and Bias arising from the randomization process (Domain 1), rated as some concerns in Elwan 2018.
24. Downgraded by one level. The confidence interval crosses the line of no effect (0.60 to 1.59) and includes the possibility of both a moderate reduction in risk and a substantial increase in risk. This uncertainty prevents a definitive conclusion regarding the effect of statins on ascites development.
25. Downgraded by one level. Both included studies were assessed as having moderate limitations overall. In Mohanty 2016, moderate concerns were noted in Bias due to confounding (Domain 1) and Bias in measurement of the outcome (Domain 5). In Merkel 2021, moderate concerns were noted in Bias in classification of interventions (Domain 2) and Bias in selection of participants (Domain 3). Additionally, Bias in selection of the reported result (Domain 6) was a consistent issue, rated as moderate in both studies.
26. Downgraded by one level. While Abraldes 2016 and Bishnu 2018 were assessed as low risk of bias, Jha 2019 and Kronborg 2023 raised some concerns. The primary limitation was Bias due to missing outcome data (Domain 3), in which the handling of dropouts was not clearly reported.
27. Downgraded by one level. Although the confidence interval excludes the line of no effect, the total number of events is extremely low (only 11 events in total). This low event count renders the effect estimate fragile, despite the statistical significance. Furthermore, the confidence interval is wide (0.06 to 0.98), ranging from a massive reduction in risk to a negligible benefit, indicating significant uncertainty regarding the true magnitude of the effect.
28. Downgraded by one level. While Abraldes 2016 and Bishnu 2018 were assessed as having low risk of bias, Jha 2019 and Vijayaraghavan 2020 were rated as raising some concerns. Specifically, both studies had some concerns in Domain 3 (bias due to missing outcome data).
29. Downgraded by one level. The confidence interval crosses the line of no effect (0.41 to 1.15), including the possibility of both a substantial reduction in bleeding risk and a potential increase in risk.
30. Downgraded by one level. All included studies were assessed as having moderate or serious limitations. The specific drivers varied by study: Bias due to confounding (Domain 1) was rated as serious in Patel 2018 and moderate in Mohanty 2016 and Pfisterer 2024. Bias in selection of participants (Domain 3) was the primary cause for the serious rating in Pfisterer 2024, while Chang 2017 and Merkel 2021 presented moderate concerns in this domain. Additionally, moderate concerns regarding Bias in selection of the reported result (Domain 6) were noted in Mohanty 2016, Chang 2017, and Merkel 2021.
31. Downgraded by one level. The statistical heterogeneity was substantial (I² = 56.1%), indicating significant variation in effect sizes across the observational studies that could not be fully explained.
32. Downgraded by one level. The confidence interval is wide (0.34 to 1.14) and crosses the line of no effect, including the possibility of both a substantial reduction in bleeding risk and a potential increase in risk.

**Notes:**

1 - Effect Measures and Absolute Risk Estimation:

- Observational Primary Outcomes: For all-cause mortality, hepatic decompensation, and hepatocellular carcinoma, adjusted hazard ratios (HRs) were prioritized to account for confounding. Because not all studies contributing adjusted HRs reported raw event counts, absolute effects were calculated by applying the pooled adjusted HR to a proxy baseline risk. This baseline risk was derived from the event rate in the full control group of the unadjusted observational analysis, which included all studies with available raw data.
- RCTs and Observational Secondary Outcomes: For RCTs, effect estimates were based on standard odds ratios (ORs) derived from event counts. For secondary observational outcomes (hepatic encephalopathy, hepatorenal syndrome, ascites, spontaneous bacterial peritonitis and variceal bleeding), unadjusted ORs were used. Absolute effects for these outcomes were estimated directly using the observed control group risks reported in the contributing studies.

2 - Excluded Outcomes: Observational data for Hepatorenal Syndrome and Spontaneous Bacterial Peritonitis were not reported in this summary table as evidence was limited to a single study for each, precluding meta-analytic synthesis.

**eText 1:** Detailed definitions of outcomes.

**Primary outcomes:** All-cause mortality was defined as death from any cause occurring during the follow-up period of each respective study. Hepatic decompensation was a composite outcome defined based on study design. For the included RCTs, hepatic decompensation was defined as the occurrence of any of the following five major clinical events: variceal bleeding, spontaneous bacterial peritonitis (SBP), hepatorenal syndrome (HRS), ascites, or hepatic encephalopathy (HE). For the included observational studies, the composite outcome of hepatic decompensation was extracted as defined by the original authors. While the precise components varied slightly across these studies, the definition consistently included ascites, variceal bleeding, or HE. The definition was broadened in several studies to also include SBP, HRS, and/or clinically significant jaundice. Ascertainment of these events in observational studies was based on clinical documentation, specific International Classification of Diseases (ICD) diagnostic codes, or procedural and pharmacological surrogates. Hepatocellular carcinoma (HCC) was defined as a new diagnosis of HCC identified through histopathological confirmation, established radiological criteria (such as Liver Imaging Reporting and Data System 5), or specific diagnostic codes (e.g., ICD-9 code 155.0; ICD-10 code C22.0x) as reported by the individual studies.

**Secondary outcomes:** Variceal bleeding was defined as clinically significant upper gastrointestinal bleeding, characterized by hematemesis or melena leading to hospitalization, with the source confirmed as esophageal or gastric varices by endoscopy or as identified by specific diagnostic codes. Ascites was defined as new-onset or worsening fluid accumulation in the peritoneal cavity, confirmed by clinical examination or abdominal imaging, often requiring the initiation or dose escalation of diuretic therapy, or as identified by specific diagnostic codes. Hepatic encephalopathy (HE) was defined as new-onset or worsening neurocognitive dysfunction, diagnosed clinically based on criteria such as the West Haven classification or as documented by a gastroenterologist, or as identified by specific diagnostic codes. Spontaneous bacterial peritonitis (SBP) and Hepatorenal Syndrome (HRS) were defined according to established clinical and laboratory criteria as reported in the source studies or as identified by specific diagnostic codes. The change in Hepatic Venous Pressure Gradient (HVPG) was defined as the absolute difference between the baseline measurement and the value measured at the end of the follow-up period. Each HVPG value was obtained via hepatic vein catheterization and calculated as the difference between the wedged hepatic venous pressure and the free hepatic venous pressure.

**eFigure 1:** Preferred Reporting Items for Systematic Reviews and Meta-analyses (PRISMA) flowchart of search strategy and included studies.


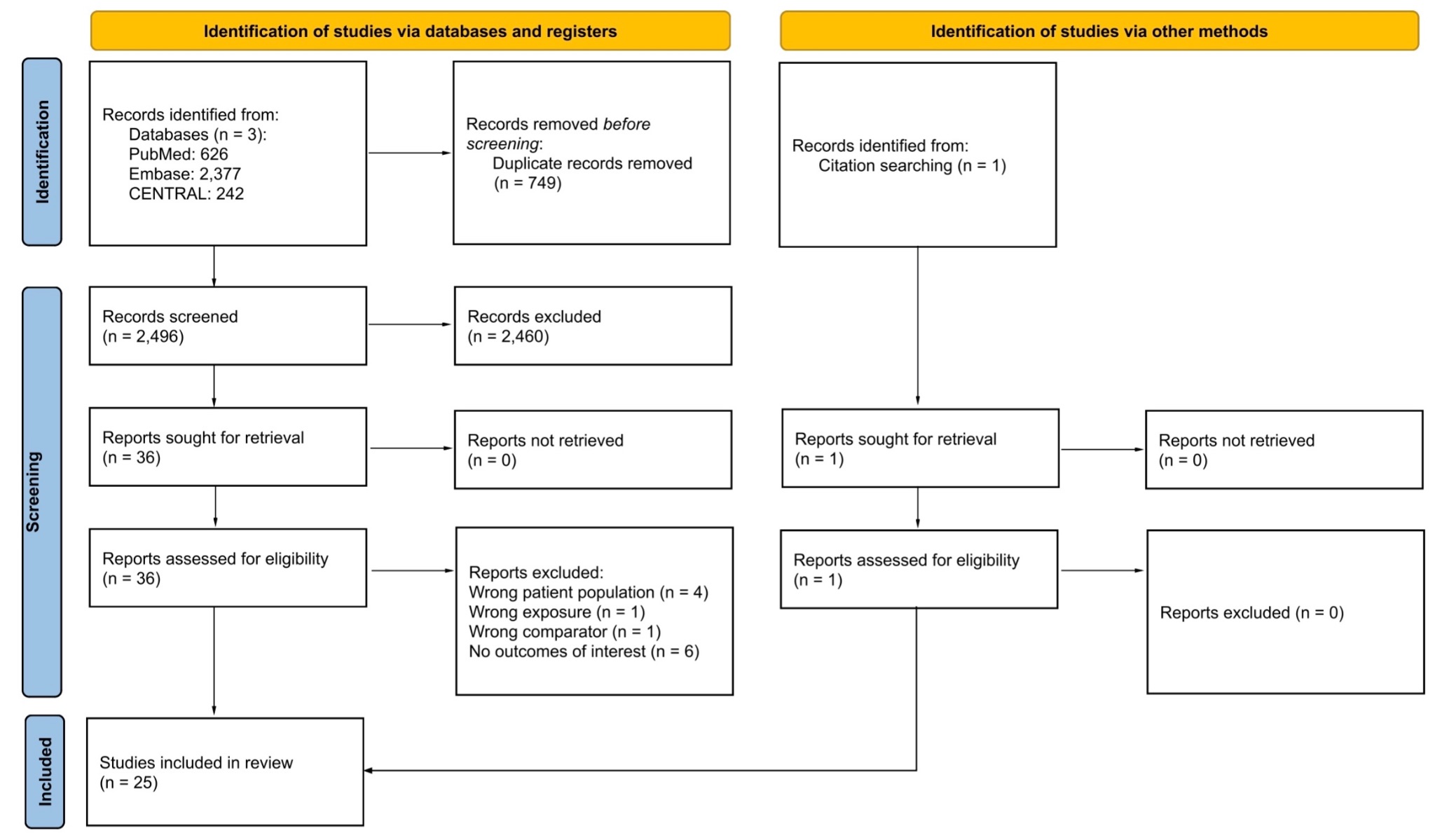


Source: Page MJ, McKenzie JE, Bossuyt PM, Boutron I, Hoffmann TC, Mulrow CD[, et al. The PRISMA 2020 statement: an updated guideline for reporting systematic reviews. *BMJ*. 2021;372:n71. doi:10.1136/bmj.n71](https://www.zotero.org/google-docs/?r1sax5). This work is licensed under CC BY 4.0. To view a copy of this license, visit https://creativecommons.org/licenses/by/4.0/

**eFigure 2**: Risk of bias assessments.

1. Primary outcome: All-cause mortality; assessed with Cochrane Risk of Bias tool - RoB 2 for randomized clinical trials.

*Traffic light plot*


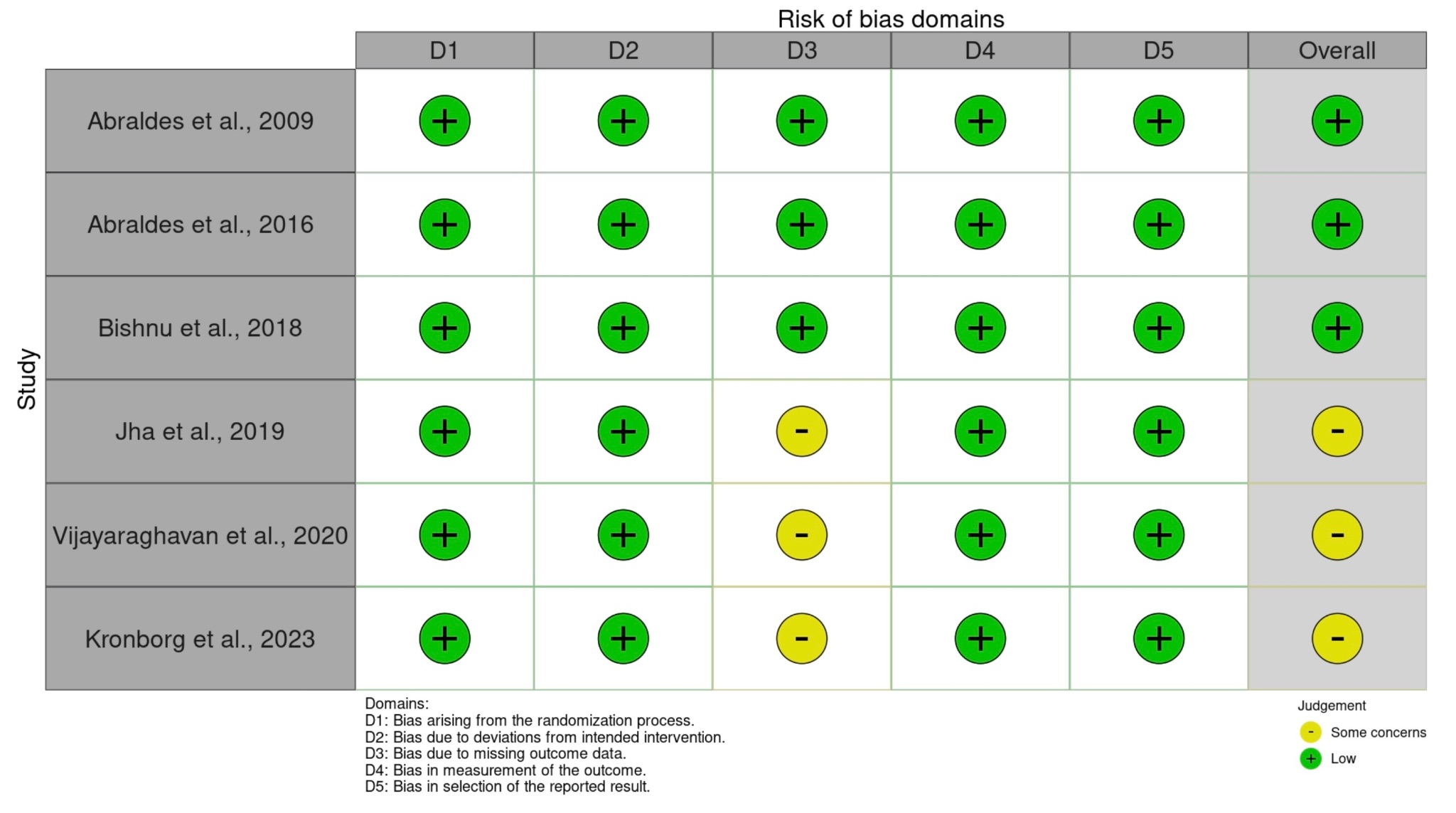


1. Primary outcome: All-cause mortality; assessed with Cochrane Risk of Bias tool - ROBINS-I V2 for observational studies.

*Traffic light plot*


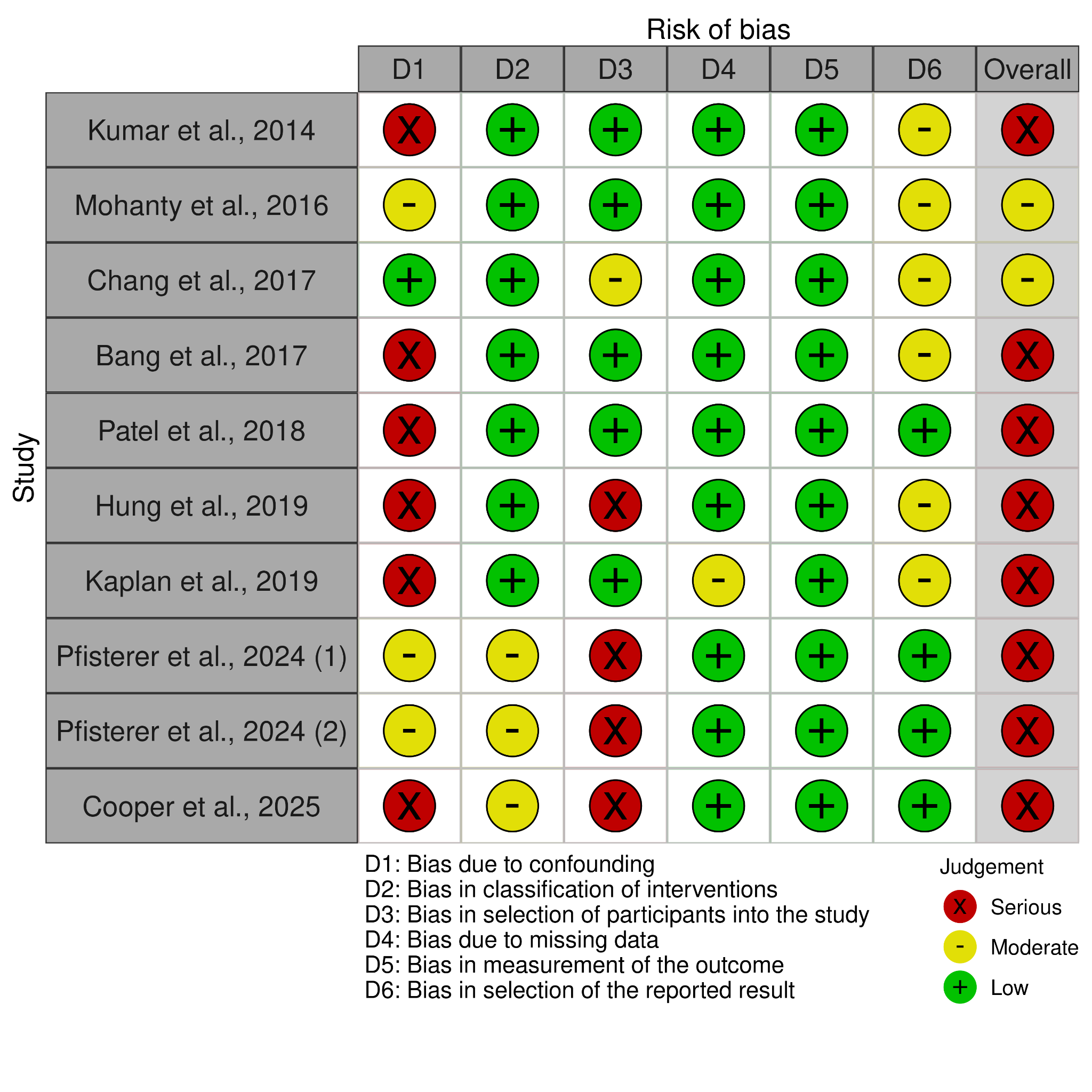


1. Primary outcome: Hepatic decompensation; assessed with Cochrane Risk of Bias tool - RoB 2 for randomized clinical trials

*Traffic light plot*


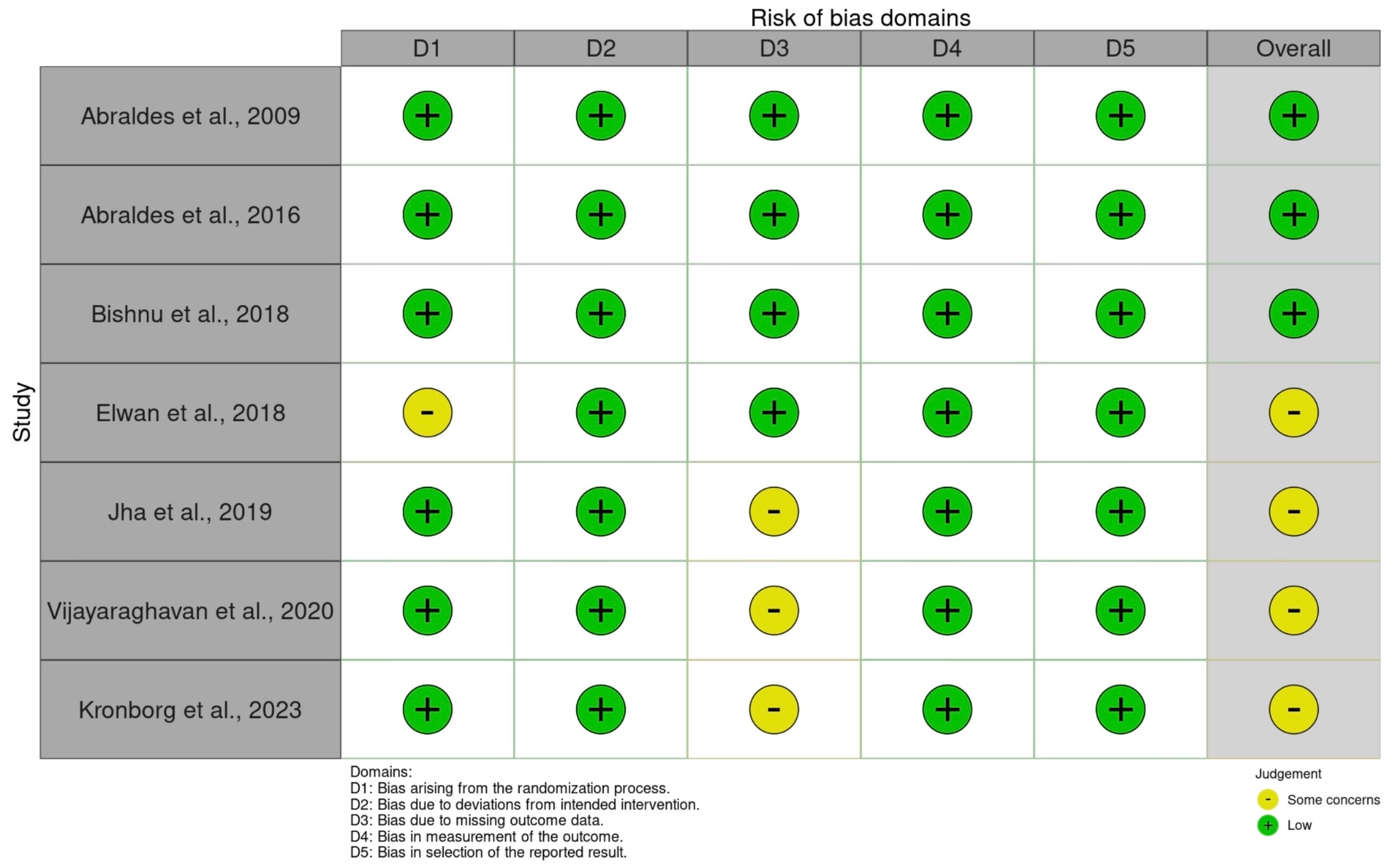


1. Primary outcome: Hepatic decompensation; assessed with Cochrane Risk of Bias tool - ROBINS-I V2 for observational studies.

*Traffic light plot*

*
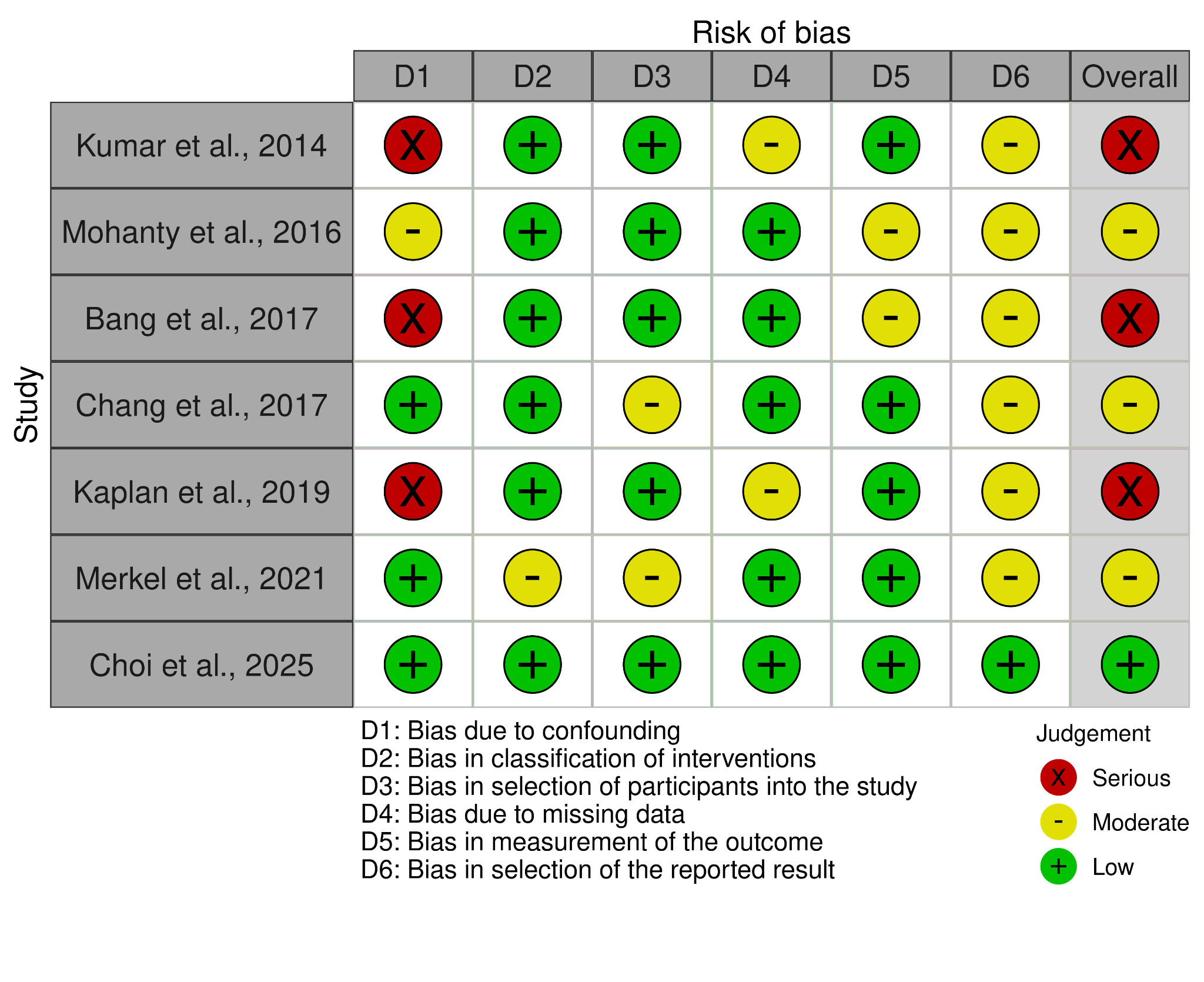
*

1. Primary outcome: Hepatocellular carcinoma; assessed with Cochrane Risk of Bias tool - ROBINS-I V2 for observational studies.

*Traffic light plot*


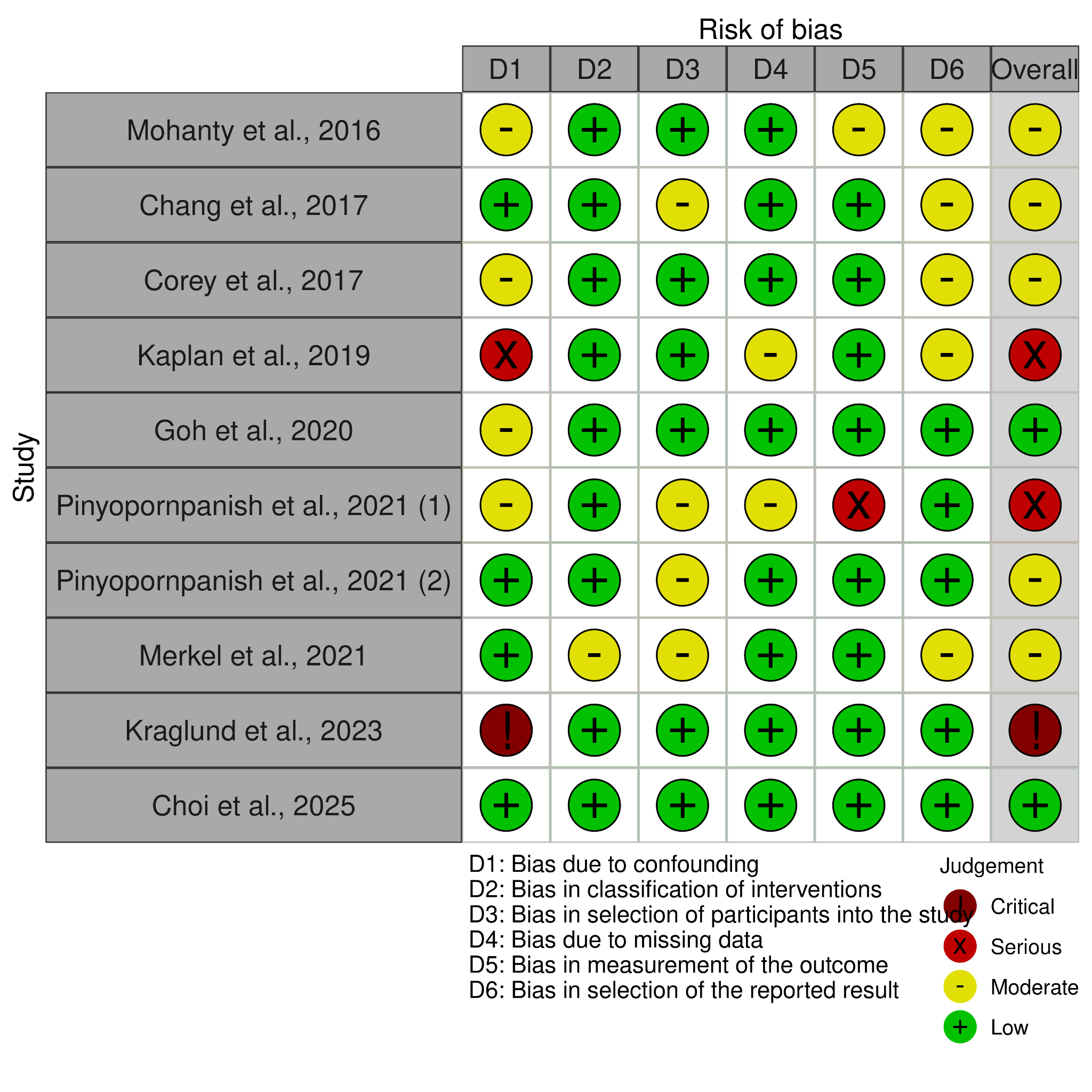


1. Secondary outcome: Variceal bleeding; assessed with Cochrane Risk of Bias tool - RoB 2 for randomized clinical trials

*Traffic light plot*


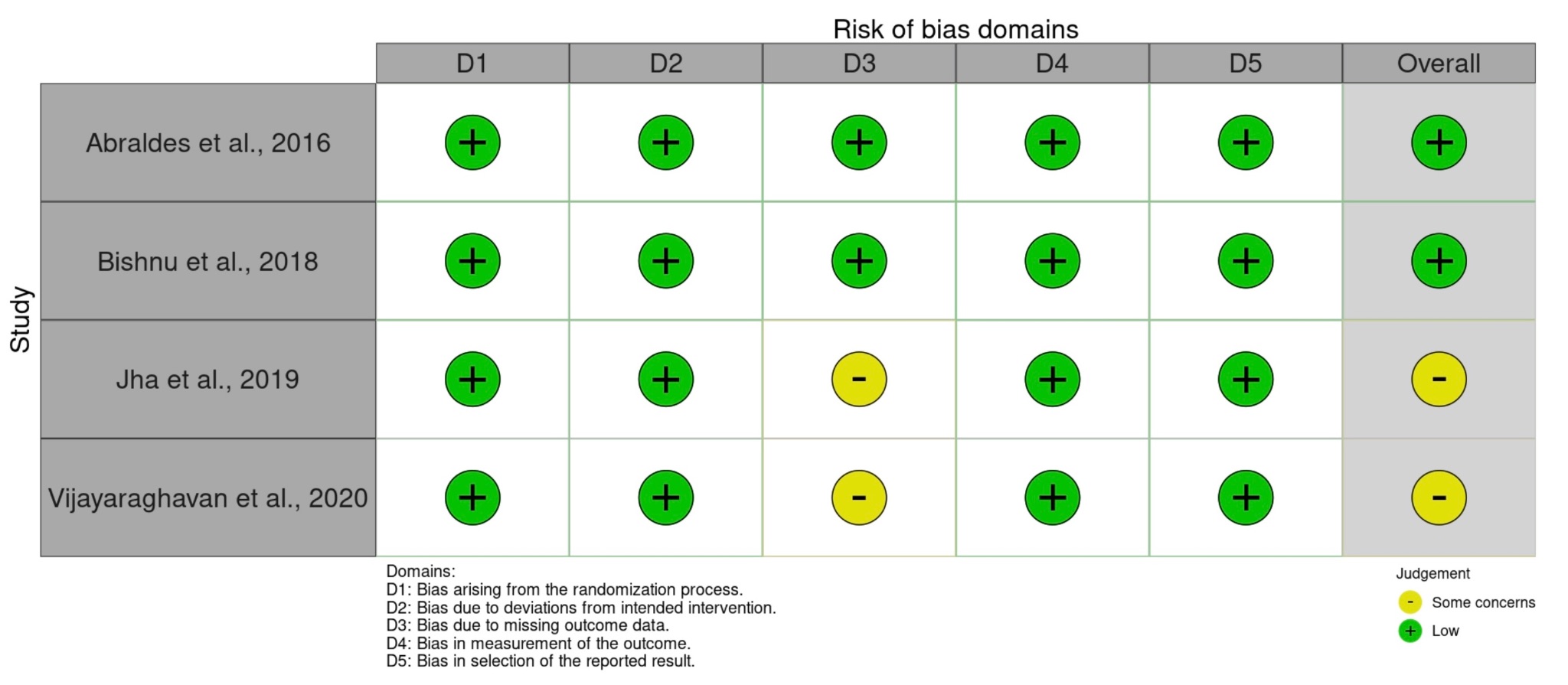


1. Secondary outcome: Variceal bleeding; assessed with Cochrane Risk of Bias tool - ROBINS-I V2 for observational studies.

*Traffic light plot*


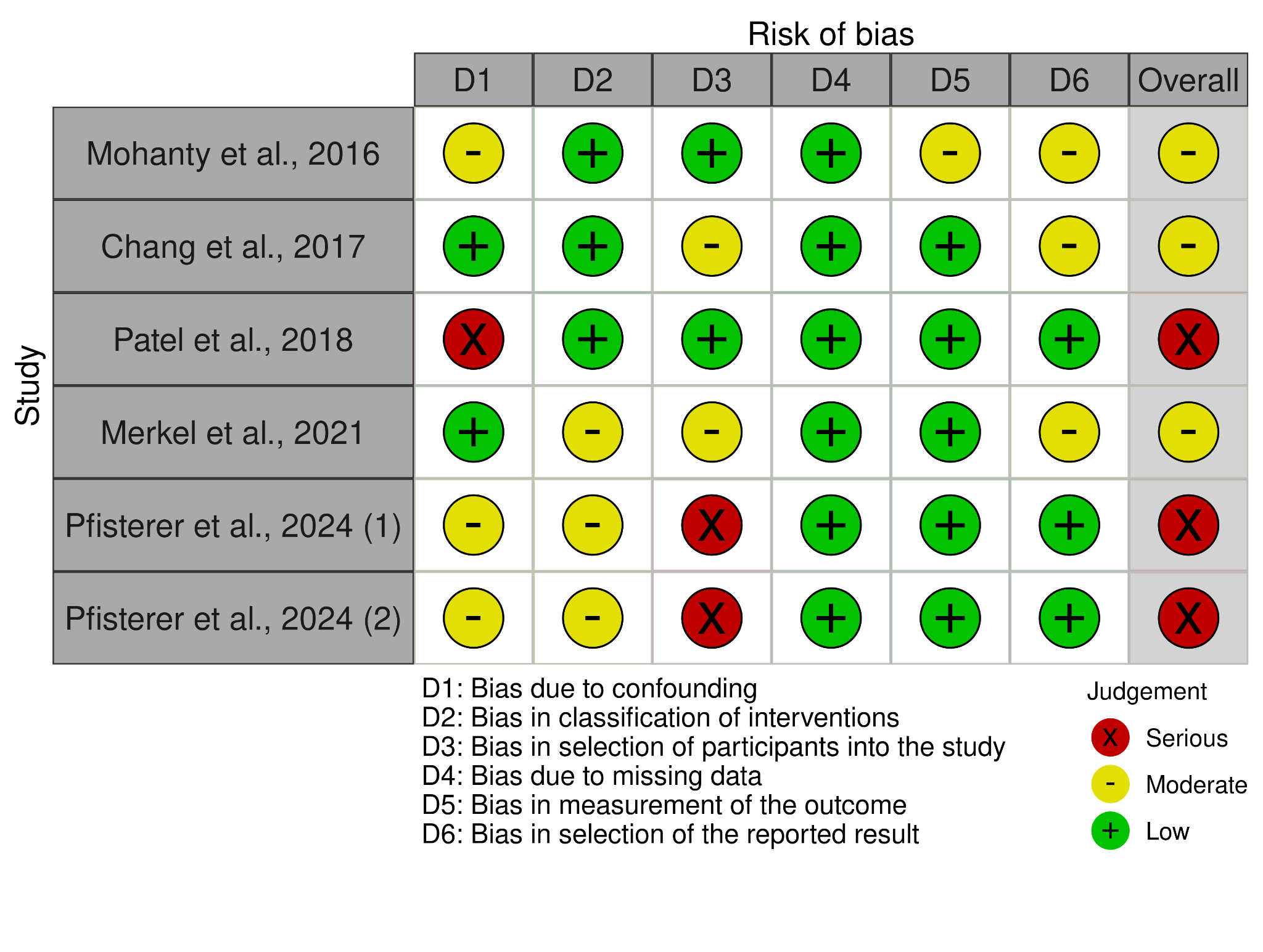


1. Secondary outcome: Ascites; assessed with Cochrane Risk of Bias tool - RoB 2 for randomized clinical trials.

*Traffic light plot*


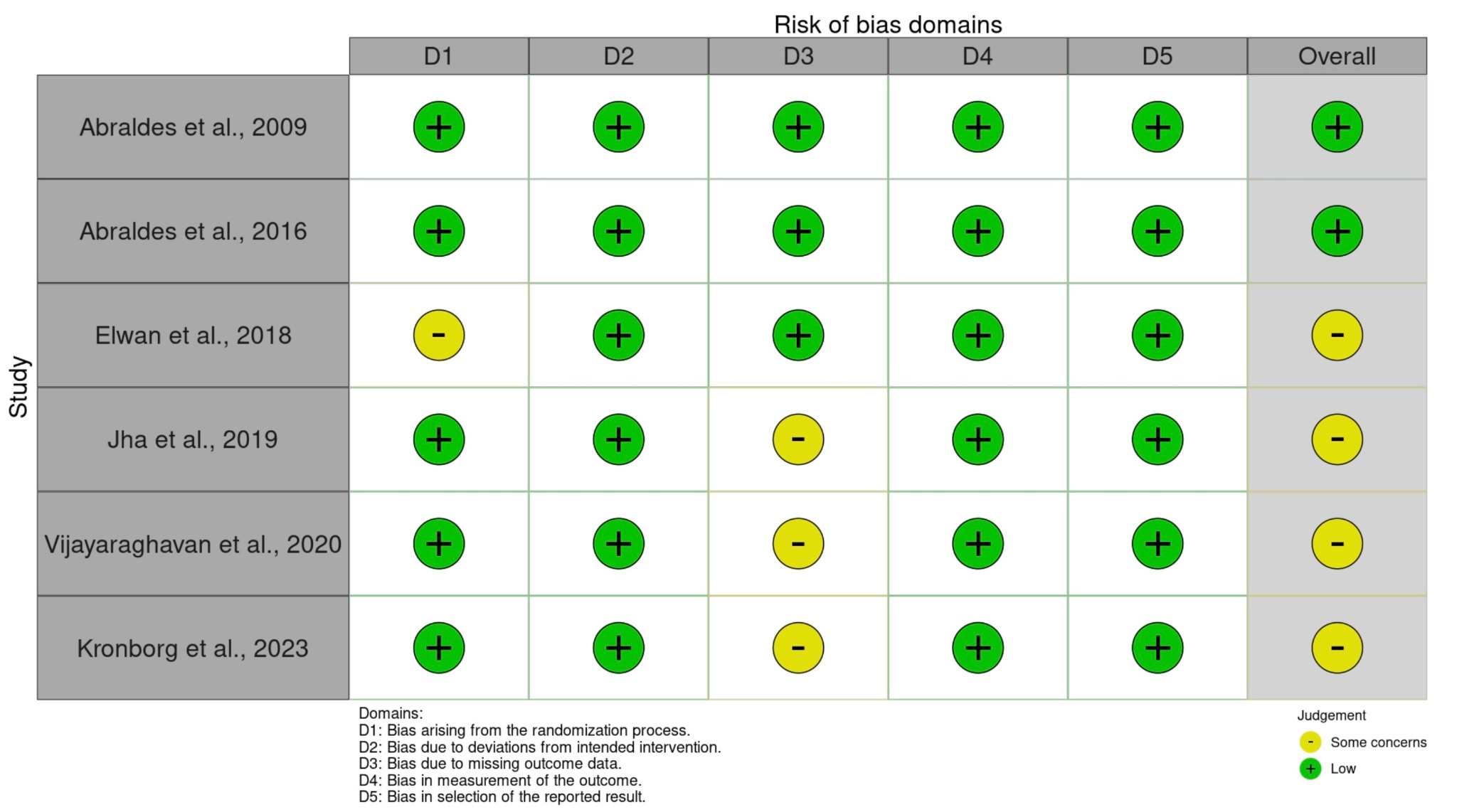


1. Secondary outcome: Ascites; assessed with Cochrane Risk of Bias tool - ROBINS-I V2 for observational studies.

*Traffic light plot*


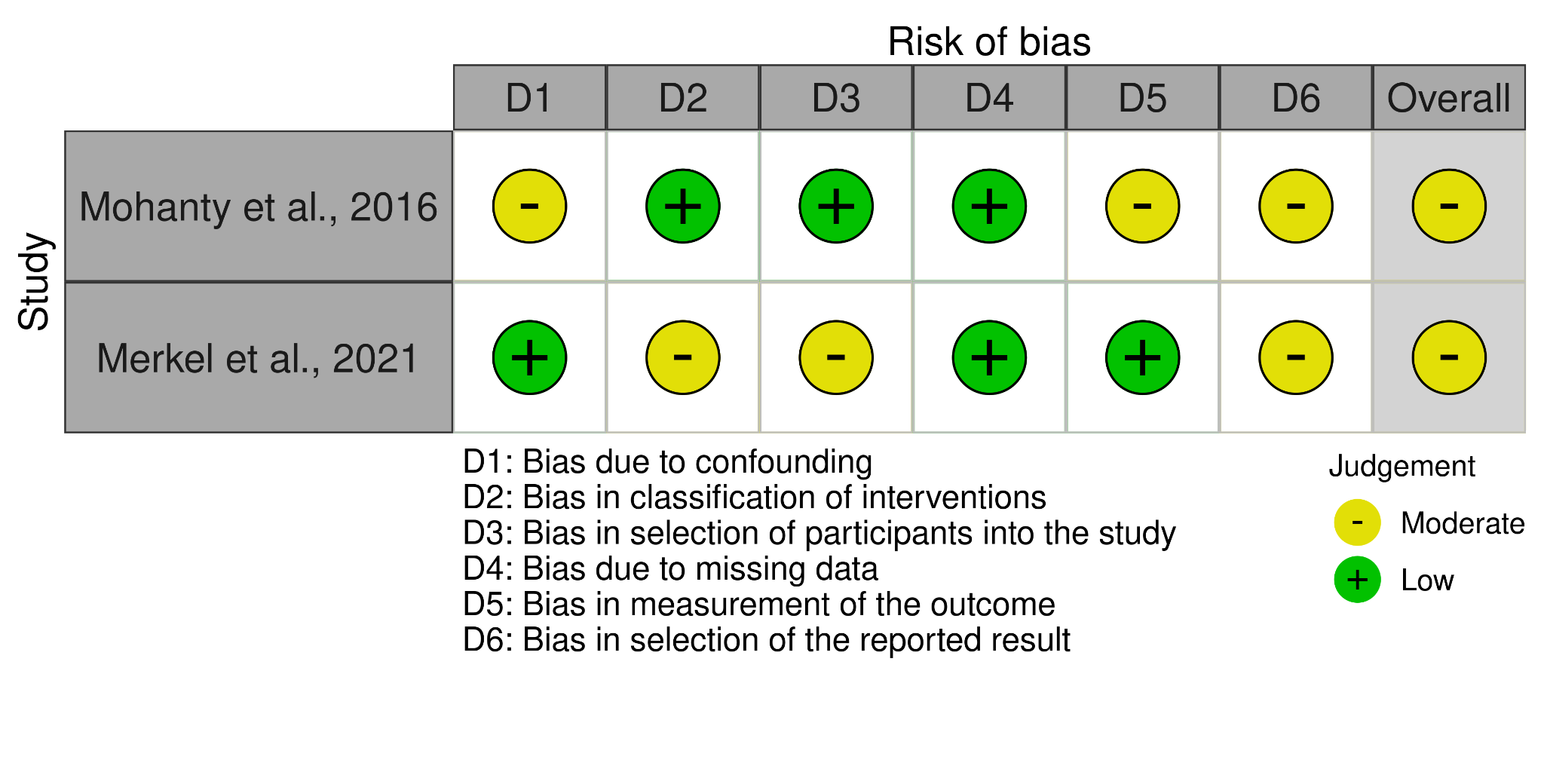


1. Secondary outcome: Hepatorenal syndrome; assessed with Cochrane Risk of Bias tool - RoB 2 for randomized clinical trials.

*Traffic light plot*


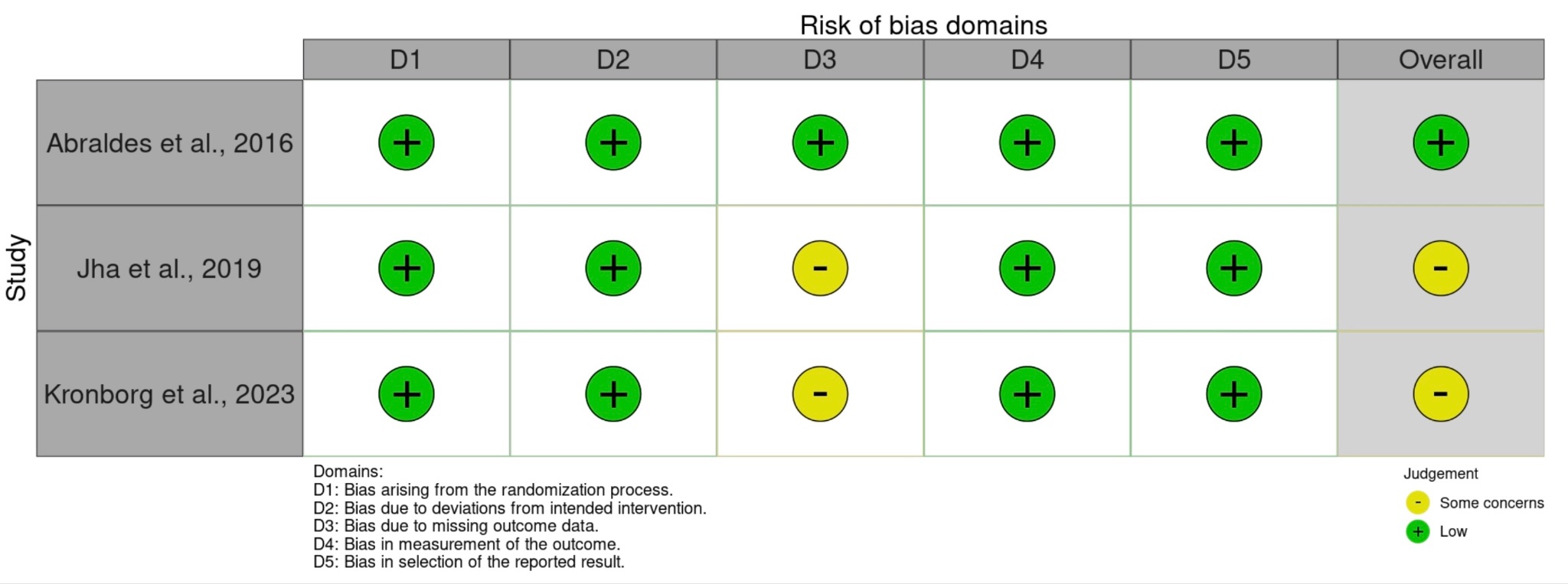


1. Secondary outcome: Hepatorenal syndrome; assessed with Cochrane Risk of Bias tool - ROBINS-I V2 for observational studies.

*Traffic light plot*


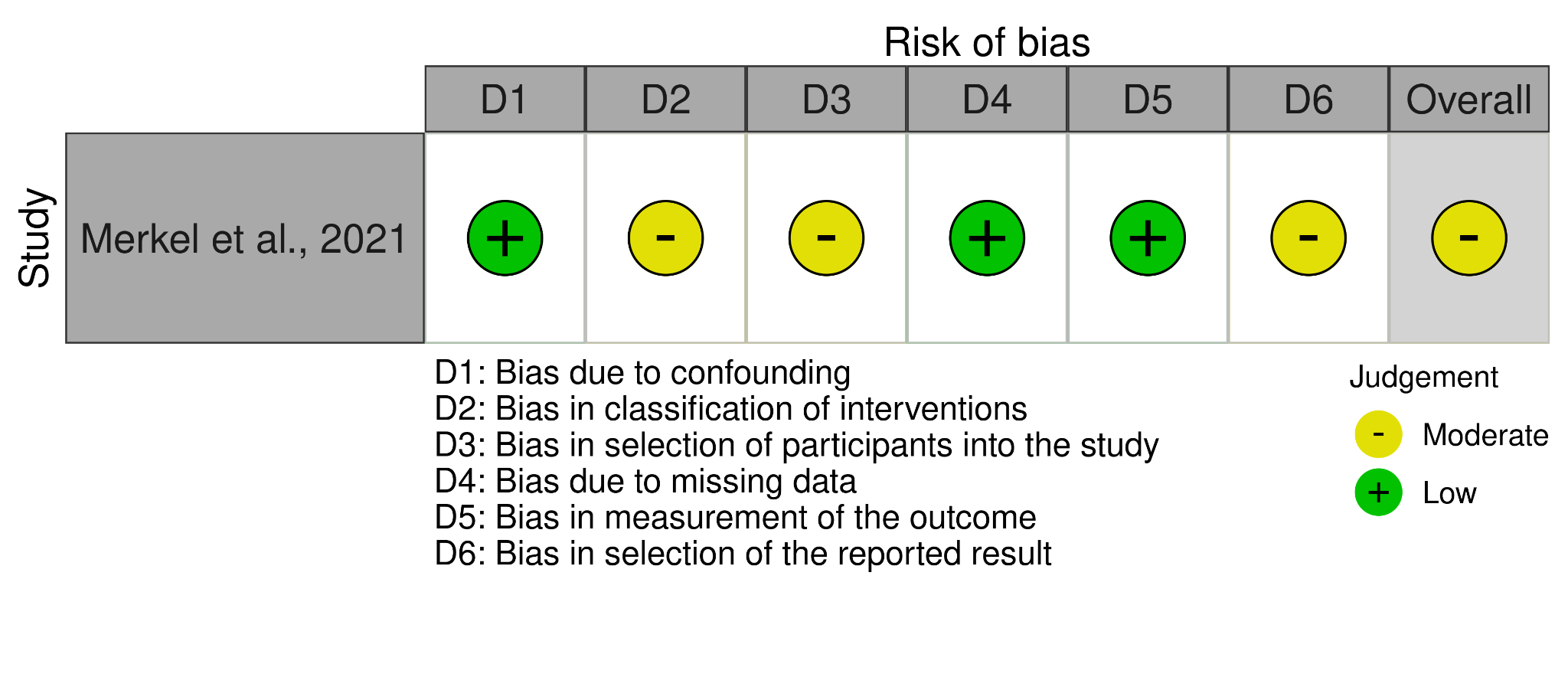


1. Secondary outcome: Spontaneous bacterial peritonitis; assessed with Cochrane Risk of Bias tool - RoB 2 for randomized clinical trials.

*Traffic light plot*


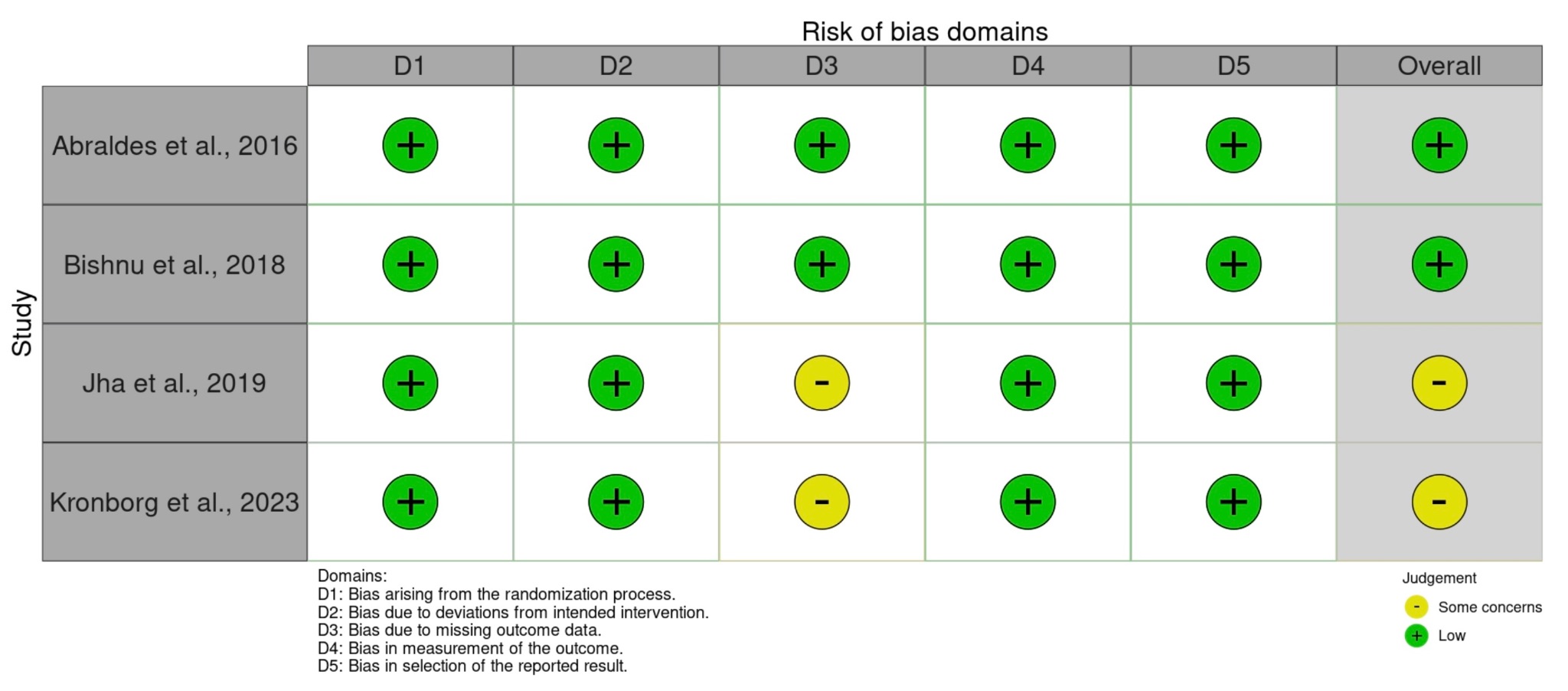


1. Secondary outcome: Spontaneous bacterial peritonitis; assessed with Cochrane Risk of Bias tool - ROBINS-I V2 for observational studies.

*Traffic light plot*

*
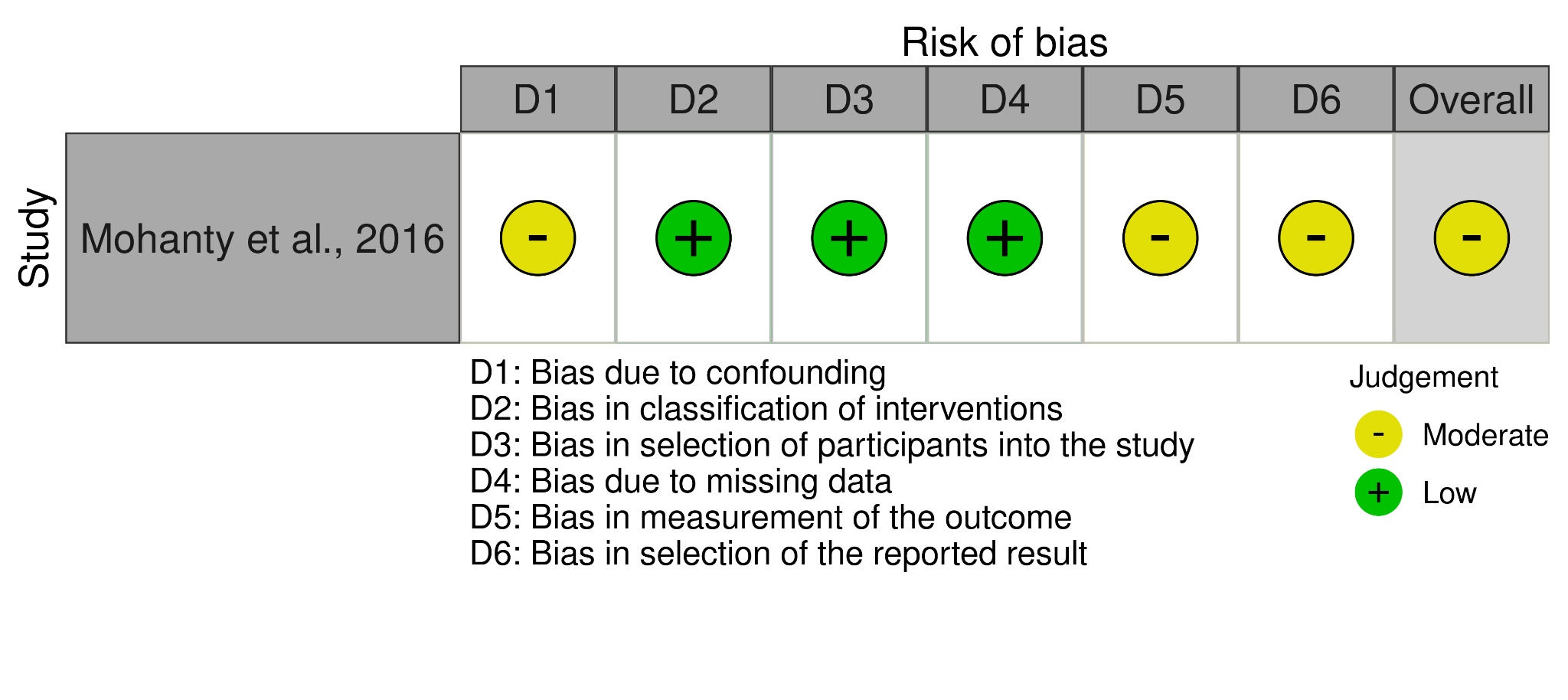
*

1. Secondary outcome: Hepatic encephalopathy; assessed with Cochrane Risk of Bias tool - RoB 2 for randomized clinical trials.

*Traffic light plot*


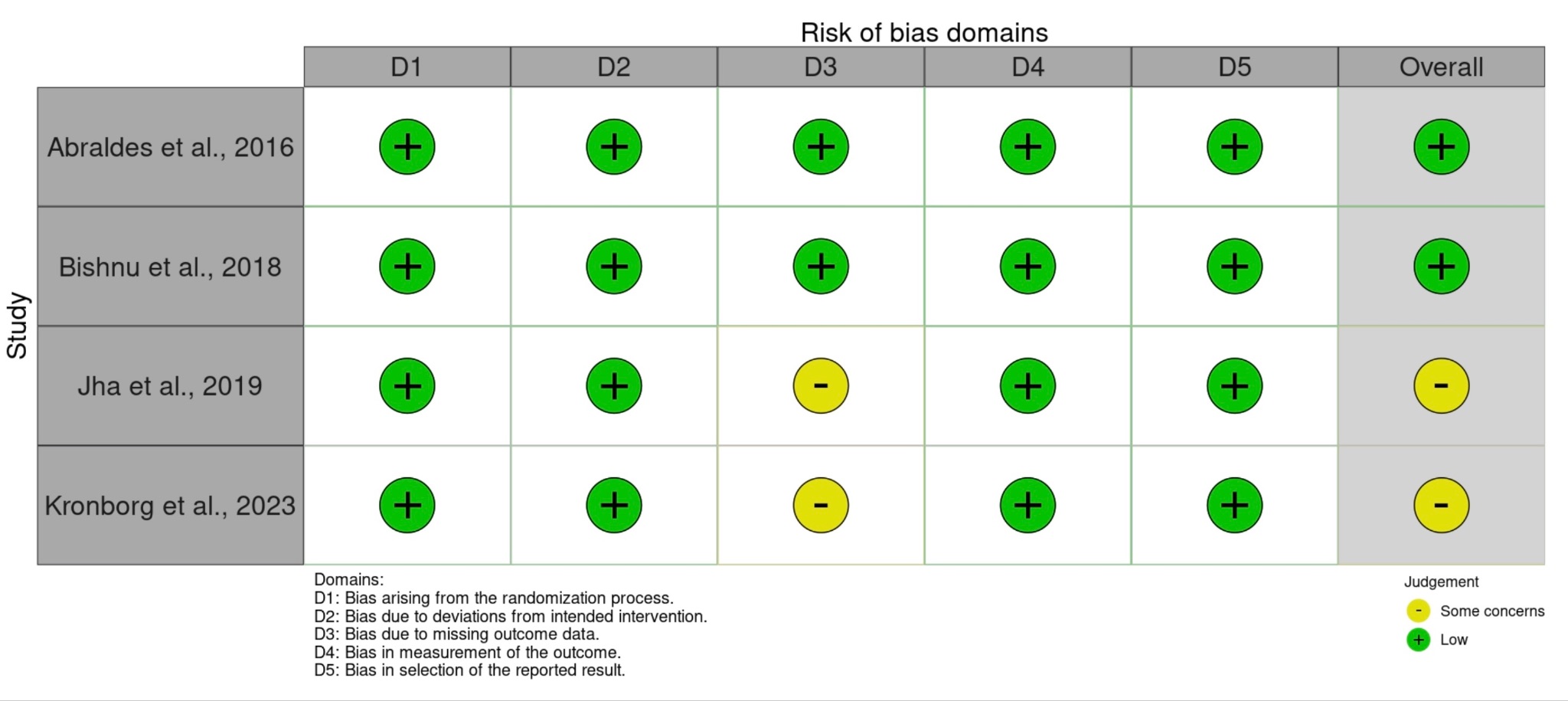


1. Secondary outcome: Hepatic encephalopathy; assessed with Cochrane Risk of Bias tool - ROBINS-I V2 for observational studies.

*Traffic light plot*


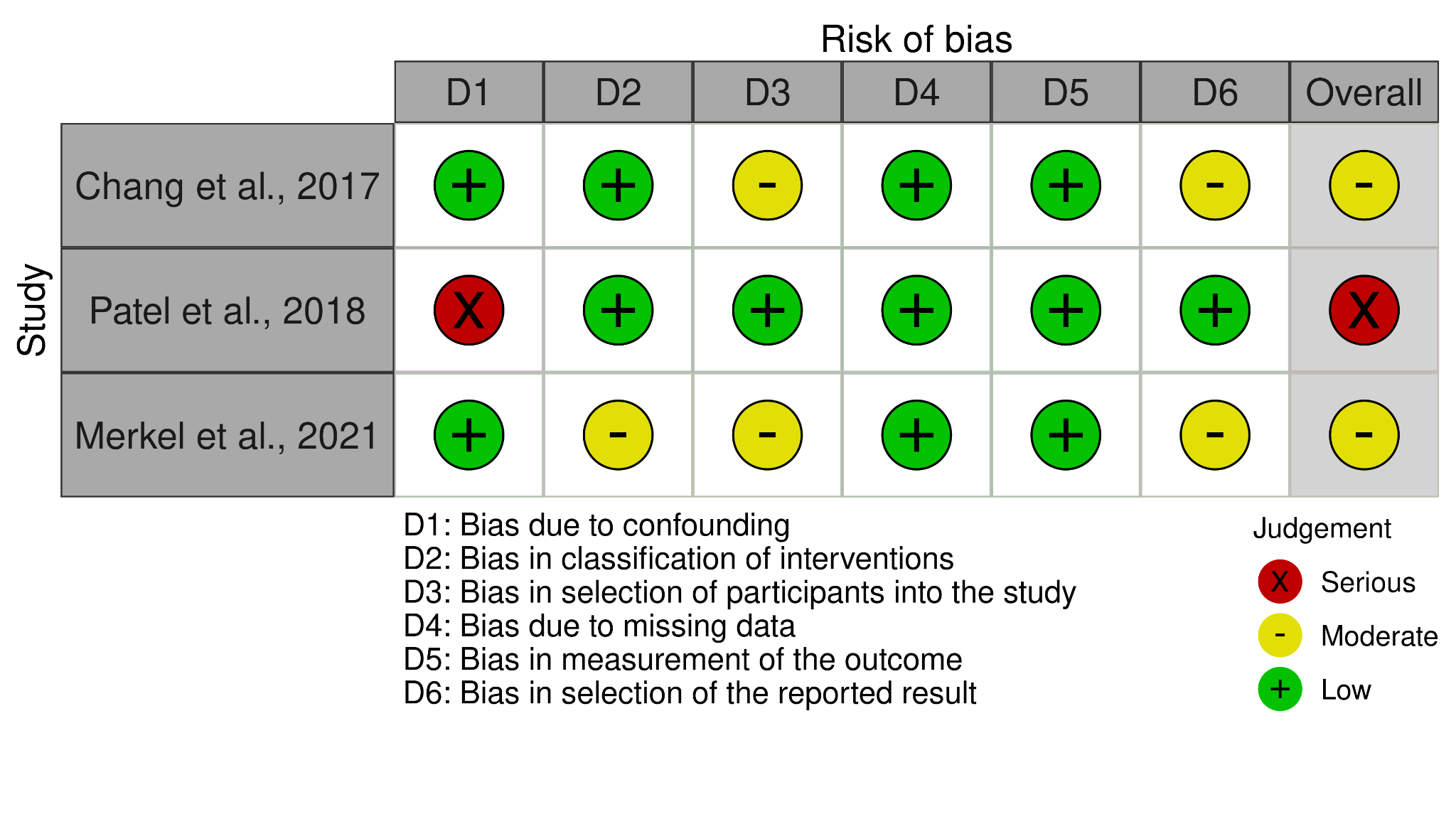


1. Secondary outcome: Change in hepatic venous pressure gradient (HVPG); assessed with Cochrane Risk of Bias tool - RoB 2 for randomized clinical trials.

*Traffic light plot*


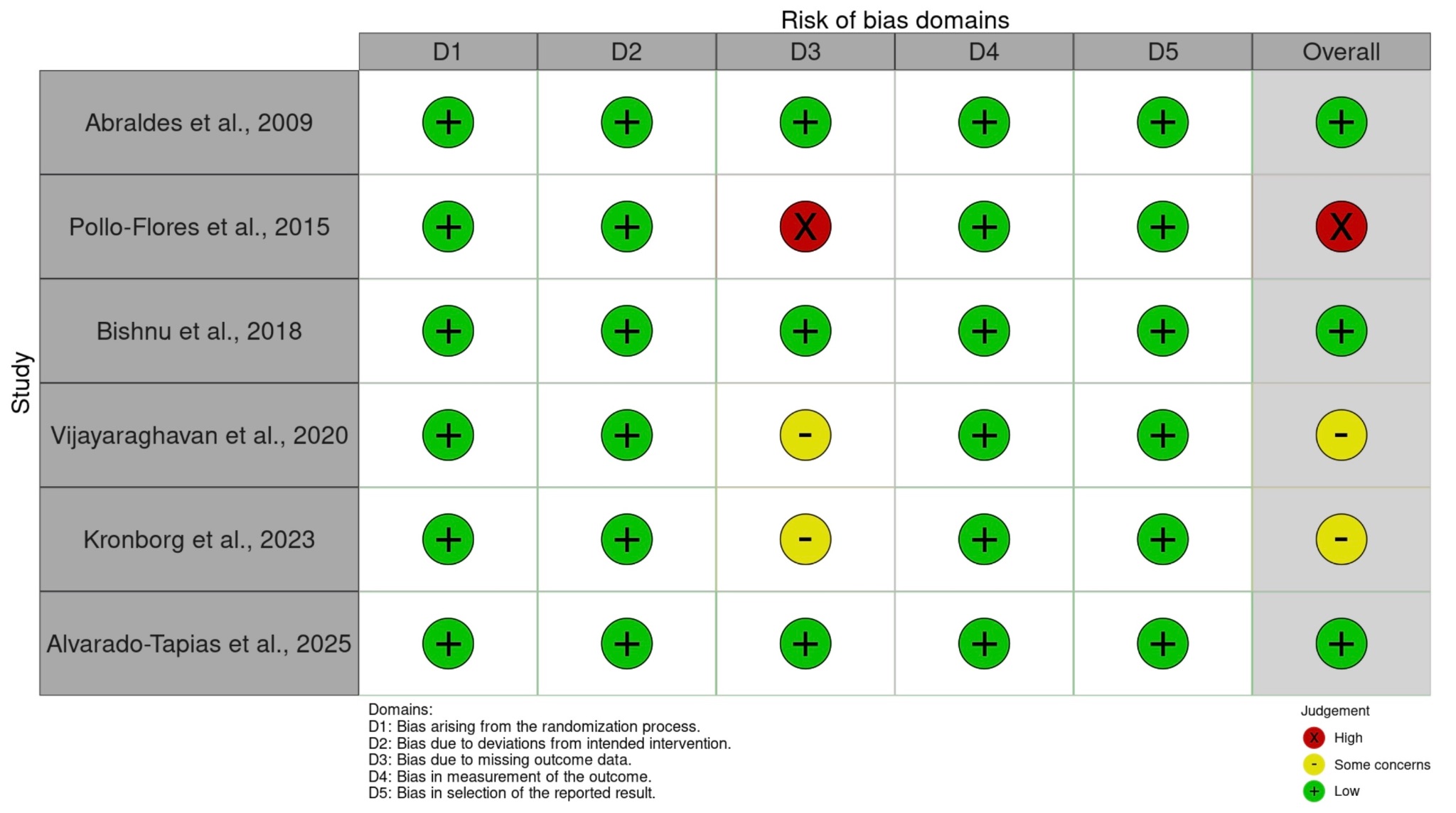


**eFigure 3:** Forest Plots.

1. **Primary outcome** - Effect of statins on hepatocellular carcinoma: Meta-analysis of unadjusted odds ratios from observational studies.


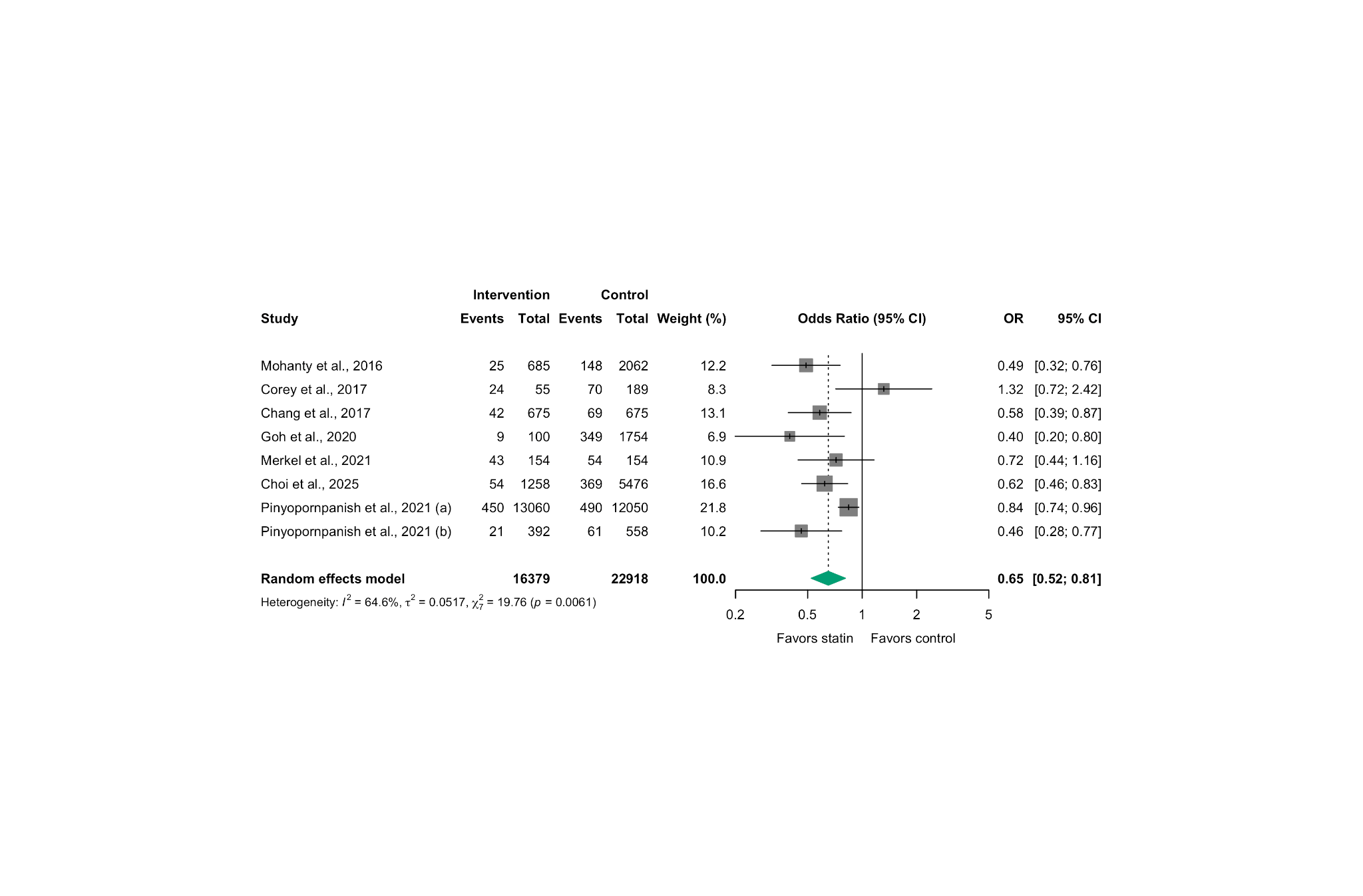


The squares represent point estimates, and the size of each square is proportional to the weight of the study. Horizontal lines indicate the 95% CI of the odds ratio (OR) estimate in each study. The diamond represents the pooled point estimate, and its width represents the pooled estimate 95% CI.

1. **Secondary outcome** - Effect of statins on variceal bleeding: Meta-analysis of unadjusted odds ratios from RCTs and observational studies.


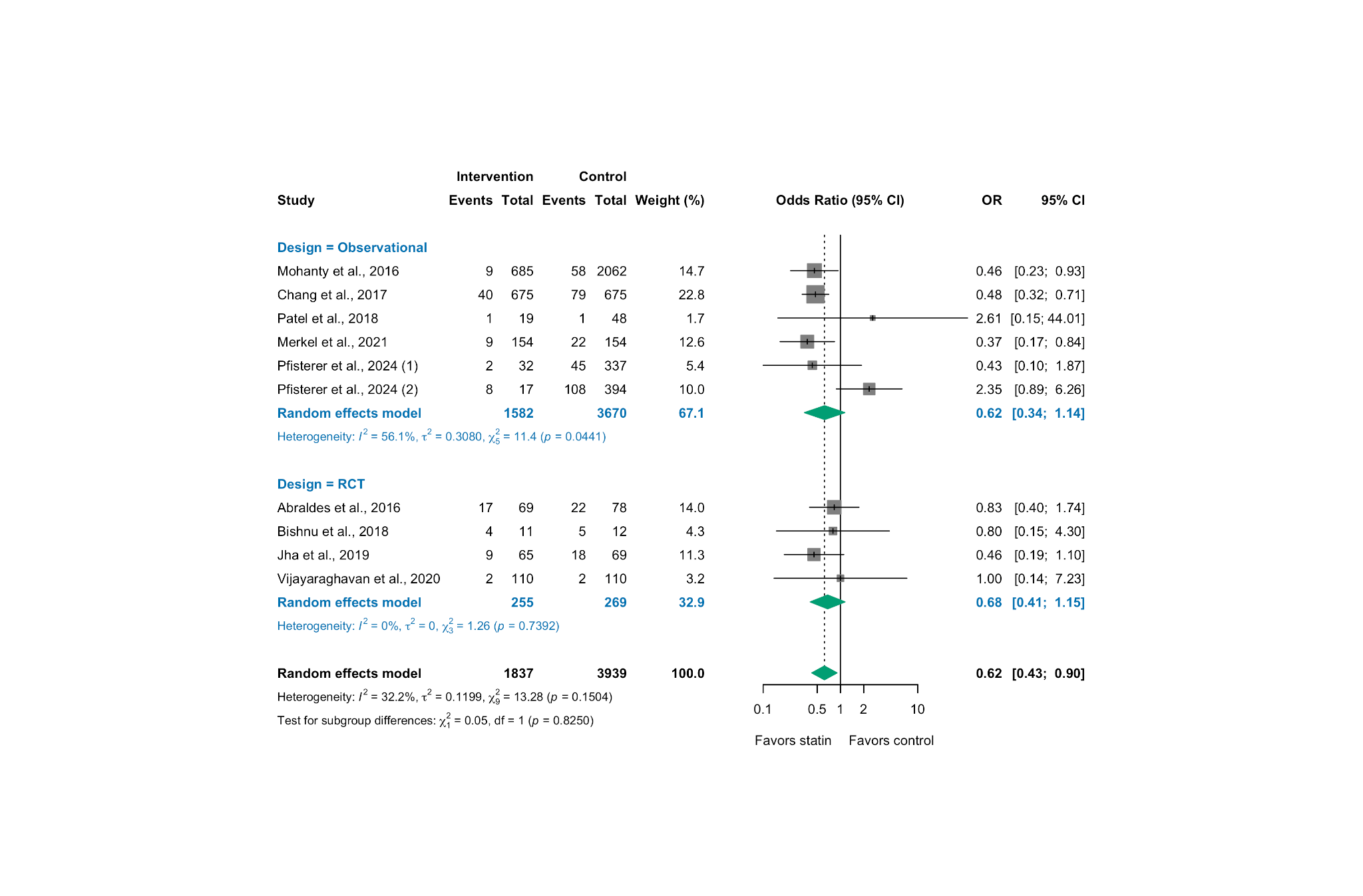


The squares represent point estimates, and the size of each square is proportional to the weight of the study. Horizontal lines indicate the 95% CI of the odds ratio (OR) estimate in each study. The diamond represents the pooled point estimate, and its width represents the pooled estimate 95% CI. Summary effects are presented separately for the RCTs and observational studies, as well as for the overall combined analysis.

1. **Secondary outcome** - Effect of statins on ascites: Meta-analysis of unadjusted odds ratios from RCTs and observational studies.


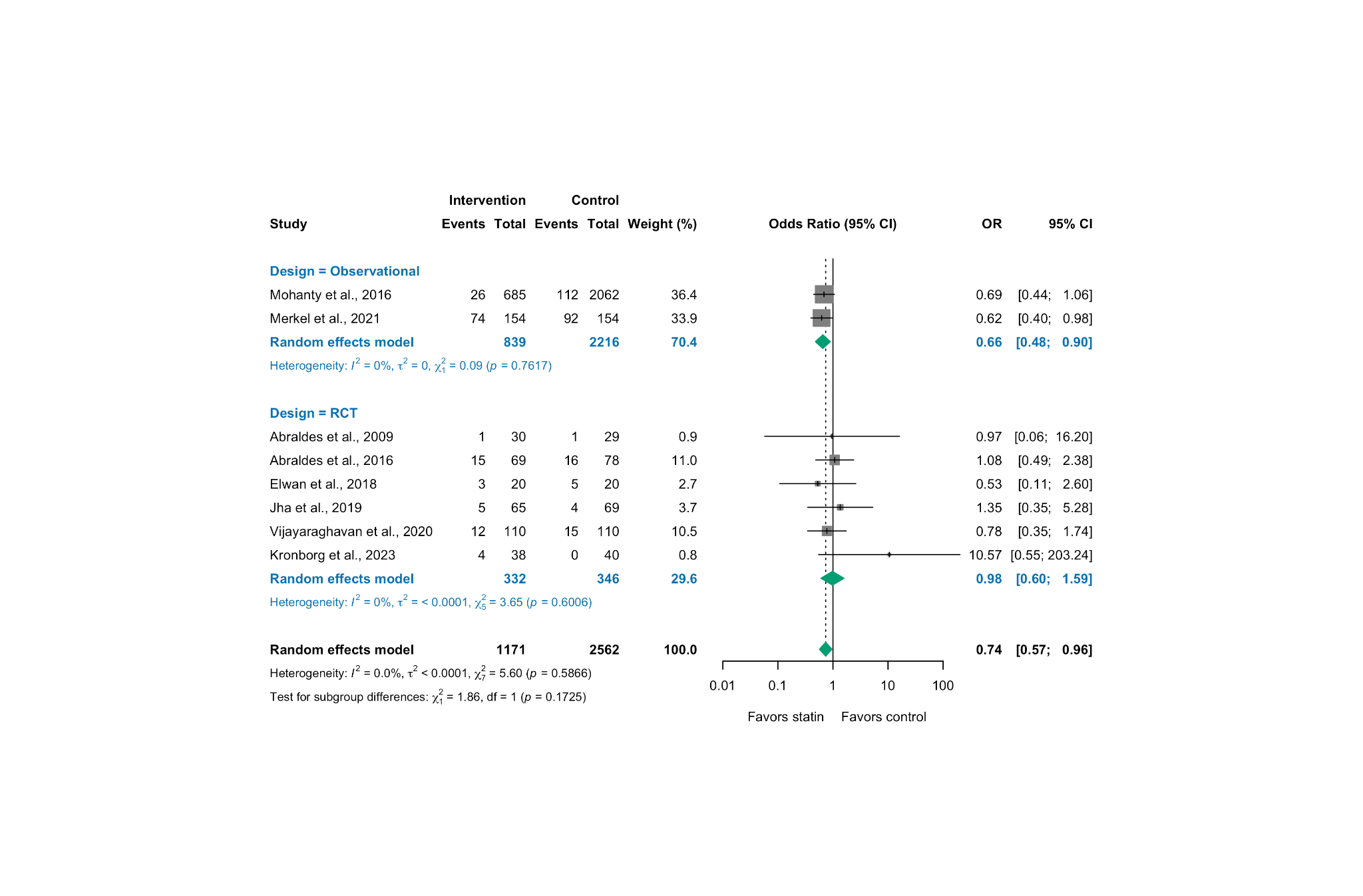


The squares represent point estimates, and the size of each square is proportional to the weight of the study. Horizontal lines indicate the 95% CI of the odds ratio (OR) estimate in each study. The diamond represents the pooled point estimate, and its width represents the pooled estimate 95% CI. Summary effects are presented separately for the RCTs and observational studies, as well as for the overall combined analysis.

1. **Secondary outcome** - Effect of statins on hepatorenal syndrome: Meta-analysis of unadjusted odds ratios from RCTs and observational studies.


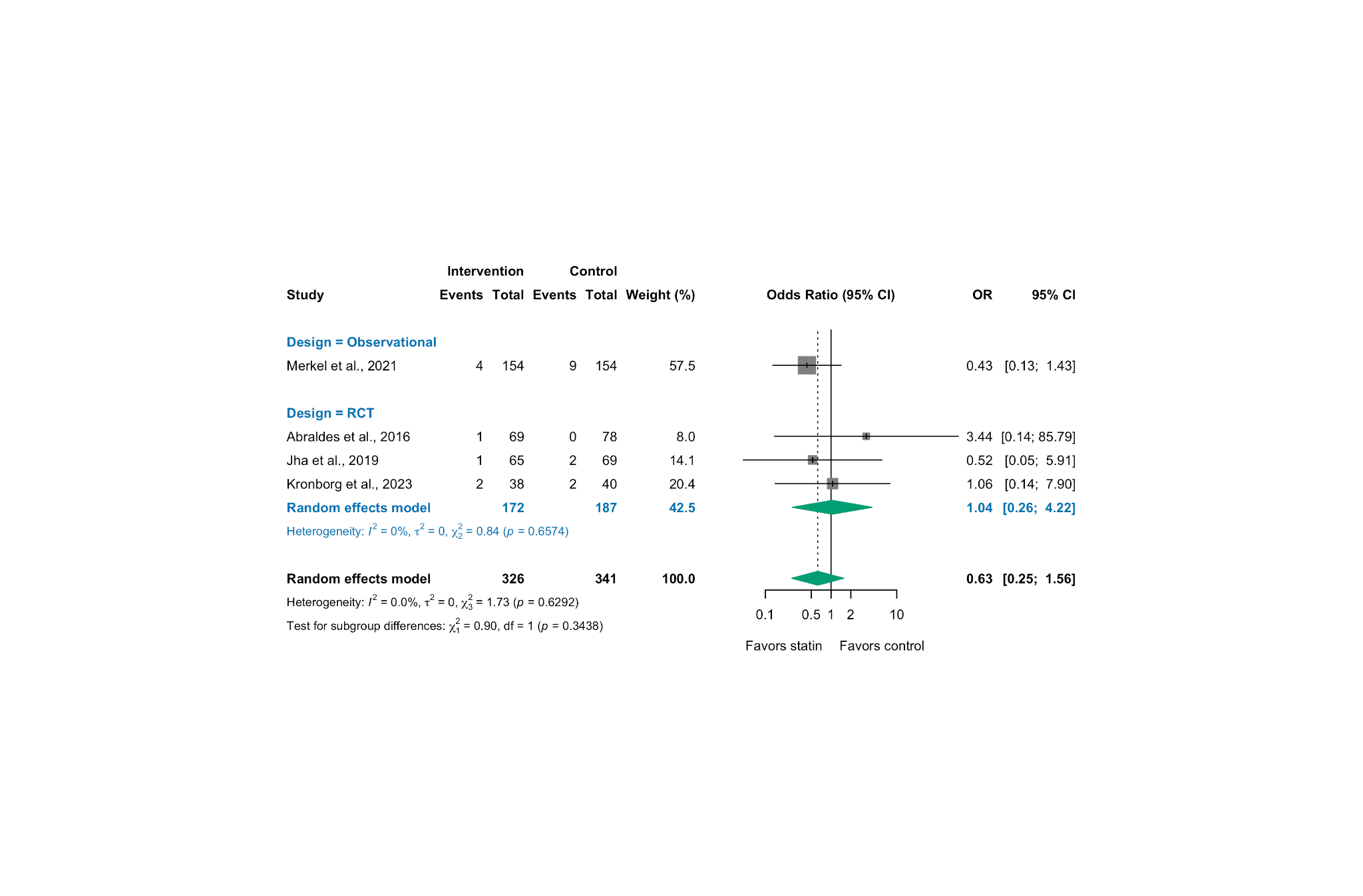


The squares represent point estimates, and the size of each square is proportional to the weight of the study. Horizontal lines indicate the 95% CI of the odds ratio (OR) estimate in each study. The diamond represents the pooled point estimate, and its width represents the pooled estimate 95% CI. Summary effects are presented for the RCTs subgroup and for the overall combined analysis. As only one observational study was included, its individual estimate is shown without a separate subgroup summary.

1. **Secondary outcome** - Effect of statins on spontaneous bacterial peritonitis: Meta-analysis of unadjusted odds ratios from RCTs and observational studies.


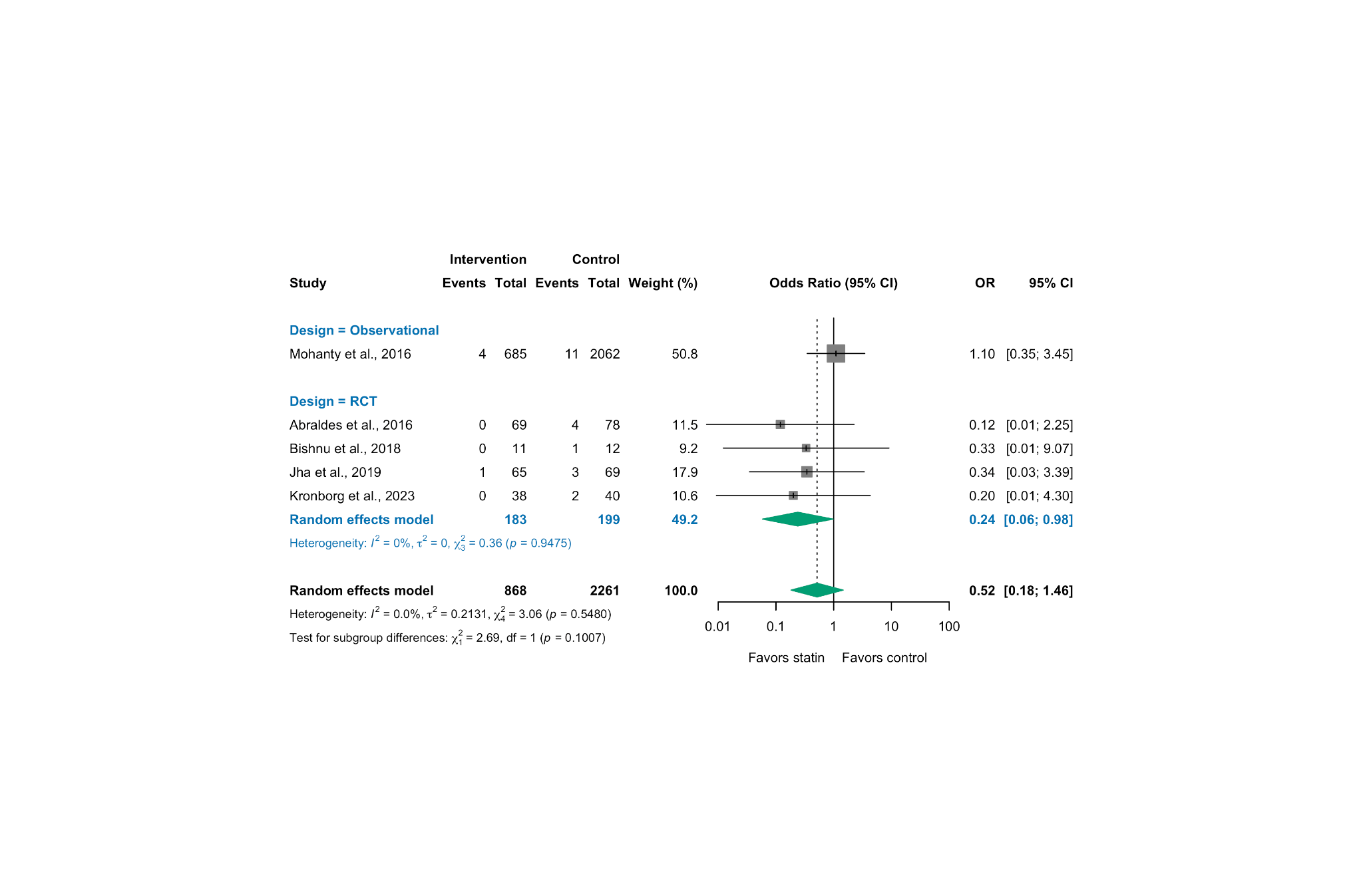


The squares represent point estimates, and the size of each square is proportional to the weight of the study. Horizontal lines indicate the 95% CI of the odds ratio (OR) estimate in each study. The diamond represents the pooled point estimate, and its width represents the pooled estimate 95% CI. Summary effects are presented for the RCTs subgroup and for the overall combined analysis. As only one observational study was included, its individual estimate is shown without a separate subgroup summary.

1. **Secondary outcome** - Effect of statins on hepatic encephalopathy: Meta-analysis of unadjusted odds ratios from RCTs and observational studies.


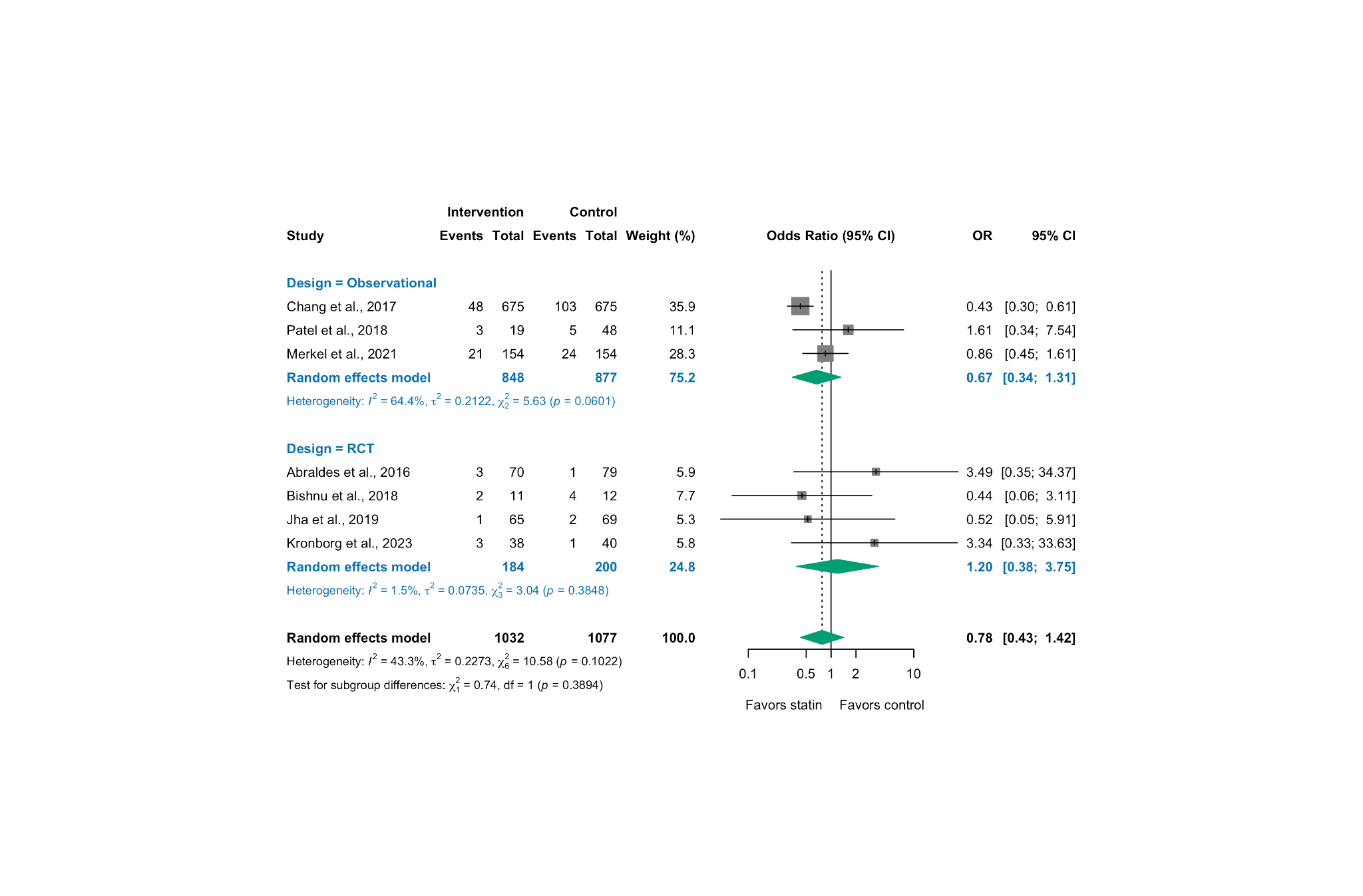


The squares represent point estimates, and the size of each square is proportional to the weight of the study. Horizontal lines indicate the 95% CI of the odds ratio (OR) estimate in each study. The diamond represents the pooled point estimate, and its width represents the pooled estimate 95% CI. Summary effects are presented separately for the RCTs and observational studies, as well as for the overall combined analysis.

**eFigure 4:** Sensitivity analysis of RCTs using relative risk.

1. **Primary outcome** - Effect of statins on all-cause mortality: Meta-analysis of relative risks from RCTs.


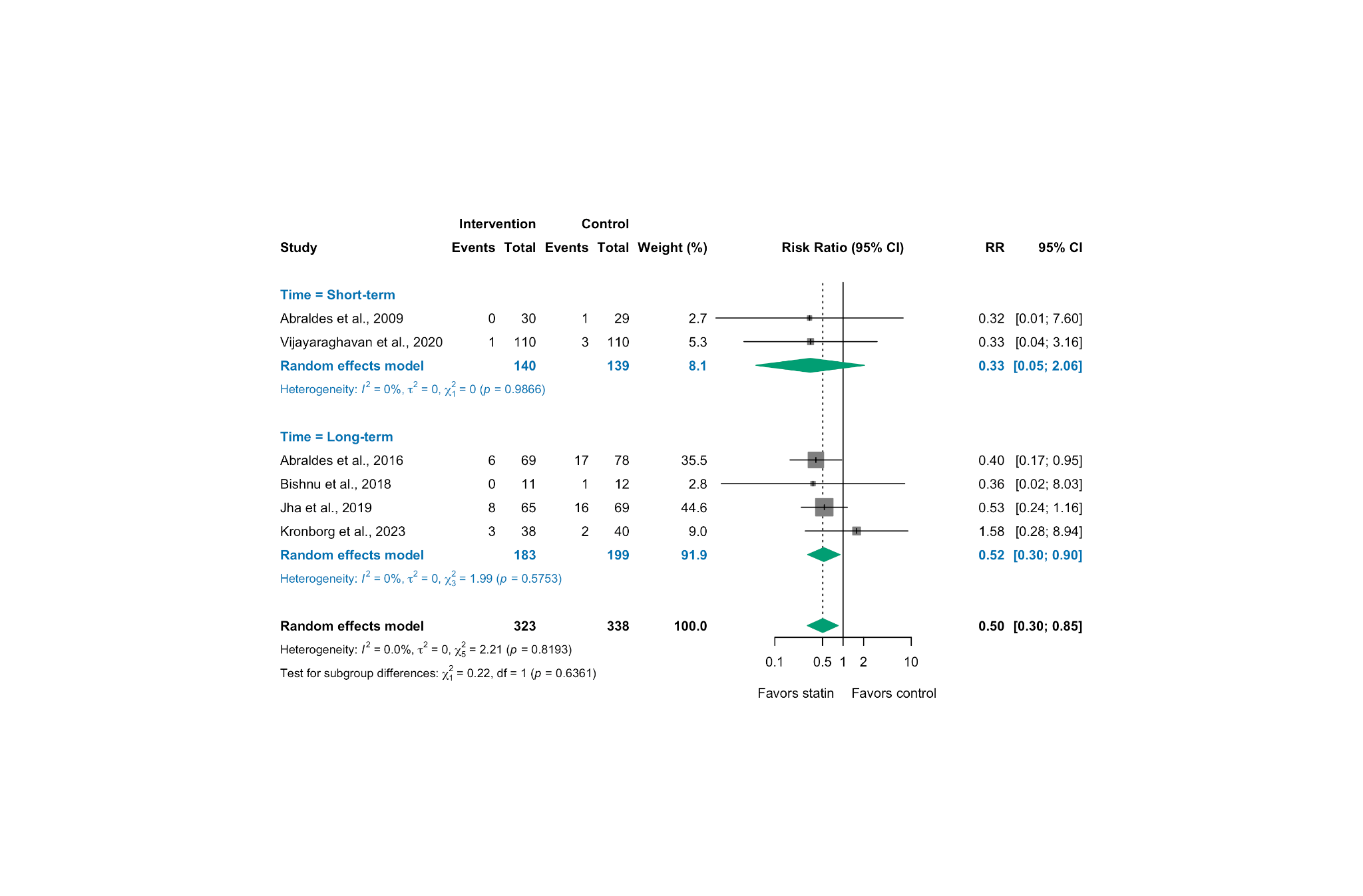


The squares represent point estimates, and the size of each square is proportional to the weight of the study. Horizontal lines indicate the 95% CI of the relative risk (RR) estimate in each study. The diamond represents the pooled point estimate, and its width represents the pooled estimate 95% CI. Summary effects are presented separately for the short-term and long-term follow-up subgroups, as well as for the overall combined analysis.

1. **Primary outcome** - Effect of statins on hepatic decompensation: Meta-analysis of relative risks from RCTs.


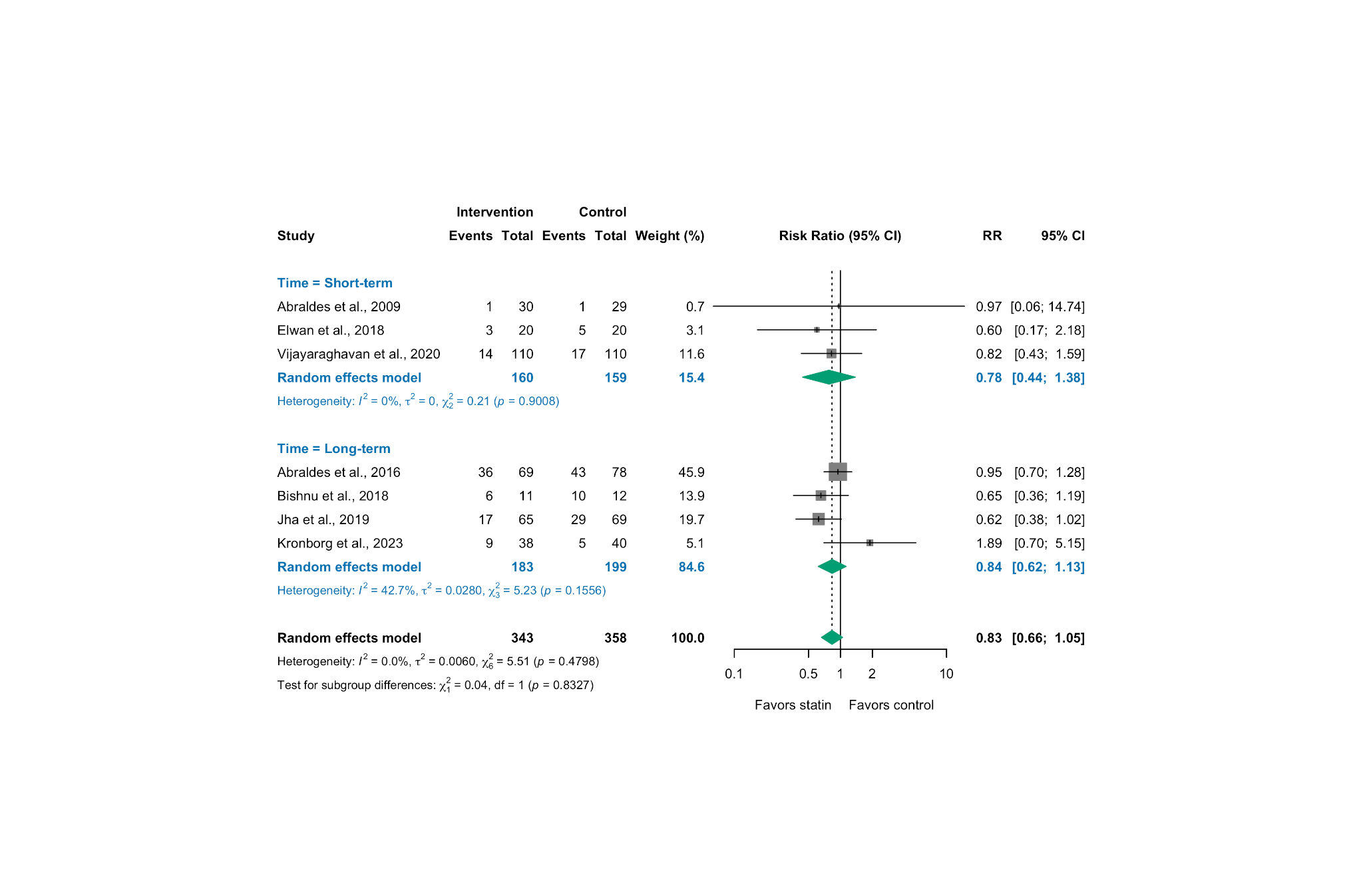


The squares represent point estimates, and the size of each square is proportional to the weight of the study. Horizontal lines indicate the 95% CI of the relative risk (RR) estimate in each study. The diamond represents the pooled point estimate, and its width represents the pooled estimate 95% CI. Summary effects are presented separately for the short-term and long-term follow-up subgroups, as well as for the overall combined analysis.

1. **Secondary outcome** - Effect of statins on variceal bleeding: Meta-analysis of relative risks from RCTs.


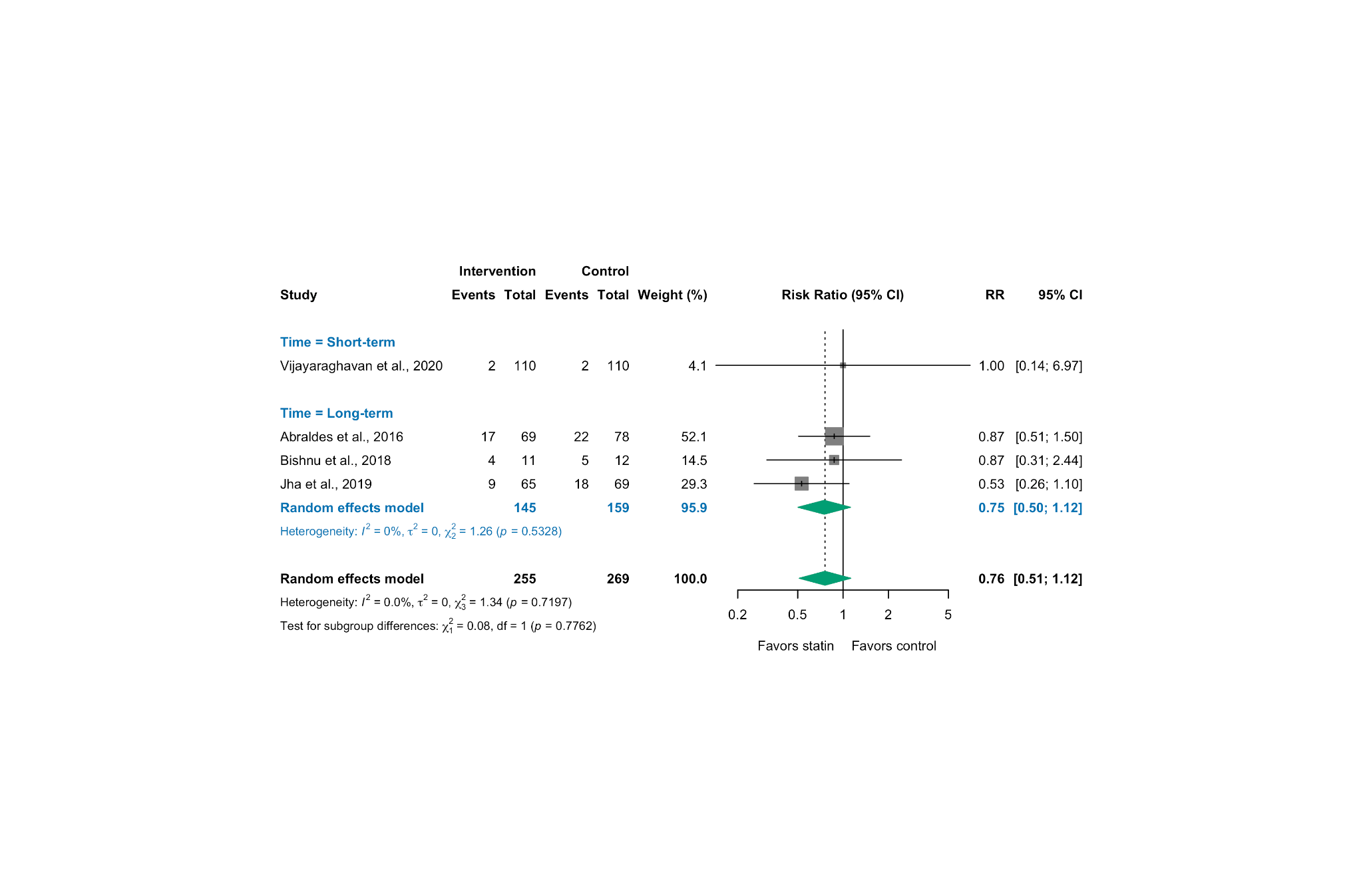


The squares represent point estimates, and the size of each square is proportional to the weight of the study. Horizontal lines indicate the 95% CI of the relative risk (RR) estimate in each study. The diamond represents the pooled point estimate, and its width represents the pooled estimate 95% CI. Summary effects are presented for the long-term follow-up subgroup and for the overall combined analysis. As only one short-term follow-up study was included, its individual estimate is shown without a separate subgroup summary.

1. **Secondary outcome** - Effect of statins on ascites: Meta-analysis of relative risks from RCTs.


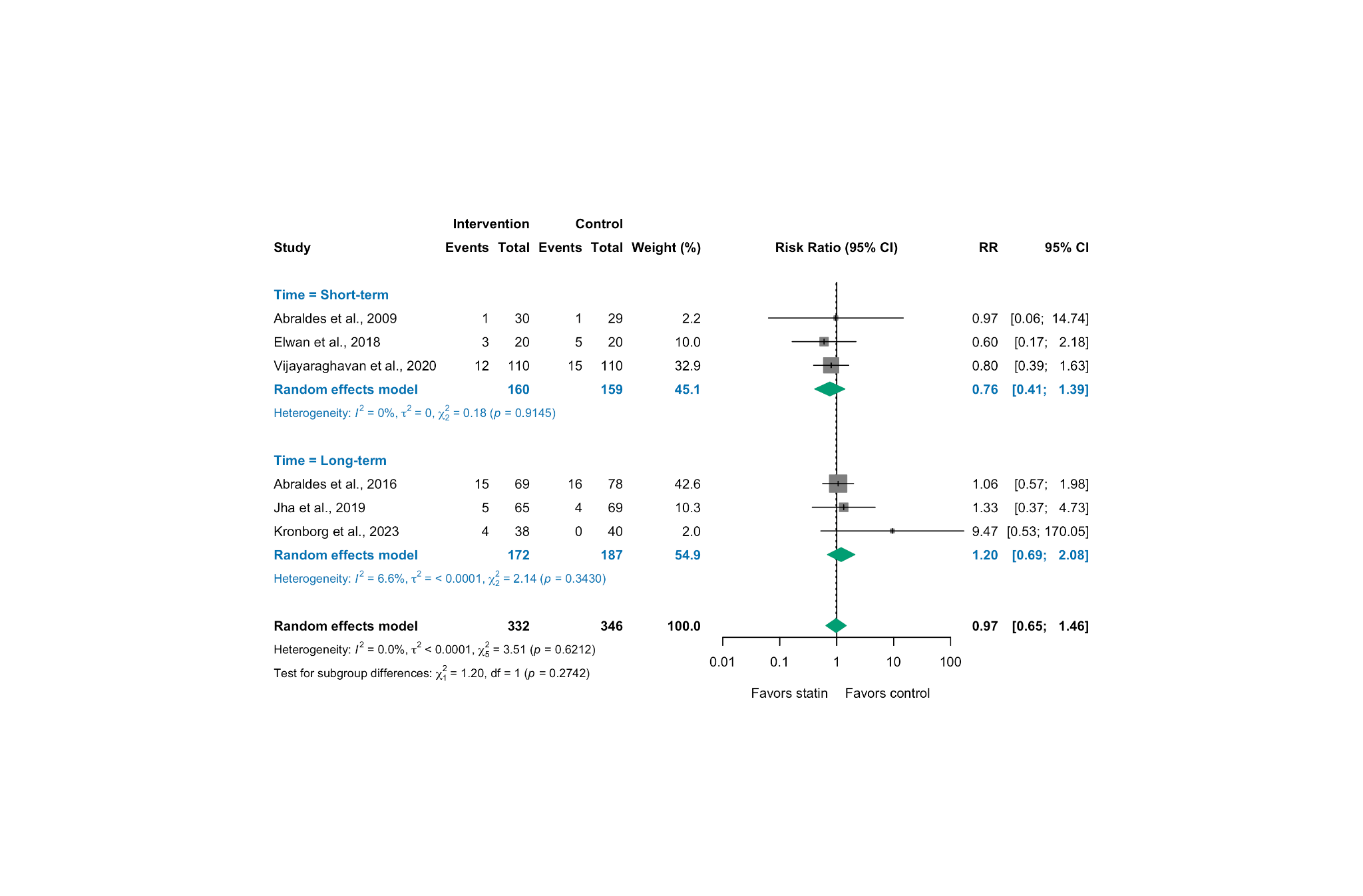


The squares represent point estimates, and the size of each square is proportional to the weight of the study. Horizontal lines indicate the 95% CI of the relative risk (RR) estimate in each study. The diamond represents the pooled point estimate, and its width represents the pooled estimate 95% CI. Summary effects are presented separately for the short-term and long-term follow-up subgroups, as well as for the overall combined analysis.

1. **Secondary outcome** - Effect of statins on hepatorenal syndrome: Meta-analysis of relative risks from RCTs.


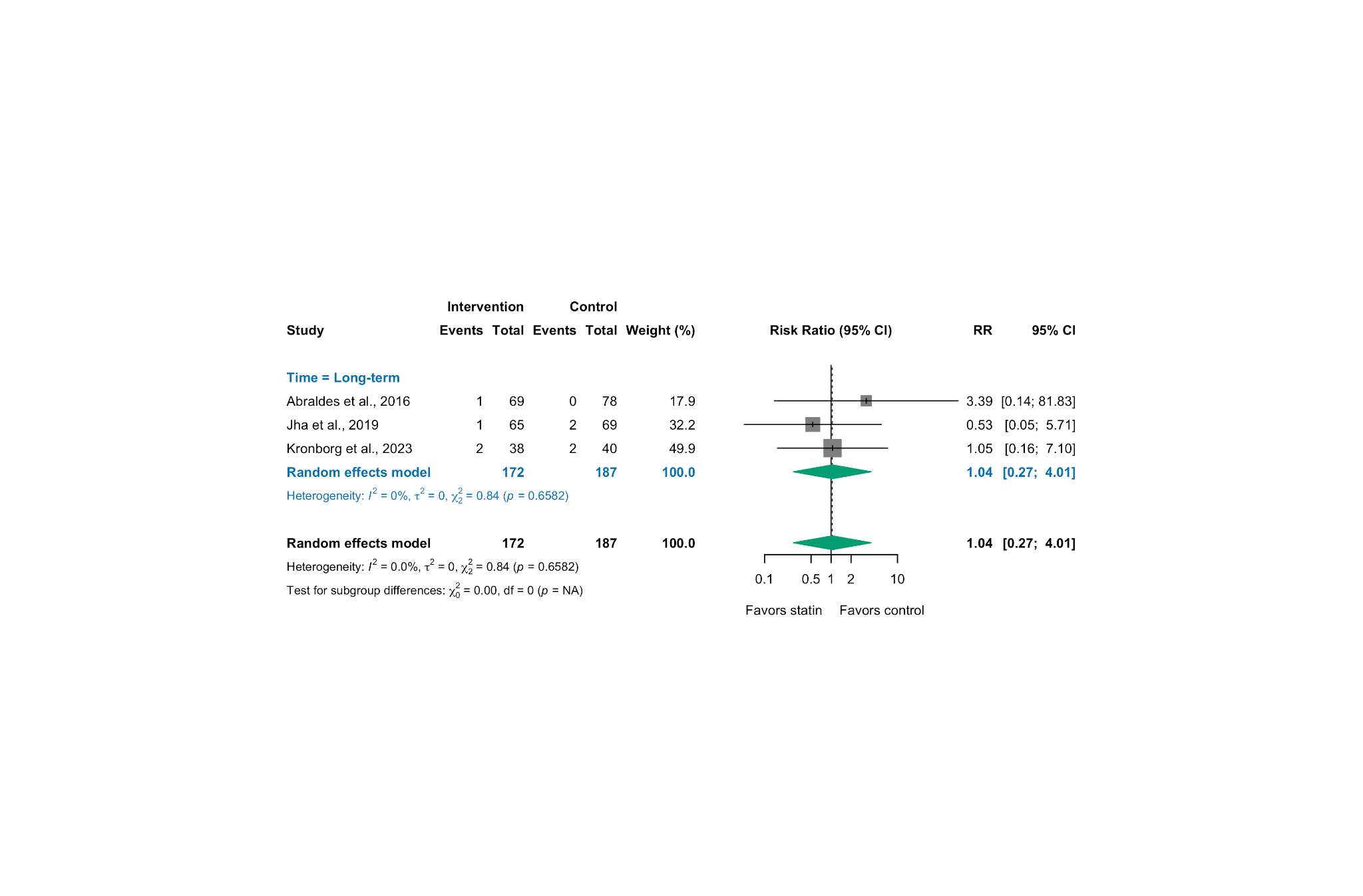


The squares represent point estimates, and the size of each square is proportional to the weight of the study. Horizontal lines indicate the 95% CI of the relative risk (RR) estimate in each study. The diamond represents the pooled point estimate, and its width represents the pooled estimate 95% CI. As all included trials were classified under the long-term follow-up subgroup, the summary effect shown represents both this subgroup and the overall pooled estimate.

1. **Secondary outcome** - Effect of statins on spontaneous bacterial peritonitis: Meta-analysis of relative risks from RCTs.


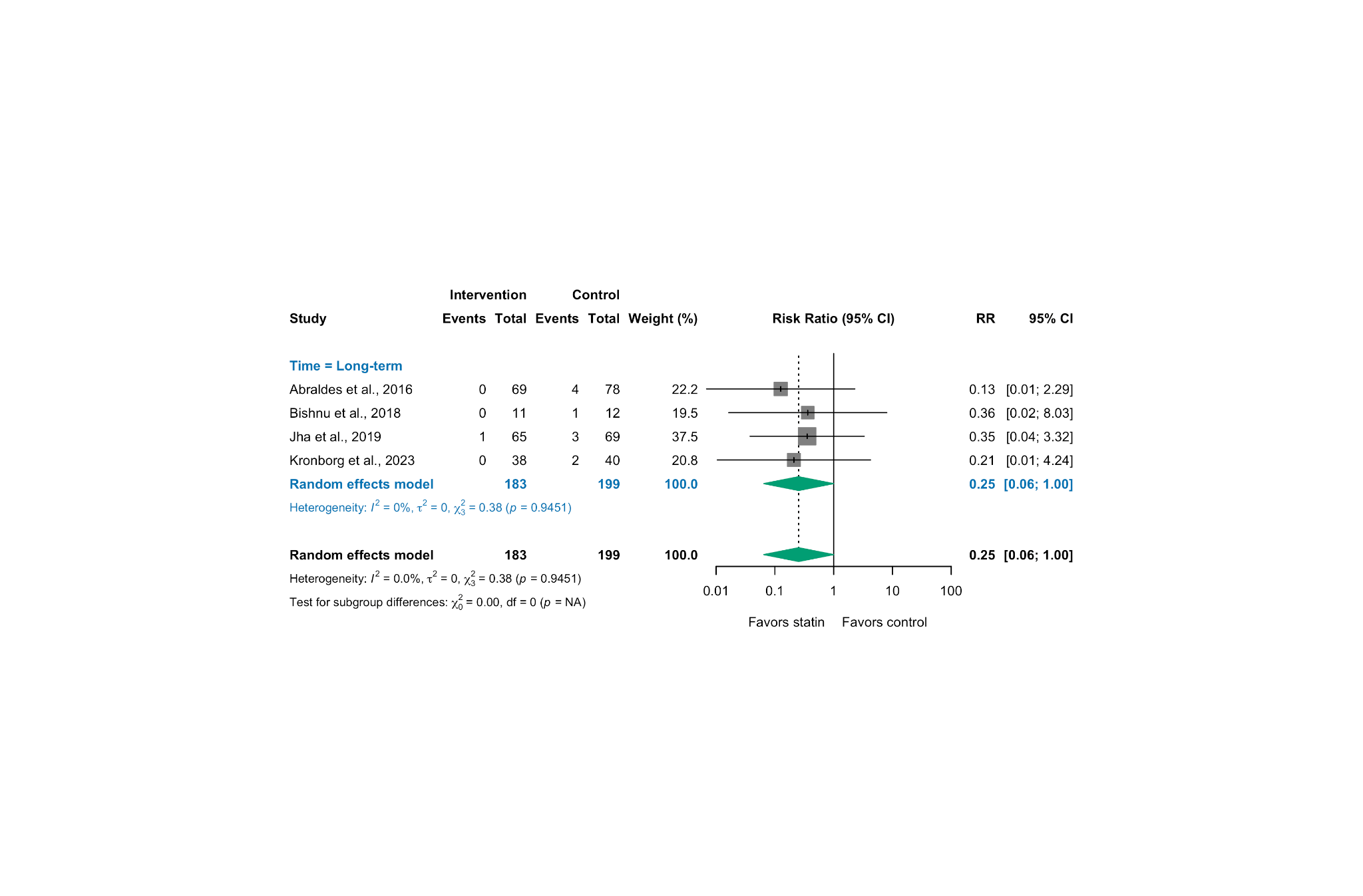


The squares represent point estimates, and the size of each square is proportional to the weight of the study. Horizontal lines indicate the 95% CI of the relative risk (RR) estimate in each study. The diamond represents the pooled point estimate, and its width represents the pooled estimate 95% CI. As all included trials were classified under the long-term follow-up subgroup, the summary effect shown represents both this subgroup and the overall pooled estimate.

1. **Secondary outcome** - Effect of statins on hepatic encephalopathy: Meta-analysis of relative risks from RCTs.


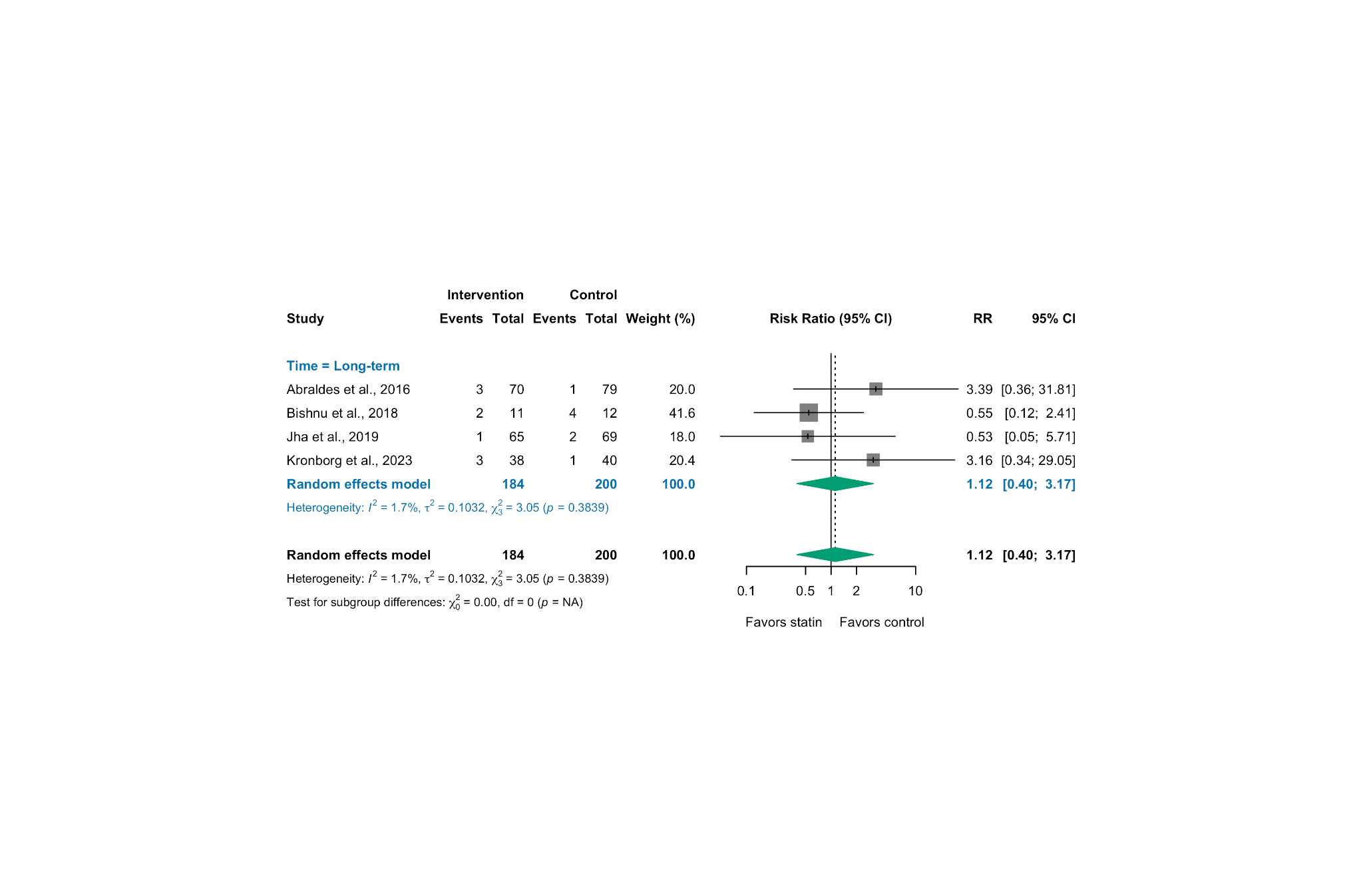


The squares represent point estimates, and the size of each square is proportional to the weight of the study. Horizontal lines indicate the 95% CI of the relative risk (RR) estimate in each study. The diamond represents the pooled point estimate, and its width represents the pooled estimate 95% CI. As all included trials were classified under the long-term follow-up subgroup, the summary effect shown represents both this subgroup and the overall pooled estimate.

**eFigure 5:** Post hoc sensitivity analysis of the effect of statins on change in HVPG restricted to RCTs in which all participants received non-selective beta-blockers.


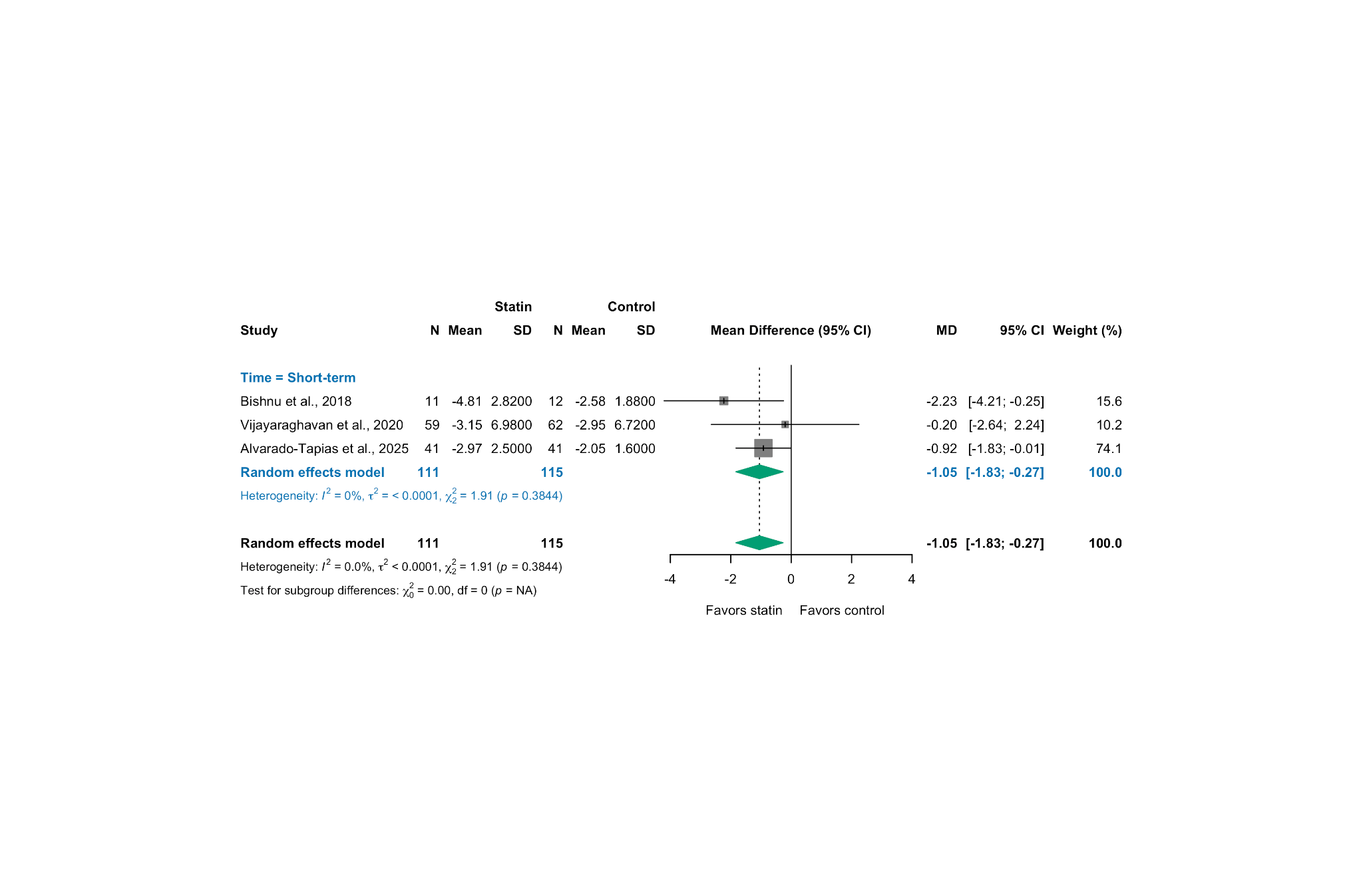


The squares represent point estimates, and the size of each square is proportional to the weight of the study. Horizontal lines indicate the 95% CI of the mean difference (MD) estimate in each study. The diamond represents the pooled point estimate, and its width represents the pooled estimate 95% CI. As all included trials were classified under the short-term follow-up subgroup, the summary effect shown represents both this subgroup and the overall pooled estimate.

**eFigure 6:** Funnel Plots.

1. Primary outcome: All-cause Mortality; RCTs and observational studies combined.


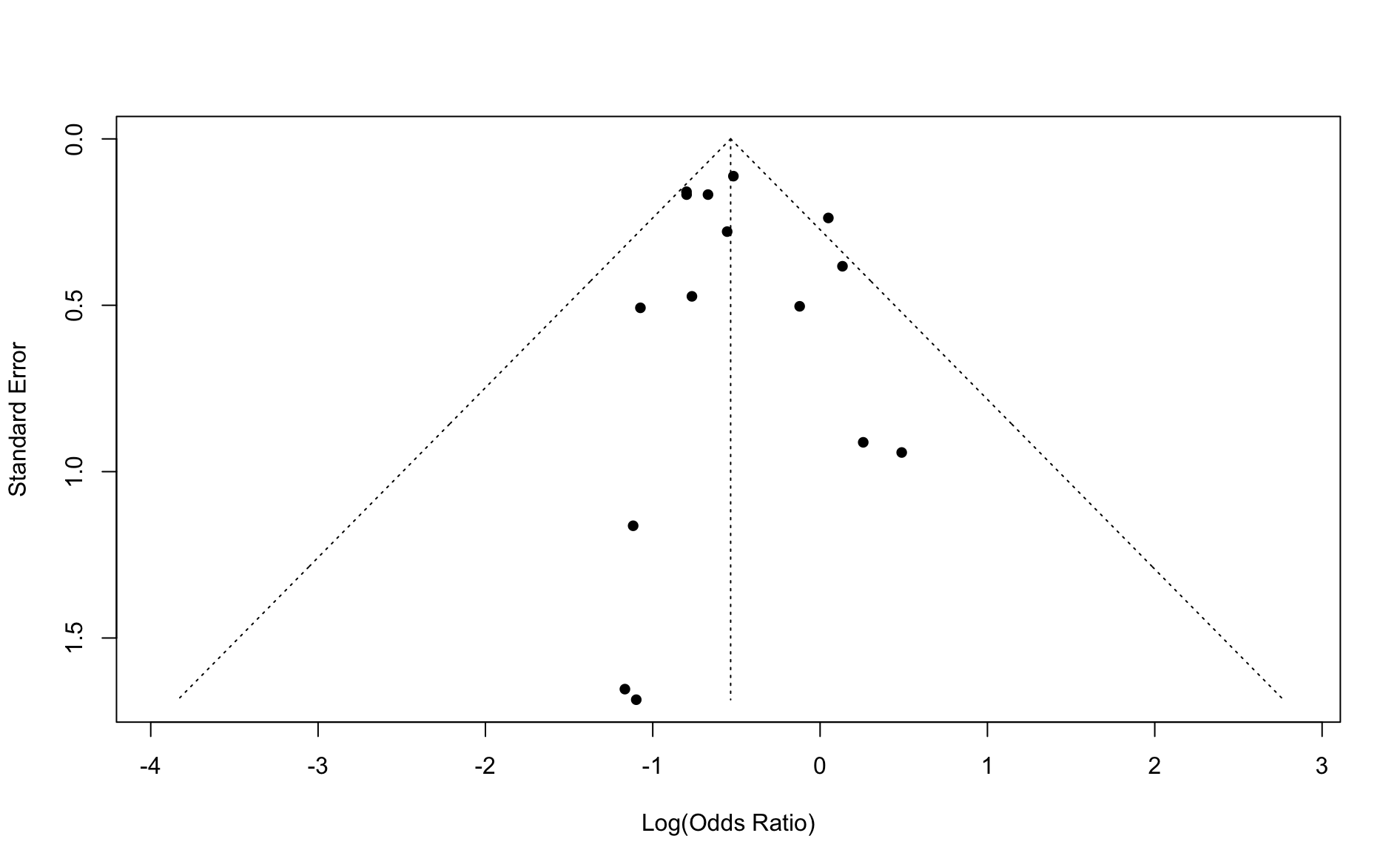


Funnel plot with pseudo 95% confidence intervals.

1. Primary outcome: Hepatic decompensation; RCTs and observational studies combined.


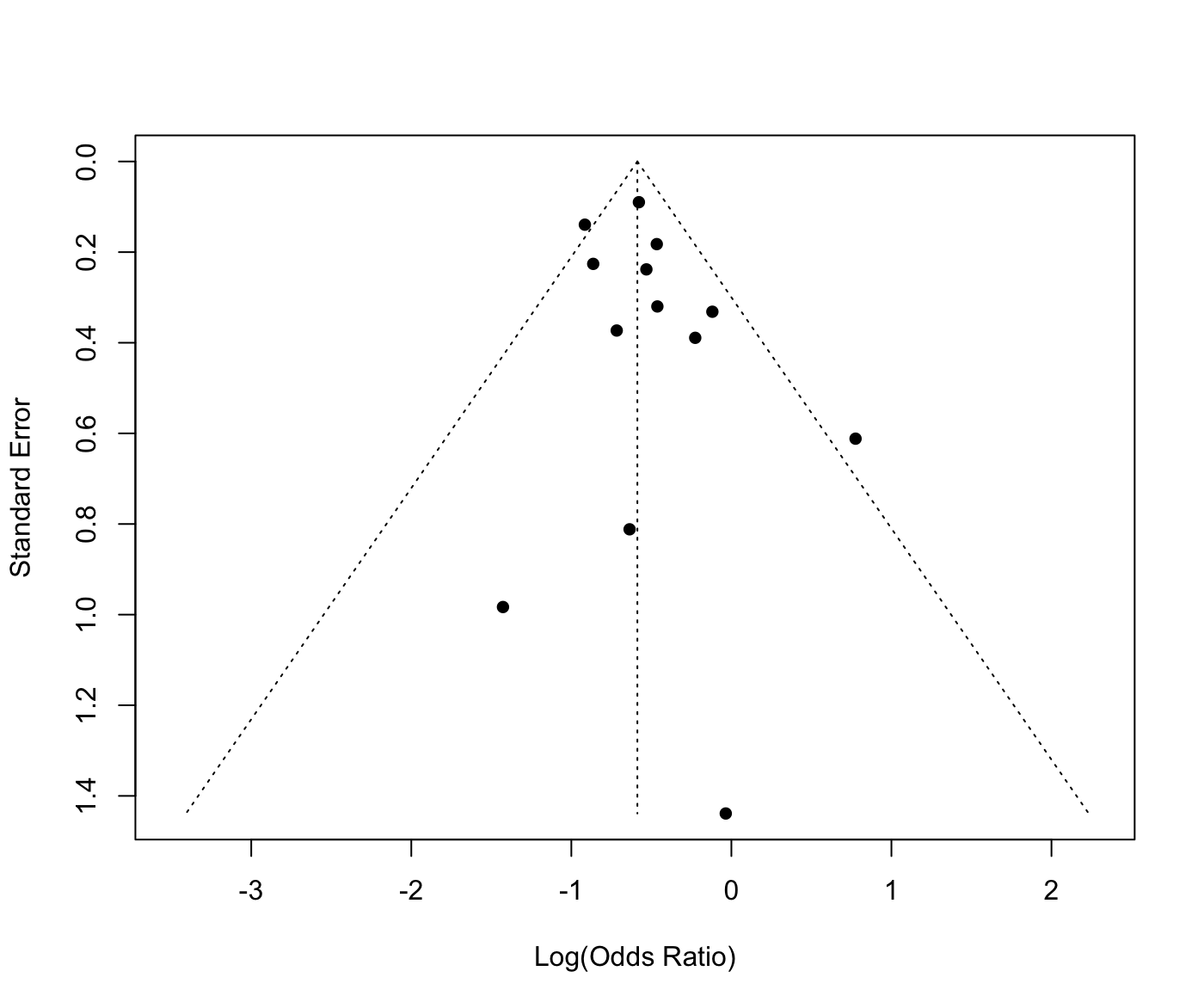


Funnel plot with pseudo 95% confidence intervals.
